# Scrolling in Supramolecular Gels: A Designer’s Guide

**DOI:** 10.1021/acs.chemmater.3c03013

**Published:** 2024-03-09

**Authors:** Christopher D. Jones, Laurence J. Kershaw Cook, Anna G. Slater, Dmitry S. Yufit, Jonathan W. Steed

**Affiliations:** † Department of Chemistry, Durham University, Durham DH1 3LE, U.K.; ‡ Department of Chemistry and Materials Innovation Factory, University of Liverpool, Crown Street, Liverpool L69 7ZD, U.K.

## Abstract

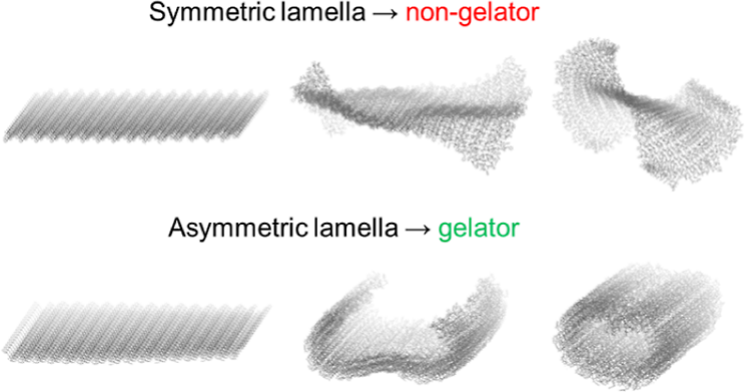

Gelation by small molecules is a topic of enormous importance in catalysis, nanomaterials, drug delivery, and pharmaceutical crystallization. The mechanism by which gelators self-organize into a fibrous gel network is poorly understood. Herein, we describe the crystal structures and gelation properties of a library of bis(urea) compounds and show, via molecular dynamics simulations, how gelator aggregation progresses from a continuous pattern of supramolecular motifs to a homogeneous fiber network. Our model suggests that lamellae with asymmetric surfaces scroll into uniform unbranched fibrils, while sheets with symmetric surfaces undergo stacking to form crystals. The self-assembly of asymmetric lamellae is associated with specific molecular features, such as the presence of narrow and flexible end groups with high packing densities, and likely represents a general mechanism for the formation of small-molecule gels.

## Introduction

Low molecular weight gelators (LMWGs) are a diverse group of compounds capable of forming extended networks of supramolecular polymers.^1−6^ These polymers typically coalesce into extended tubular or helical structures, known as fibrils, which develop into larger fibers through braiding or entanglement.^7,8^ The gels resulting from these self-assembled structures may replace polymeric materials in applications such as drug delivery, crystal growth, and chemical sensing.^6,9−11^ LMWGs often provide more reproducible performance than polymeric gelators, which are sensitive to variations in molecular weight, branching, and degree of functionalization. As small molecules, they may offer a greater scope for synthetic modification, allowing gels to be prepared with tunable rheological characteristics and improved compatibility with target solvents. Moreover, the reversibility of the self-assembly process means that gel–sol transitions can be induced as required, by disrupting labile supramolecular motifs with heat, light or chemical stimuli.^12,13^

Although the structures of small-molecule gels have been investigated in detail, designing new LMWGs remains a challenge.^14−16^ Useful insights have been drawn from comparisons of successful LMWG-solvent pairings, including predictive correlations between empirical solvent parameters and the size and polarity of gelator end groups.^17^ Nonetheless, it is difficult to identify an LMWG prior to experimental testing or explain the effects of even small synthetic modifications on the gelation ability. Functional groups may be chosen to favor a key fibril-forming supramolecular motif, but competing interactions or the self-assembly of alternative structures, such as crystals, may disrupt the potential for gel formation.^18−21^

The molecular packing observed when a compound forms a gel often differs from the arrangement observed in its crystal structures. For this reason, it has been argued that the analysis of crystals may not usefully inform the design of LMWGs.^20,22−25^ In other cases, there is a clear relationship between crystal structure and gel chemistry.^26−28^ It has also been demonstrated that molecules displaying one-dimensional supramolecular motifs in their crystals are far more likely to give rise to gels.^16^ Likewise, other supramolecular tendencies, such as the formation of helical or lamellar hydrogen bonding networks, can be considered topological features that will be strongly conserved even when the molecular packing is otherwise altered.^29−32^ Identifying such structures and simulating their behavior under different aggregation conditions could offer insight into the mechanism of gelator self-assembly and allow the outcome of this process to be more easily predicted and controlled.

**Figure 1:**
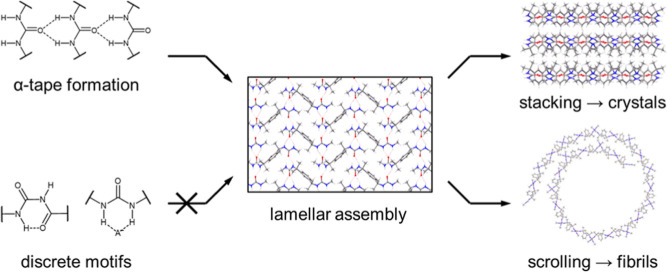
Schematic illustration of urea crystallization and fibril formation via the stacking and scrolling of lamellar assemblies. Lamellae form from continuous, two-dimensional networks of α-tapes, which may be disrupted by the formation of discrete hydrogen bonding motifs.^18^

Along with amides, urea derivatives are among the most common LMWGs and are good representative examples of gel-forming systems in which directional hydrogen bonding is thought to be important.^3,8,33,34^ Gelation by ureas is typically associated with the formation of a continuous array of hydrogen bonds known as an α-tape motif.^8,35^ In bis(urea) systems, molecules may assemble into two-dimensional α-tape networks or lamellae (Figure 1). We have previously shown that bis(urea) lamellae with asymmetric faces may, like other polar sheet-like structures, scroll spontaneously into cylindrical fibrils.^18^ For a gel to form, the lamellae must grow and scroll before stacking can occur. Competition between gelation and crystallization processes is therefore common and may be strongly influenced by environmental variables such as temperature, pH, and solvent composition.^20,36,37^ The likelihood of gelation and the type of gel formed can also be highly dependent on the sequence of conditions to which a sample is exposed, resulting in even greater pathway complexity.^38^

When designing an LMWG, the choice of the end group is highly important. For example, end groups containing strong hydrogen bond acceptors such as anions, pyridines, and carboxylic acids are often found to reduce the gelation capacities of bis(urea) gelators by inhibiting the formation of the α-tape structure.^27,39^ In some systems, however, species of this type may facilitate gel formation by giving rise to additional continuous hydrogen bonding motifs^40,41^ or link molecules into dimeric assemblies with improved gelation properties.^42^ Our lamellar scrolling model suggests that gel formation is also more likely if the end groups are small and flexible, allowing them to adopt a densely packed arrangement on a single face of the parent lamella.

The aim of this investigation was to test the utility of the lamellar scrolling model by synthesizing a library of potential gelators with different end groups and determining whether their gelation capacities are correlated to their single-crystal structures. As in our previous study,^18^ bis(urea)s derived from tetramethylxylylene diisocyanate (otherwise named 1,3-bis(1-isocyanato-1-methylethyl)benzene)^43−46^ were found to crystallize readily from polar solvents and form a range of hydrogen bonding networks. Derivatives featuring asymmetric lamellae in their crystal structures, and lacking alternative nonlamellar crystal forms, were likely to produce gels in aromatic solvents. The potential for scrolling was demonstrated via molecular dynamics (MD) simulations of isolated assemblies in the gas phase. The study illustrates how the analysis of crystal structures can aid the development of effective LMWGs, by highlighting general trends in the arrangement of supramolecular motifs and suggesting likely mechanisms by which fibrils may arise.

## Results and Discussion

### Synthesis and Characterization

Bis(urea)s **1**–**10** (Scheme 1) with different end groups were prepared by reacting tetramethylxylylene diisocyanate with a small excess of an amine. Compounds **1d** and **5a**, and those of type **11**, have been previously reported along with their crystal structures.^18,47,48^ Most of the compounds precipitate on formation and can be purified by washing with chloroform. Compound **5c**, however, is soluble in chloroform and was therefore isolated by evaporation of the solvent in vacuo. The products were characterized by NMR spectroscopy, mass spectrometry, and elemental (CHN) analysis (Supporting Information, Figures S1–S90).

**Scheme 1:**
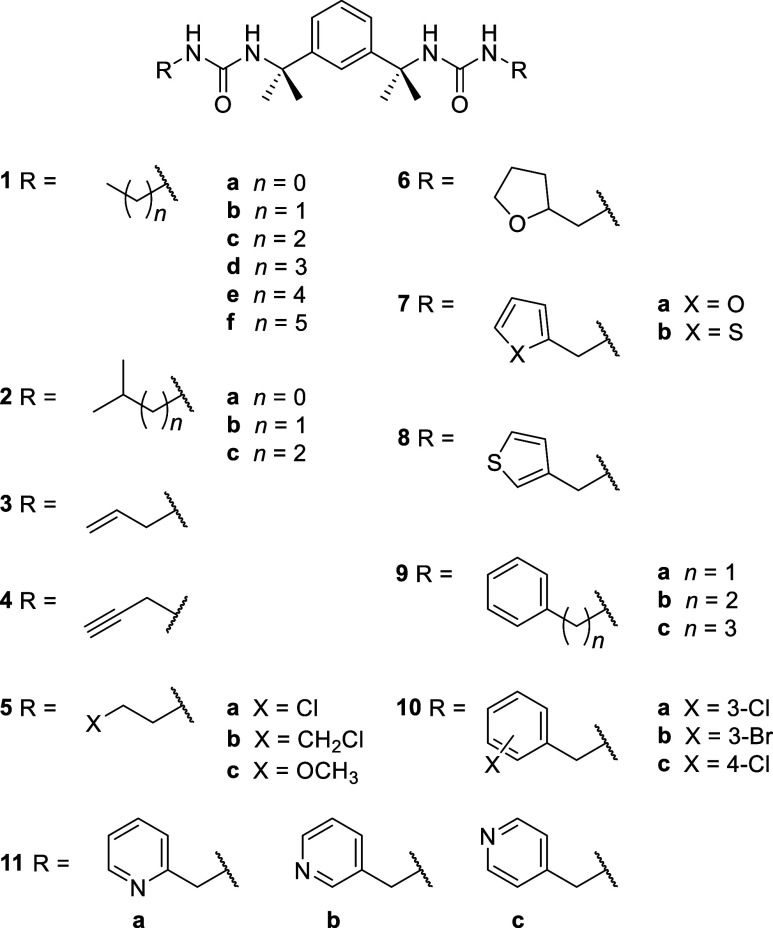
Bis(urea) Compounds Prepared in the Present Work

Recrystallization of the compounds was performed by slow cooling hot solutions of the compounds in methanol, ethanol, 1-propanol, or acetonitrile and yielded crystals suitable for single-crystal X-ray diffraction analysis for all derivatives except **1e**, **1f,** and **8** (Table S11). Compound **6** was synthesized as a mixture of diastereomers, but a crystal structure was acquired for the *meso* form only. For compounds **2a**, **4**, **5b,** and **7b**, two polymorphs were obtained and labeled Forms 1 and 2 in each case. The Form 1 structures of **4**, **5b,** and **7b** crystallized reproducibly from methanol, while the Form 2 polymorphs were produced from ethanol, acetonitrile, and 1-propanol, respectively. Both polymorphs of **2a** were crystallized from methanol, but the monoclinic Form 1 structure was found to be a “disappearing” polymorph:^49,50^ the material was obtained only once by slow recrystallization, and all subsequent trials in methanol and other solvents always yielded the tetragonal Form 2. Refinement parameters for the collected data sets were generally satisfactory, but the crystal structures of **5b** (Form 1), *meso*-**6,** and **10c** were of relatively low precision, as large, good-quality crystals of these materials could not be obtained. Nonetheless, the overall structural details are unambiguous.

### Structural Trends

We have previously performed a survey of urea-containing crystal structures in the Cambridge Structural Database (CSD).^18^ These structures (Supporting Information, Tables S1–S10) display a range of tape motifs, which are categorized as α-tapes if they comprise only well-defined *R*_2_^1^(6) urea–urea synthons (Supporting Information, Figure S91).^51−53^ Perhaps surprisingly, only 648 (41%) of the 1568 structures display urea–urea interactions, and tape motifs occur in just 385 (25%). Tapes are particularly rare in the 20% of structures containing urea groups in the *syn*-*anti* conformation, which typically self-assemble into discrete dimers rather than continuous hydrogen bonding motifs.^32,54,55^ In the remaining structures, where urea groups adopt the *syn*–*syn* conformation, the absence of α-tapes is often attributable to the existence of competing interactions with guest species (Supporting Information, Figure S92).^13,56−58^ For example, ions are observed in 37% of the *syn*–*syn* structures lacking tape motifs, but only 4.2% of the *syn*–*syn* structures in which tapes are present. The frequency of the *syn*–*syn* conformation, and thus the probability of α-tape formation, is greatest for urea groups linked to alkyl substituents rather than heteroatoms, carbonyls, or aryl groups. The use of the tetramethylxylylene spacer, in combination with relatively weakly interacting space groups, was therefore a reasonable strategy for generating a library of materials featuring α-tape motifs.

The bis(urea) crystals in this study display α-tape networks with several different repeat units. As in our previous investigation,^18^ the structure of the repeat unit may be described by labeling each molecule with a letter, such that molecules assigned the same letter are involved in the same pair of α-tapes. For example, the repeat unit [AB] displays a lamellar, “brick-wall” structure, wherein α-tapes are shared between molecules in alternating rows. Of the 26 crystal structures determined for bis(urea)s **1**–**10**, 21 display lamellar α-tape networks with [AB] or [AABB] repeat units. The remaining structures are nonlamellar, as the bis(urea) molecules are linked by α-tapes into a three-dimensional network. The structures of **2a** (Form 2), **3**, **5a** (Form 2), and **5b** (Form 2) comprise the repeat unit [ABCD], while **10c** adopts a more complex [ABABCDCD] arrangement.

**Figure 2:**
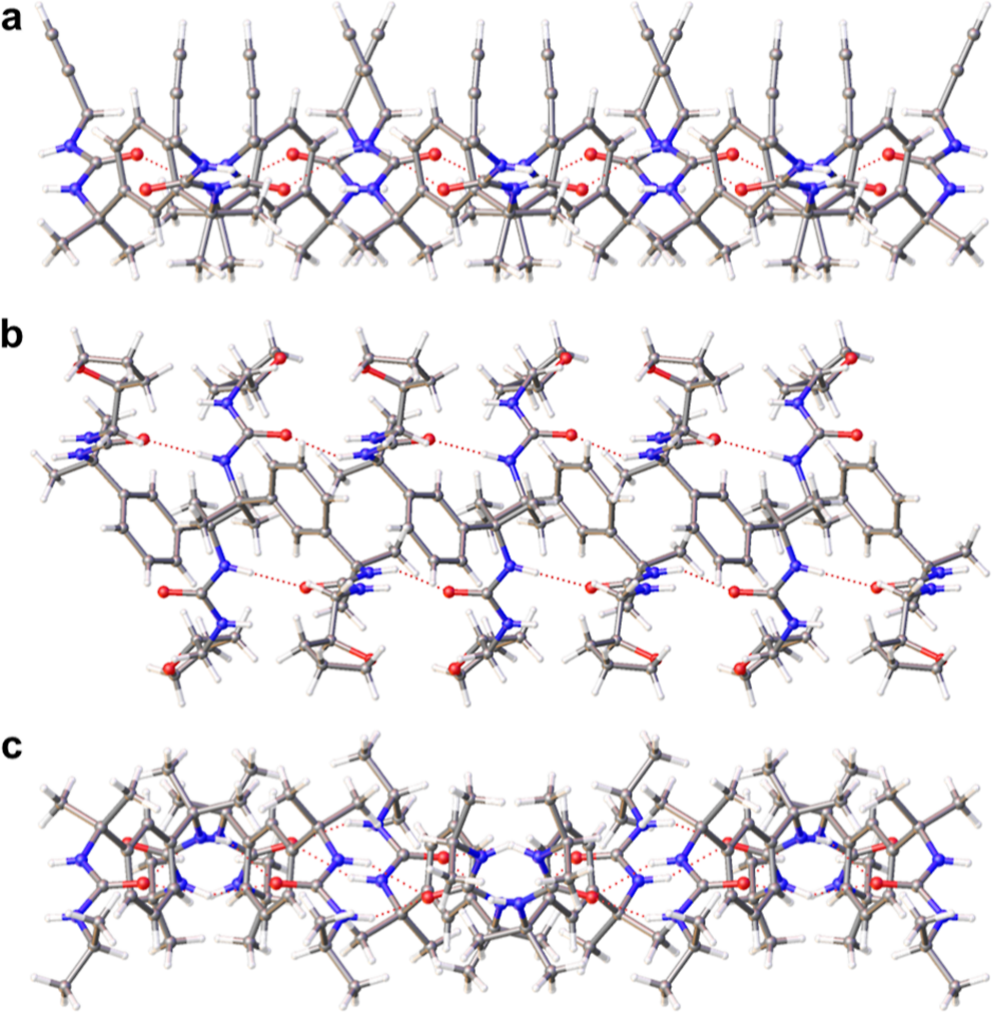
(a) Asymmetric lamella in the monoclinic structure of **4a** (Form 2), *A*_mol_ = 72.3 Å^2^; (b) thick symmetric lamella in the structure of *meso*-**6**, *A*_mol_ = 54.1 Å^2^; (c) thin symmetric lamella in the monoclinic structure of **2a** (Form 1), *A*_mol_ = 78.8 Å^2^.

In the lamellar structures of **1a**, **1b**, **1c**, **1d**, **2b**, **4** (Form 2), **5a** (Form 1), **5b** (Form 1), **5c**, **7a**, **7b** (Form 1), and **10b**, the end groups of the bis(urea) molecules are distributed asymmetrically, occupying a single face of the lamellar plane (Figures 2a and Supporting Information, S94). The remaining nine structures of **2a** (Form 1), **2c**, **4** (Form 1), *meso-***6**, **7b** (Form 2), **9a**, **9b**, **9c,** and **10a** display a symmetric arrangement of end groups (Figures 2b and Supporting Information, S95). The area spanned by each molecule (*A*_mol_ = 53–64 Å^2^) in a symmetric lamella is generally smaller than that in an asymmetric lamella (*A*_mol_ = 72–78 Å^2^). However, the end groups of the symmetric systems are accommodated on both faces of the lamella so are less closely packed. Indeed, the area per end group is 32–60% larger in the symmetric lamellae than in the asymmetric systems. Molecules with less bulky end groups tend to lie more parallel to the lamellar plane (Figure 2c). Thus, the symmetric lamellae of **2a** (Form 1) and **4** (Form 1) are comparable in thickness to an asymmetric lamella and display similarly large values of *A*_mol_ (78.8 and 70.3 Å^2^ respectively).

The weaker steric constraints of symmetric lamellae mean that they are particularly common among bis(urea)s with relatively bulky or inflexible end groups. For example, the inflexible *i*-propyl end groups of **2a** (Form 1) and relatively long *i*-pentyl end groups of **2c** favor symmetric assemblies, whereas the lamellae formed by the *i*-butyl analogue **2b** are asymmetric. Lamellae produced by the *n*-alkyl derivatives **1a**, **1b**, **1c,** and **1d** are all asymmetric, while those comprising benzyl derivatives, such as **9a**, **9b**, **9c,** and **10a**, are generally symmetric. Compound **10b** is a notable outlier, as its structure consists of asymmetric lamellae despite the presence of bulky bromo-substituted benzyl end groups. Conversely, propargyl derivative **4** forms symmetric lamellae in one of its polymorphs (Form 1) even though the alkyne end group is relatively small.

**Figure 3:**
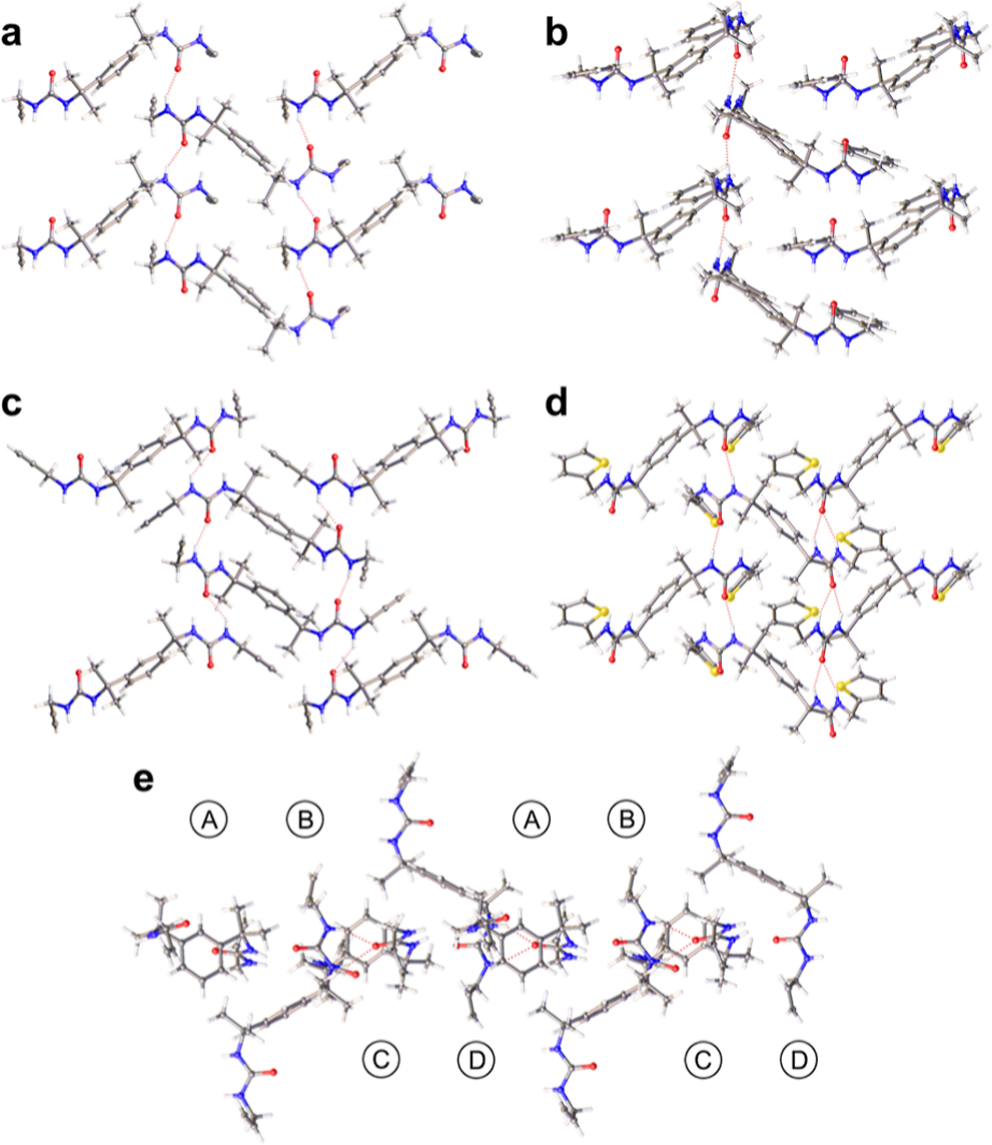
(a) [AB] network of antiparallel tapes in the asymmetric lamella of **4a** (Form 2); (b) [AB] network of antiparallel tapes in the symmetric lamella of **9a**; (c) [AABB] network of antiparallel tapes in the symmetric lamella of **4a** (Form 1); (d) [AB] network of syn-parallel tapes in the symmetric lamella of **7b** (Form 1); (e) [ABCD] network of **3**.

All of the asymmetric lamellae (Figure 3a) and most of the symmetric lamellae (Figure 3b) consist of antiparallel α-tapes with a relatively simple [AB] arrangement. Accommodating bulky end groups in a close-packed layered structure is more difficult, however, and symmetric lamellae may therefore exhibit more unusual hydrogen bonding networks. The structures of **2a** (Form 1) and **4** (Form 1) feature [AABB] repeat units (Figure 3c), as do the previously reported solvate structures of **11b** and **11c**.^48^ Other solvates of **11c** display an even more complex [AAAABBBB] structure, while the structures of **7b** (Form 2), **9b,** and **9c** consist of [AB] lamellae with syn-parallel arrangements of α-tapes (Figure 3d). The nonlamellar networks [ABCD] (Figures 3e and Supporting Information, S96) and [ABABCDCD] (Supporting Information, Figure 97) are also associated with sterically demanding end groups, such as the rigid allyl group of **3** and the bulky, electron dense halogen substituents of **5a**, **5b,** and **10c**.

The presence of α-tapes in a crystal constrains the symmetry and dimensions of the unit cell. In all of the bis(urea) structures, the α-tapes are aligned with a single cell axis. The corresponding cell parameter must therefore be a multiple of the urea–urea repeat distance, which lies in the range 4.4–4.7 Å: there are 18 structures with a cell axis of 9.0–9.4 Å, seven with an axis of 17.9–18.3 Å, and one with an axis of 34.9 Å. The structures in the CSD display similar constraints where tape motifs are present, featuring an average urea–urea distance of 4.65 Å (Supporting Information, Figure S93). It is interesting to note, however, that bis(urea)s comprising the tetramethylxylylene spacer produce more complex α-tape networks than other reported bis(urea) systems. For example, 56% of the networks in the literature consist of simple one-dimensional arrays of molecules with the repeat unit [A]. The compounds in our study never adopt this arrangement, instead forming two- or three-dimensional networks with repeat units that have rarely been described elsewhere. Our bis(urea) library therefore provides a unique insight into the structural diversity of hydrogen bonding networks and an opportunity to explore the role played by lamellar assemblies in the formation of a fibrous gel.

### Conformational Analysis

A key factor influencing the stability of an α-tape network is the geometry of the spacer between the urea groups. The complexity of the networks in this study can be attributed to the steric bulk of the spacer methyl groups, which restricts the range of conformations that the spacer can adopt. It is apparent that networks with the same arrangement of α-tapes consist of bis(urea) molecules with similar conformations and orientations. Thus, particular geometries and packing modes must be favored if the molecules are to self-assemble into an asymmetric lamella, which we hypothesize is a necessary step in the formation of a scrolled gel fibril. To better understand how the bis(urea) structure affects the self-assembly outcome, the geometries of the spacers in the crystal structures must be measured and correlated to the symmetry and connectivity of the α-tape networks.

**Figure 4:**
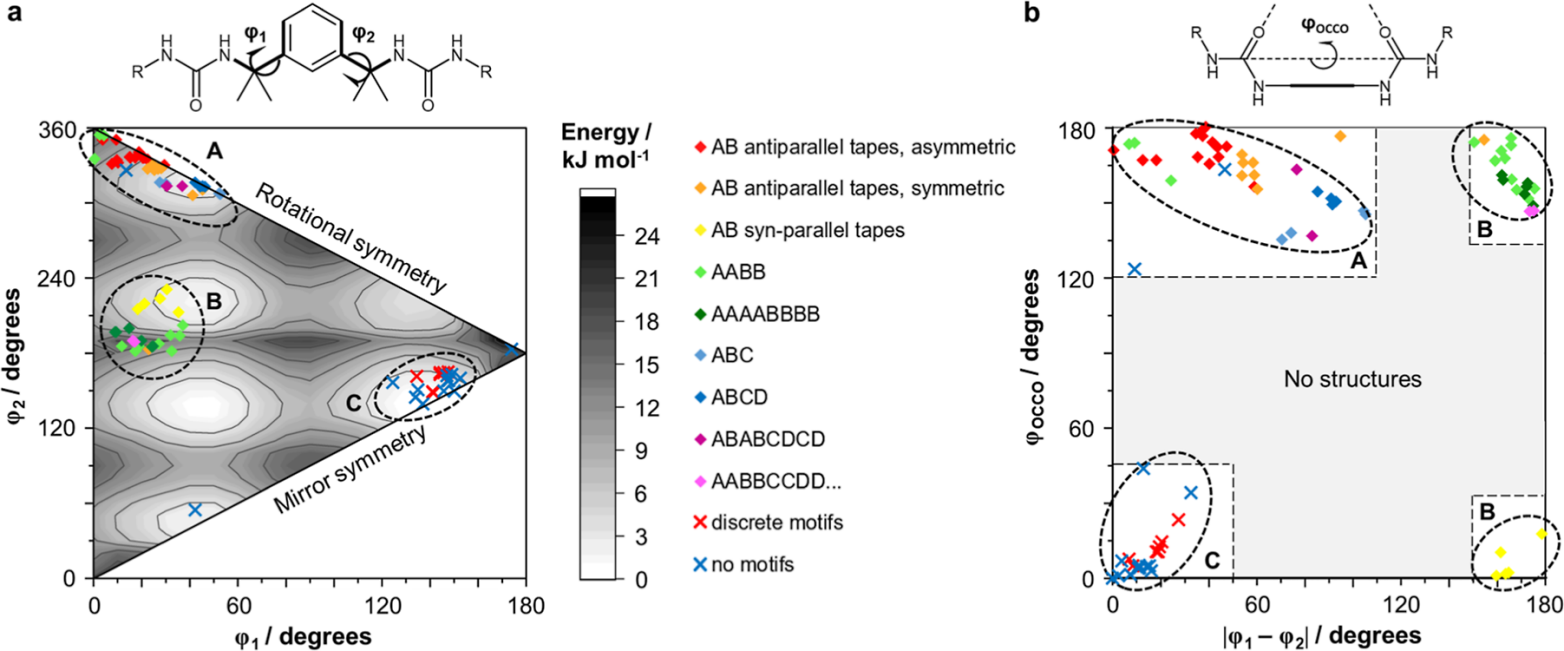
(a) DFT energy map of bis(urea) spacer conformations, specified by torsion angles of φ_1_ and φ_2_. Overlaid data points indicate the φ_1_ and φ_2_ values observed in the bis(urea) crystal structures. Data point colors and symbols indicate the existence of urea–urea motifs and, where applicable, the repeat unit of the α-tape network; (b) orientations of the urea groups in the crystal structures, specified by the urea–urea torsion angle φ_OCCO_ and the difference |φ_1_ – φ_2_|.

The conformation of the tetramethylxylylene spacer may be described in terms of φ_1_ and φ_2_, the two C–C–C–N torsion angles between the central aromatic ring and nearest alkyl-urea bonds (Figure 4a). Due to the structural symmetry of the spacer, all possible combinations of φ_1_ and φ_2_ lie in the range 0° ≤ φ_2_ ≤ 180°, within a triangle described by the lines φ_1_ = φ_2_ and φ_1_ = 360° – φ_2_. Conformations with mirror symmetry are situated on the line φ_1_ = φ_2_, while the line φ_1_ = 360° – φ_2_ corresponds to conformations that are rotationally (*C*_2_) symmetric. The conformational analysis may be extended by considering the relative orientations of the urea moieties (Figure 4b). Although the presence of multiple flexible bonds between the two C=O bonds limits the utility of the O–C–C–O torsion angle, φ_OCCO_, the sterically hindered spacer is sufficiently rigid for this parameter to offer a meaningful indication of the urea conformations.

To identify the most stable conformations of the bis(urea) spacer, energies were computed for a molecule of compound **1a** with varying combinations of φ_1_ and φ_2_. The calculations were performed in Gaussian 09 using DFT, with the B3LYP functional^59^ and cc-PVDZ basis set.^60^ The chosen basis set yields energy values similar to those obtained using the Pople basis set 6-31+G*,^61^ but was found to produce aromatic ring conformations closer to the expected planar geometry. For each 10° increment in φ_2_ between 0 and 180°, φ_1_ was increased from 0 to 360° in steps of 10°, and geometry optimization was performed on the remainder of the molecule. The final energy landscape was constructed by comparing the results for equivalent combinations of φ_1_ and φ_2_ and retaining those that were the lowest in energy. The calculations reveal a difference of 26.6 kJ mol^–1^ between the least and most stable conformations, with a standard deviation of 4.4 kJ mol^–1^. Conformations with φ_1_ and/or φ_2_ close to a multiple of 90° are most strongly disfavored. Interestingly, DFT modeling of a bis(oxazolidone), derived from **5a** as an intermediate for macrocycle formation, produced a similar energy landscape, suggesting that the groups attached to the methylated *m*-xylylene spacer only weakly affect its conformational preference.^48^

The importance of the spacer conformation on the stability of a bis(urea) crystal may be deduced by comparing the calculated minimum-energy values of φ_1_ and φ_2_ with the angles measured in crystal structures. For this analysis, it is useful to include the bis(urea) structures investigated in our previous work,^18,48^ in addition to the structures of other analogues reported in the literature (Supporting Information, Figure S98).^32,43−46,62,63^ A plot of the data (Figure 4a) displays three well-defined clusters corresponding to groups of crystal structures with differing characteristics. Cluster A includes most of the structures with α-tape networks and particularly those with asymmetric [AB] repeat units. Both torsion angles lie within 60 of 0°, and the spacer is either rotationally symmetric or nearly so. Cluster B encompasses the remaining tape-containing systems, which typically feature syn-parallel α-tapes or unusual lamellar repeat units. One torsion angle lies within the range spanned by Cluster A, while the other is close to 180°. Finally, cluster C comprises all of the structures that do not feature α-tapes and is characterized by two torsion angles within 60 of 180°. The existence of well-defined clusters in the conformational landscape illustrates the inflexibility of the tetramethylxylylene spacer and may be responsible for the strong tendency of the bis(urea)s to form crystalline materials. Structures with the same α-tape network display similar conformations, so a particular molecular arrangement may be targeted by including end groups that favor the corresponding values of φ_1_ and φ_2_.

Consistent trends are also visible in the relative orientations of the urea carbonyl groups (Figure 4b). As expected, all structures in cluster A and most structures in cluster B display φ_OCCO_ values between 135 and 180° due to the antiparallel arrangement of neighboring α-tapes. By contrast, the φ_OCCO_ values for structures in cluster C are typically below 60°. For structures without α-tapes, the difference between the end-group torsion angles, |φ_1_ – φ_2_|, is always less than 60°. Tape-containing structures display a wider range of |φ_1_ – φ_2_| values, but angles below 60° are still much more common. Combinations of torsion angles giving 60° < |φ_1_ – φ_2_| < 105° usually correspond to nonlamellar α-tape networks, while structures with |φ_1_ – φ_2_| > 150° are almost always located in cluster B. The latter group displays either antiparallel α-tapes in [AABB] and [AAAABBBB] networks or syn-parallel α-tapes with an [AB] repeat unit.

The DFT analysis predicts some major features of the conformational landscape, such as a lack of structures in the ranges 60° < φ_2_ < 120°, 60° < φ_1_ < 120°, and 240° < φ_1_ < 300°. The structures in cluster C are particularly close to a minimum in the energy landscape. However, it is clear that the calculations do not fully capture the factors influencing the crystallization outcome. For example, many data points in cluster B and on the edges of cluster A are situated near local maxima in the calculated energy landscape. There are also no examples of tape-containing structures with mirror-symmetric spacers, despite an abundance of minima on the line φ_1_ = φ_2_. These observations indicate that it is not always reasonable to base crystal structure predictions for flexible bis(urea)s on geometry optimizations in the gas phase. Although the lowest energy conformations are generally favored, higher energy geometries may be tolerated if they give rise to a hydrogen bonding network or a packing mode that is more compatible with crystal formation.

### Gelation Behavior

The gelation capacities of the bis(urea) compounds prepared in this study, along with three compounds (**11a**–**c**) reported previously,^18^ were tested by cooling hot 1% (w/v) solutions in a range of solvents (Table 1). Preliminary trials revealed that the solvents amenable to gel formation can be organized into three distinct classes according to polarity, with the solvents in each class producing similar aggregation outcomes. The least polar solvents, toluene and xylenes, are gelled by a number of analogues with extended alkyl and benzylic end groups. Many of these gelators are also able to gel di- and trichlorobenzenes, although the critical gelation concentrations (CGCs) of these systems tend to be slightly higher. Finally, a small number of compounds form gels in nitrobenzene, a significantly more polar solvent. There are 20 analogues that form gels in one or more of the solvent classes and seven that appear to be completely nongelating. Most of the gelators exhibit CGCs in the range 0.5–1.0% (w/v). For example, toluene solutions of **9c**, 1,2-dichlorobenzene solutions of **6a,** and nitrobenzene solutions of **1a** and **4** undergo complete gelation if their concentrations exceed 0.5, 0.9, 0.7, and 0.9% (w/v), respectively. At lower concentrations, only a small number of compounds form sample-spanning gels. The alkyl derivatives **1d**-**f** produced weak gels at concentrations of just 0.05% (w/v).

**Table 1:** Results of Gelation Experiments for Bis(urea) Derivatives in 1,2-Dichlorobenzene, Nitrobenzene, Nitromethane, Acetonitrile, and Toluene^a^

	1,2-PhCl_2_	PhNO_2_	MeNO_2_	MeCN	PhMe
**1a**	GP	G^T^	P	P	I
**1b**	PG^C^	GP	μX^N^	μX^N^	I
**1c**	PG^C^	GP	P	P	I
**1d**	G^C^	GP	GP	P	G^C^
**1e**	G^C^	GP	GP	GP	G^C^
**1f**	G^C^	GP	GP	GP	G^C^
**2a**	P	P	X^P/N^	P	I
**2b**	P	P	P	P	G^T^
**2c**	G^T^	P	μX^N^	μX^N^	G^T^
**3**	P	P	μX^B^	X^B^	I
**4**	P	G^C^	P	P	P
**5a**	P	P	P	X^B^	I
**5b**	P	P	P	X^B^	I
**5c**	G^T^	PG^T^	PG^T^	X^B^	PG^T^
**6**	G^C^	PG^C^*	P	P	G^c^
**7a**	G^T^	P	P	P	P
**7b**	GP	G^T^	μX + X^P/R^	X^P/R^	G^O^
**8**	G^T^	G^T^	X^B^	X^B^	P
**9a**	P	P	X^P^	X^P/N^	P
**9b**	G^T^	PG^T^	P	P	G^O^
**9c**	G^C^	P	P	P	G^O^
**10a**	G^T^	P	P	X^p^	PG^O^
**10b**	G^T^	P	P	μX^B^	G^O^
**10c**	PG^T^	G^T^	μX^R^	μX^R^	P
**11a**	P	X^P^	X^N^	μX^N^	I
**11b**	P	μX + X^N^	X^P/N^	X^P^	I
**11c**	P	G^T^ + X^R^	GP + X^B^	X^B^	I

a Observations were made after heating 1% (w/v) solutions in 2 cm^3^ sealed vials and allowing the materials to cool to room temperature for 1 h. Results are marked with a letter corresponding to the aggregation outcome: G = gel (highlighted in bold), PG = partial gel, X = crystal, μX = microcrystals, GP = gelatinous precipitate, and *P* = precipitate. Superscripts are used to denote the appearance of gels and crystals: for gels, C = clear (transparent), T = translucent, and O = opaque, while for crystals, B = blocks, N = needles, P = plates, and R = rods. An asterisk is used to denote materials prepared from an 8% (w/v) solution, in order to exceed the solubility limit of the bis(urea).

The gelation trials reveal that even small variations in the end group structure can lead to dramatic differences in the aggregation behavior. Notably, the methyl derivative **1a** forms gels only in nitrobenzene, but analogues with longer alkyl end groups gel the relatively nonpolar solvents toluene and 1,2-dichlorobenzene. Likewise, the highest gelation capacities of 3-chlorobenzyl analogue **10a** are observed in 1,2-dichlorobenzene, while 4-chlorobenzyl analogue **10c** behaves as a gelator primarily in nitrobenzene. A clear trend is that compounds with more flexible and extended end groups are more likely to display gelation behavior. For example, compounds **1b**, **1c,** and **9a** are nongelators, but analogues **1d**-**f**, **9b,** and **9c** feature longer alkyl chains and are able to form gels in a range of solvents.

**Figure 5:**
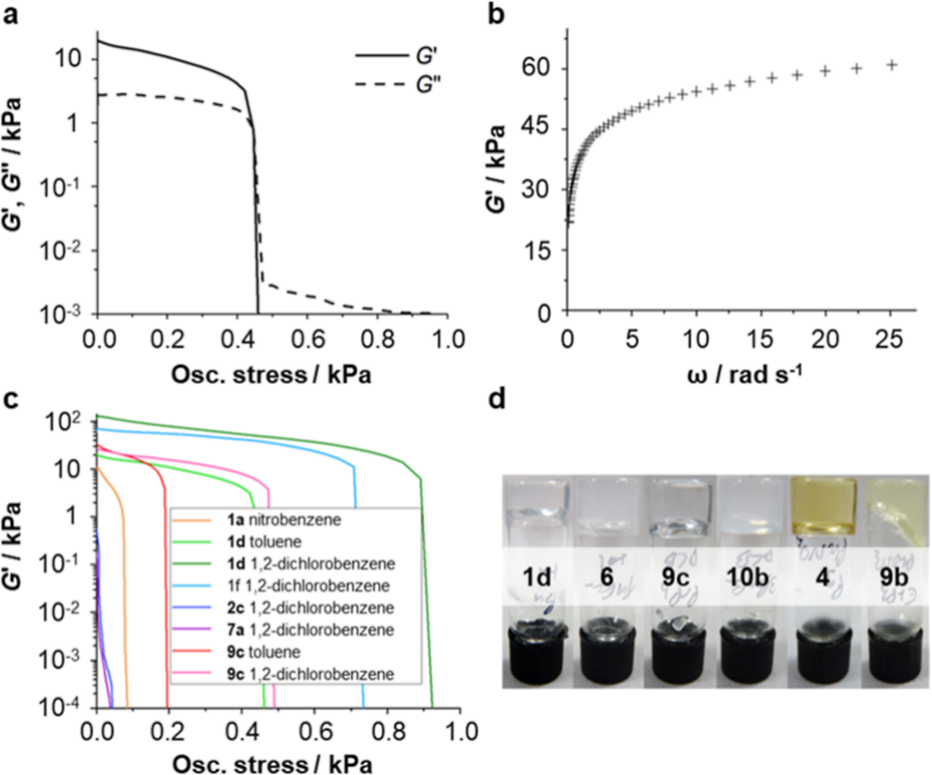
(a) Variation of *G*′ and *G*″ with shear stress for a gel of **1d** in toluene; (b) variation of *G*′ with shear frequency for a gel of **1d** in toluene; (c) variation of *G*′ with oscillatory stress for gels comprising different solvents and bis(urea) gelators; (d) typical gels of (left to right) **1d** in toluene, **6** in toluene, **9c** in 1,2-dichlorobenzene, **10b** in 1,2-dichlorobenzene, **4** in nitrobenzene, and **9b** in nitrobenzene. All gels were prepared from 1% (w/v) solutions and analyzed at 10 °C. A constant frequency of 1 Hz was used in the stress sweep experiments, and a constant stress of 1 Pa was used in the frequency profile.

The rheological properties of the gels were analyzed by means of oscillatory shear experiments. In all of the gels studied, *G*′ is an order of magnitude larger than *G*″ at low stresses, and collapse of the material occurs above a well-defined yield stress (Figures 5a and Supporting Information, S99). Moreover, there is a gradual rise in *G*′ with increasing frequency ω, as expected for fibrous gels (Figures 5b and Supporting Information, S100). The properties of the gels may be tuned by altering either the structure of the gelator or the solvent being gelated (Figure 5c). For example, compound **1d** produces stronger gels at 1% (w/v) in 1,2-dichlorobenzene than in toluene, with plateau *G*′ values of 130 and 21 kPa and yield stresses of 850–900 and 420–450 Pa, respectively. Gels of **2c** and **7a** in this solvent, however, are significantly less robust: the *G*′ values are just 570 and 170 Pa, and the yield stresses are less than 30 Pa. Nitrobenzene gels also tend to be relatively weak. Compound **1a** forms a nitrobenzene gel with a plateau *G*′ of 11 kPa and yield stress of 70–75 Pa, and nitrobenzene gels of the other derivatives were found to collapse too readily to be reliably characterized (Figure 5d).

**Figure 6:**
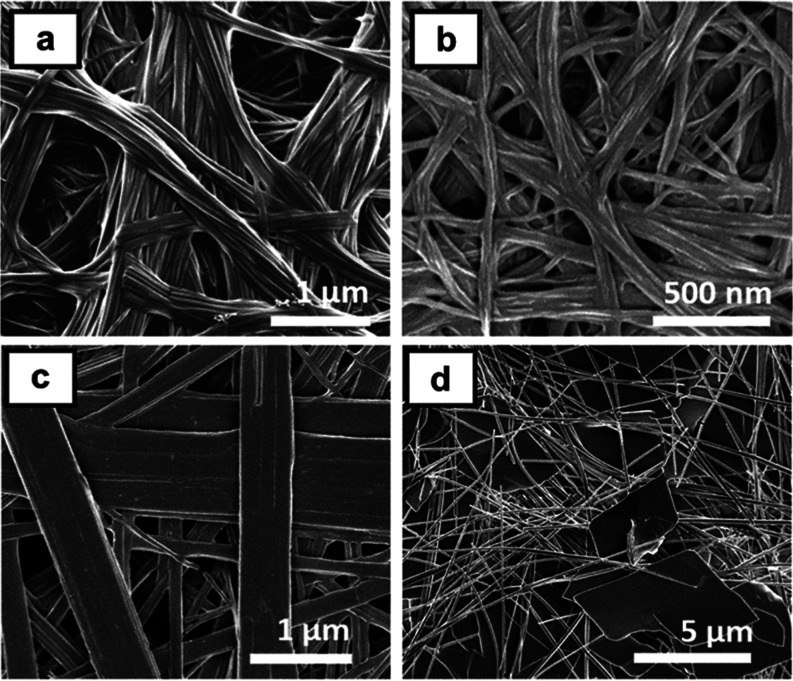
SEM micrographs of xerogels of (a) **1d** and (b) **2b** in toluene and precipitates of (c) **1e** from nitrobenzene and (d) **2a** from 1,2-dichlorobenzene.

To gain insight into the structural differences underlying the variation in rheological properties, xerogels were prepared from a selection of 1% (w/v) gels and analyzed by scanning electron microscopy (SEM). The micrographs reveal that the microstructures of gels in toluene and 1,2-dichlorobenzene are remarkably similar: in all cases, the materials consist of uniform, unbranched fibrils 20–30 nm in diameter (Figures 6a,b and Supporting Information, S101). This result suggests that the strengths of the gels are determined by the density and connectivity of the fiber network over larger length-scales. However, it is also possible that structural changes during drying of the gels renders the images unrepresentative of the wet materials.^64^ Indeed, SEM micrographs show that xerogels prepared from gels of **7b** and **8** in nitrobenzene consist entirely of microcrystalline particles (Supporting Information, Figure S102), similar to those of nongelatinous precipitates (Supporting Information, Figure S103), indicating that preparation of the sample leads to quantitative recrystallization of the gel assemblies. Interestingly, microcrystals in the precipitates of **1e** and **2a**, from nitrobenzene and 1,2-dichlorobenzene respectively, are more fibrous in nature but do not give rise to sample-spanning gels (Figure 6c,d). It is possible that these aggregates are too short and rigid to generate an extended interconnected network capable of percolating the system and immobilizing the solvent.

There are clear correlations between the gelation properties of the bis(urea)s and the structures of their α-tape networks. The nonlamellar repeat unit [ABCD] is observed in the crystal structures of **2a**, **3**, **5a**, **5b,** and **9a**, and none of these compounds display any gelation capacity. It may be deduced that a bis(urea) is likely to be nongelating if it displays a nonlamellar α-tape network in at least one of its crystal forms. Conversely, compounds **1d**, **5c,** and **7b** produce asymmetric lamellar networks and deliver stable gels in the widest range of solvents, supporting our hypothesis that asymmetric lamellae can develop more readily into gel fibrils. The main exceptions to these trends are **10c**, which acts as a gelator despite forming a nonlamellar [ABABCDCD] network, and **11a** and **11b**, which form several lamellar crystal structures with π–π stacking motifs and solvent–picolyl interactions between the neighboring layers.^18^ We conclude that crystal structures offer a useful guide for predicting the gelation behavior of a bis(urea) but may be of limited use in systems where the α-tape network is particularly complex or the end groups can be incorporated into additional supramolecular motifs.

### Scrolling Pathways

MD simulations of bis(urea) lamellae have been shown to be helpful for identifying the underlying causes of gel formation.^18^ In particular, such simulations highlight the possibility of scrolling behavior, wherein lamellae in solution fold into stable unbranched fibrils. To assess how the bis(urea) structure and packing influence this folding pathway, a selection of the lamellar assemblies in this study were simulated using the atomic coordinates of the single-crystal structures. The model lamellae consisted of 600 molecules and were simulated under vacuum at a temperature of 300 K, controlled via a Berendsen thermostat. Simulations were conducted over 500 ps, as this short time period was found to be sufficient to detect the incidence and direction of scrolling and capture all major morphological changes involved.

**Figure 7:**
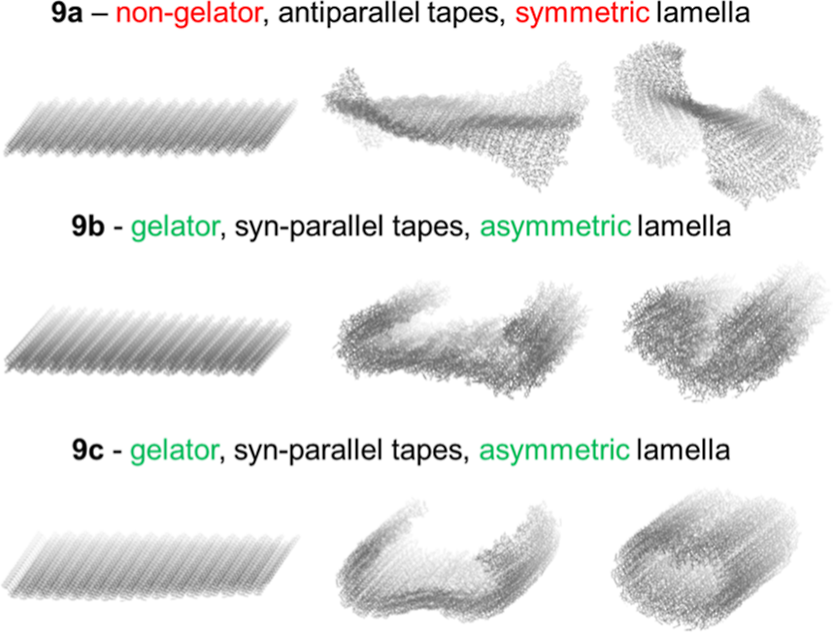
Selected frames from 500 ps MD simulations of lamellae extracted from the crystal structures of **9a**, **9b,** and **9c**. Scrolling occurs in the asymmetric lamellae of the gelators **9b** and **9c** but not in the symmetric lamella of nongelator **9a**.

The simulation results reveal that many lamellae adopt similar scrolled morphologies despite substantial differences in the bis(urea) end groups (Figures 7 and Supporting Information, S104–S106). Indeed, scrolling occurs whenever the end groups are distributed asymmetrically between the two faces of the lamella. By contrast, symmetric lamellar assemblies of **2c**, *meso*-**6**, **9a,** and **10a** undergo relatively little deformation within the simulation time scale (Supporting Information, Figure S107). Scrolling lowers the energy of a lamella by 26–34 kJ mol^–1^, greatly exceeding the stabilization produced by nonscrolling deformation pathways (Supporting Information, Figure S108). The energy of the system decreases even before the scrolling lamella makes contact with itself as the increase in curvature allows the groups on each side of the lamella to adopt a more stable packing arrangement. The product of scrolling is an unbranched fibril, which is consistent with the gel micromorphology observed in the SEM images.

**Figure 8:**
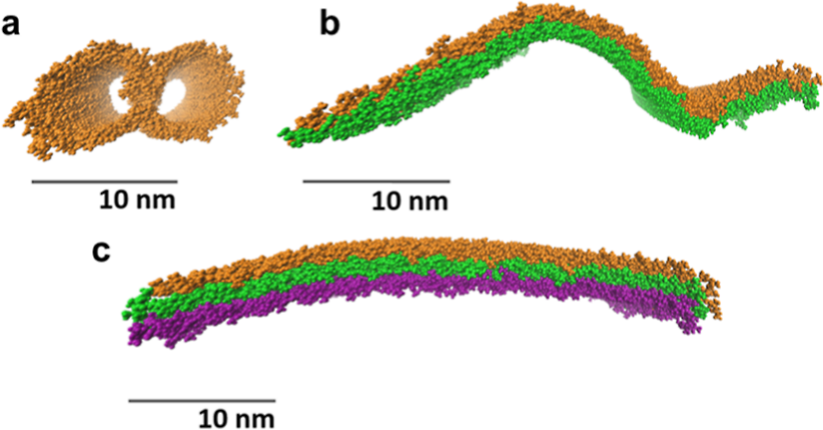
Effect of increasing lamellar thickness on the maximum attainable radius of curvature: (a) monolayers derived from the structure of **1a** undergo scrolling, but polar stacks of (b) two and (c) three lamellae bend relatively little due to their large bending moduli. All images represent the final frames of 500 ps MD simulations under vacuum at 300 K.

We have previously proposed that the process may continue until the radius of the fibril exceeds the natural radius of curvature of the scrolling lamella.^18^ Additional layers will be sufficiently strained to detach from the fibril and form a separate structure. The threshold radius of curvature may increase if stacks of lamellae are formed before scrolling takes place, since the bending modulus of the structure increases as the cube of its thickness. Furthermore, stacking of lamellae in a nonpolar fashion may eliminate the asymmetry of the system, removing the driving force for lamellar scrolling. Our simulations indicate that single lamellae scroll with a radius of 3–10 nm, depending on the steric bulk of the bis(urea) end group (Figure 8a). However, the radius increases to 10–20 nm in a two-layer system (Figure 8b) and over 70 nm if a third lamella is deposited (Figure 8c). The results provide a robust explanation for the structural uniformity of the gel fibrils, which grow to a consistent maximum diameter of 20–30 nm.

The MD simulations correctly indicate that compounds **1a**, **1d**, **2b**, **4**, **5c**, **7a, 7b**, **10b,** and **11c**, which feature asymmetric lamellae in their crystal structures, should be highly compatible with gel formation. For compounds that form symmetric lamellae, such as **2c**, **6**, **10a,** and **10c**, gelation may be associated with asymmetric assemblies that have not been identified in the crystal phase. It is possible, for example, that gels of **10a** and **10c** form from asymmetric lamellae similar to those of bromo analogue **10b**. An alternative explanation is that the opposing faces of the symmetric lamellae feature bis(urea) end groups with different conformations, creating sufficient asymmetry for scrolling to occur. Conformational asymmetry in otherwise symmetric lamellae can be observed in the syn-parallel α-tape networks of **7b** (Form 2), **9b**, **9c,** and **11b**. Simulations of these lamellae reveal that scrolling is feasible, but only while the surface groups remain in a nonequilibrium state.^18^ Consequently, the bis(urea) end groups must be sufficiently flexible for the conformational asymmetry to persist throughout the scrolling process (Figures 7 and Supporting Information, S106). This mechanism could explain why extended alkylbenzene compounds **9b** and **9c** are both effective gelators, while shorter benzyl analogue **9a** forms crystals or microcrystalline precipitates in all of the tested solvents.

**Figure 9:**
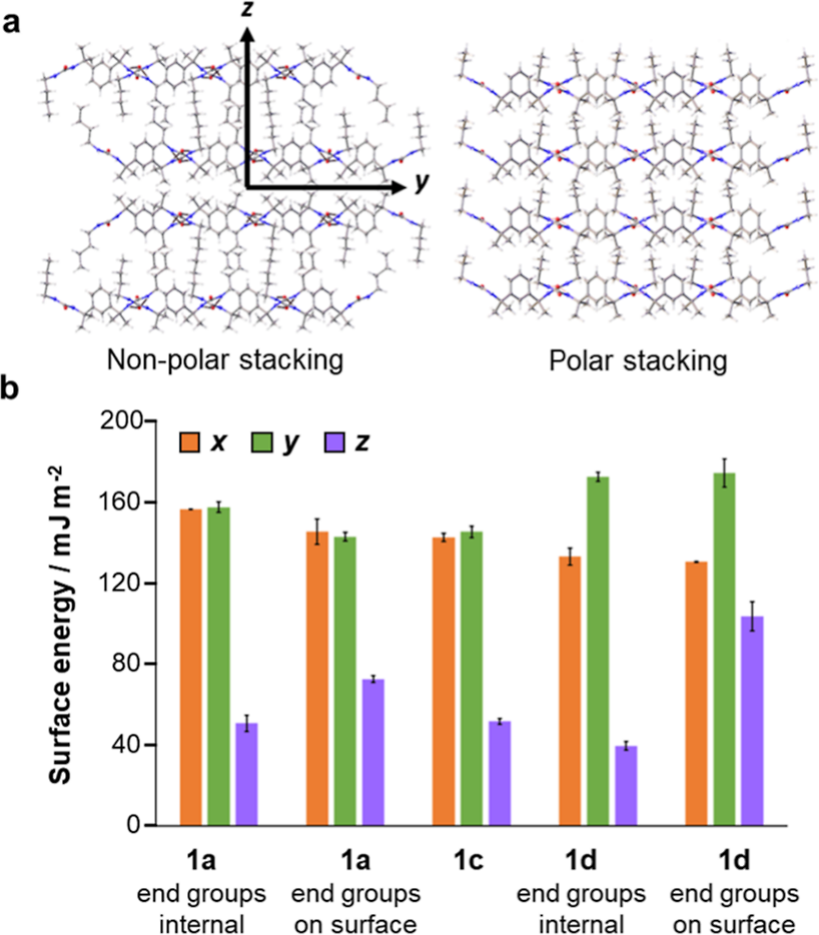
(a) Nonpolar lamellar stacking in structure of **1d** and polar stacking in structure of **1c**. The axes *y* and *z* correspond to the tape axis and normal axis of the lamellae, respectively; (b) estimates of surface energy derived from MD simulations under constant-*NVT* conditions in a vacuum.

The gelation capacities of alkyl derivatives **1a**-**1f** also vary with the length of the end group. The compounds typically form gels in toluene and dichlorobenzenes and become more effective gelators as the chain length increases. However, **1a** forms gels only in nitrobenzene, while **1b** and **1c** are nongelating. The behavior of the ethyl and propyl derivatives is anomalous, given that these compounds, like **1a** and **1d**, self-assemble into asymmetric lamellae that should be highly susceptible to scrolling. A key difference is that **1b** and **1c** produce polar crystals, wherein lamellae are stacked in the same orientation, whereas lamellae of **1a** and **1d** stack in a nonpolar fashion (Figures 9a and Supporting Information, S109). Crystallization of a polar material may occur more readily, in preference to fibril formation, since scrolling generates a similarly polar layered structure that provides a more suitable nucleation site for crystal growth. A drawback of this hypothesis is that it is inconsistent with the behavior of compounds **2b**, **4,** and **10b**, which also form polar stacks of asymmetric lamellae but are nonetheless effective gelators in a range of solvents.

It is also possible that crystals of **1b** and **1c** interact more strongly than do those of **1a** and **1d**, favoring the formation of a crystalline stack. To test this possibility, the surface energies of the structures of **1a**, **1c,** and **1d** were estimated via MD simulations, according to a method outlined in our previous study.^18^ The stability of each crystal face was evaluated by simulating crystallites with varying numbers of layers along the corresponding cell axis. Four crystallites were simulated for each face, and the process was repeated for the nonpolar crystals of **1a** and **1d** to determine the relative stabilities of the two lamellar surfaces. The results reveal that the surface energies of the crystals are highest along axes parallel to the lamellar plane, confirming that propagation of the α-tape network is more favorable than lamellar stacking (Figure 9b). Furthermore, lamellae of **1c** do not interact significantly more strongly than do those of **1a** and **1d**. Indeed, lamellae bind together most strongly if they adopt a nonpolar arrangement, such that both of the interacting surfaces are functionalized with alkyl end groups. Based on these simulations, we cannot conclude that polar stacks of lamellae self-assemble more readily or that the lamellar structures of **1b** and **1c** are less compatible with fibril formation.

Although all asymmetric lamellae are susceptible to scrolling, the deformation pathway depends on the arrangement of α-tapes and the structure of the bis(urea) end group. If the α-tapes are antiparallel, scrolling usually occurs around an axis perpendicular to the tape axis, and the end groups are located on the inner surface of the resulting fibril (Supporting Information, Figure S110).^18^ Thus, the simulations indicate that the bis(urea) end groups do not, in many cases, alter the surface chemistry of the gel fibers. A different scrolling mechanism is observed in the lamellae of **1a** and **1b** and the syn-parallel networks of **7b** (Form 2), **9b**, **9c,** and **11b**. In these systems, the scrolling axis is parallel to the α-tapes, and the end groups are presented to the outside of the scrolled structure. Intriguingly, this pattern of scrolling could also be induced in a lamella of **1d** by randomly replacing just 25% of the molecules with molecules of **1a** (Supporting Information, Figure S111). It is unclear whether such mixed assemblies can be generated in practice or even if the simulations of scrolling in vacuo replicate the true structures of the gel fibrils. Indeed, preliminary attempts to expand the gelation capacity of **1d**, by preparing a 1.0% (w/v) solution of the compound with a nongelating concentration of **1a** (0.2% (w/v)) in nitrobenzene, were unsuccessful. Further work will be required to test the sensitivity of the scrolling simulations to the gelation conditions, including gelator–solvent interactions, and the correlation between simulated and experimental aggregation outcomes.

**Figure 10:**
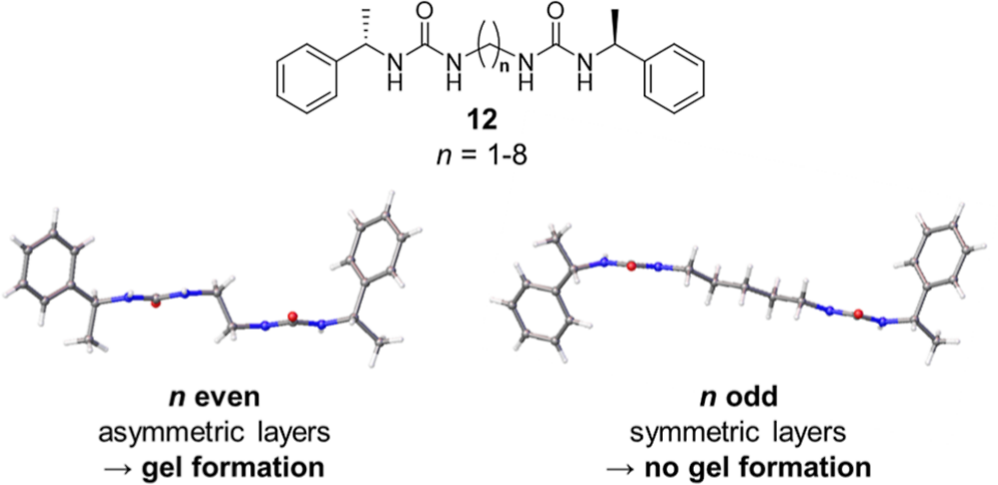
Application of the scrolled sheet model to previously reported compounds of type **12**.^27,67^.

Scrolling is a highly general phenomenon, giving rise to the fibrous morphologies of minerals and organic polymers in addition to small-molecule gels.^65^ The lamellar scrolling model is consistent with small-angle neutron scattering (SANS) studies of **1d** gels, which indicate that the developing fibrils form entangled bundles with radii of gyration in the range 20–75 nm.^47^ The radii of curvature observed in our MD simulations are also comparable to the dimensions of other tubular hydrogen bonded networks, such as the urea-based fibrils reported by Mirzamani et al.,^66^ suggesting that these structures may form via an analogous self-assembly pathway. The presence of asymmetric lamellae in a crystal structure could therefore serve as a useful predictor of the gelation capacity. For example, it has previously been shown that molecules of type **12** are gelators only if the alkylene spacer contains an even number of CH_2_ groups (Figure 10).^27,67^ Crystal structures of these compounds consist of well-defined layers, featuring an asymmetric arrangement of phenyl end groups when the number of CH_2_ groups is even and a symmetric arrangement when the number is odd. Gelation could occur when similar asymmetric lamellae form in solution, developing rapidly into fibrils through spontaneous scrolling. The symmetric lamellae of the nongelator analogues would be insusceptible to scrolling and thus more likely to assemble into multilayer stacks, favoring the growth of a crystalline material.

## Conclusions

There have been many attempts to understand the formation of supramolecular gels based on the crystal structures of the molecules involved. An analysis of 26 bis(urea)s with a shared spacer moiety reveals that the most effective gelators self-assemble into lamellar hydrogen bonding networks with asymmetric surface structures. Conversely, a compound is highly unlikely to act as a gelator if it displays a nonlamellar network of hydrogen bonds in at least one of its crystal forms. Asymmetric lamellae feature hydrogen bonding networks with similar repeat units and usually consist of molecules with flexible end groups that can more easily adopt a densely packed arrangement. MD simulations of these lamellae suggest that gel fibrils form through spontaneous scrolling, producing a narrow, unbranched fibril, consistent with SEM images of the gel morphology. Scrolling can occur only in lamellae that have not undergone multilayer stacking, so the balance between gelation and crystallization may be highly dependent on the choice of solvent and aggregation conditions. Nonetheless, the likelihood of gelation may be increased by choosing end groups that are compatible with an asymmetric lamellar structure and avoiding factors that inhibit α-tape formation, such as substituents capable of forming competing supramolecular motifs. These insights could facilitate the design of more effective gelators and offer guidance for controlling the outcomes of other self-assembly processes.

## Experimental Section

### Synthesis

Compounds **1**-**10** were synthesized by the addition of 1,3-bis(1-isocyanato-1-methylethyl)benzene (0.1 cm^3^, 0.43 mmol) to a stirred solution of the necessary amine (0.97 mmol) in chloroform (20 cm^3^) under air at 20 °C. In the synthesis of **5b**, the amine was introduced as a hydrochloride salt with triethylamine (2.1 equiv) to aid dissolution. The reaction mixture was left to stand for 24 h at 20 °C and then concentrated in vacuo and filtered under suction. The collected solids were washed with chloroform (2 × 20 cm^3^) and dried in a drying pistol. Details for each compound are given in the Supporting Information.

### X-ray Crystallography

Crystals were obtained by the slow, partial evaporation of 1% (w/v) solutions under ambient conditions. Crystals of **2b** were obtained from ethanol and polymorphs of **4**, **5b,** and **7b** from ethanol, acetonitrile, and 1-propanol, respectively. Methanol was used as the solvent for all of the other crystallizations. Details of individual data collections are given in the Supporting Information. The structures were solved by direct methods and refined by full-matrix least-squares on F2 for all data using the SHELX suite of programs^68^ in Olex2.^69^ All non-hydrogen atoms were refined in anisotropic approximation, and hydrogen atoms were mainly placed in the calculated positions and refined in riding mode. Crystallographic data for the structures have been deposited with the Cambridge Crystallographic Data Centre as supplementary publications CCDC-2297952–2297973 (**1c**, **5b**F2, **2c**, **2b**, **10a**, **7b**F1, **6**, **7a**, **5c**, **9b**, **3**, **10c**, **7b**F2, **10b**, **2a**F1, **9c**, **9a**, **4**F1, **1b**, **1a**, **2a**F2, **4**F2), 2310612 (**5b**F1).

Additional experimental details, including instrumental and computational information, are given in the Supporting Information.

## Data Availability

Underlying data for this work is available at https://pubs.acs.org/doi/10.15128/r2js956f85j.

## Supporting Information Available

Synthesis of compounds **1**–**10**, NMR and mass spectra, elemental analysis data, gel characterization, MD simulation images, CSD survey results, and instrumental methods (PDF)
Crystallographic data have been deposited with the Cambridge Crystallographic Data Centre CCDC 2297952–2297973, 2310612
