# Supplementary material for: Scrolling in Supramolecular Gels: A Designer’s Guide
Source: Chem Mater. 2024 Mar 9;36(6):2799–809. doi: 10.1021/acs.chemmater.3c03013 (PMC10976645; doi:10.1021/acs.chemmater.3c03013)
Supplement: Supplementary file 1 — cm3c03013_si_001.pdf [file cm3c03013_si_001.pdf]

Supporting Information for
Scrolling in Supramolecular Gels: A Designer's Guide

*Christopher D. Jones,[a] Laurence J. Kershaw Cook,[b] Anna G. Slater,[b] Dmitry S. Yufit[a] and Jonathan W. Steed[a]**

a) Department of Chemistry, Durham University, Durham DH1 3LE, UK.

b) Department of Chemistry and Materials Innovation Factory, University of Liverpool, Crown Street, Liverpool L69 7ZD, UK.

Contents

1. Experimental

1.1	Compositional analysis	2
1.2	Single-crystal X-ray diffraction	2
1.3	Rheometry	3
1.4	Scanning electron microscopy	3
1.5	NMR spectroscopy	3
1.6	Computational work	3
1.7	Synthesis	5

2. Results

2.1	Characterization	13
2.2	CSD survey	58
2.3	Crystal structure analysis	70
2.4	Gel characterization	74
2.5	Molecular dynamics	78

3. References 81

1. Experimental

1.1 Compositional analysis

Elemental analysis and mass spectrometry

Electrospray-ionization mass spectra of **5b** and **5c** were obtained from 0.001% (w/v) solutions of the compounds in methanol, doped with 0.1% (v/v) formic acid. The samples were analyzed using a Waters Acquity UPLC-MS (H-class) instrument. All other mass spectra were obtained from dilute (1 mg ml^{-1}) samples in methanol using a TQD mass spectrometer (Waters Ltd.).

Elemental analysis of **5b** and **5c** was performed using a Thermo FlashSmart Elemental Analyzer. All other materials were analyzed using an Exeter CE-440 Elemental Analyzer.

1.2 Single-crystal X-ray diffraction

Crystals were obtained by slow, partial evaporation of 1% (w/v) solutions under ambient conditions. Crystals of **2b** were obtained from ethanol, and polymorphs of **4**, **5b** and **7b** from ethanol, acetonitrile and 1-propanol respectively. Methanol was used as the solvent for all other crystallizations.

Diffraction experiments

All data were collected at low temperatures in the range 100-150 K maintained by Cryostream (Oxford Cryosystems) open-flow nitrogen cryostats. Crystal structures of **5b** (Form 1) and **5c** were acquired using a Rigaku AFC12K goniometer employing mirror monochromated Mo Kα radiation (λ = 0.71073 Å) radiation from a Rigaku 007HF Molybdenum rotating anode microfocus X-ray target source. For compound **5c**, a Rigaku Saturn 724+ CCD area detector was employed to collect the diffraction images, whereas for **5b** (Form 1) an upgraded Rigaku Hypix-6000HE Hybrid Photon Counting detector was used. For both **5b** (Form 1) and **5c**, data reduction and processing were achieved using CrysAlisPro software package and empirical absorption corrections using spherical harmonics were implemented in the SCALE3 ABSPACK scaling algorithm.

The structure of **5b** (Form 2) was acquired using a Bruker D8 Venture diffractometer equipped with a photon 100 dual-CMOS chip detector and operating a Mo Kα (λ = 0.71073) microfocus X-ray source. Data collection and processing were conducted using Bruker APEX3 software. The structures were solved using either direct or dual-space methods and refined by full matrix least-squared on F2 with SHELXL2019.

Data for compounds **1a**, **1b**, **1c**, **2b**, **7b** (Form 2) and **9a** were collected on an Agilent XCalibur (Sapphire-3 CCD detector, fine-focus sealed tube, graphite monochromator) 4-circle diffractometer. Data for weakly diffracting compounds **2c** and **9c** were collected on a Rigaku Saturn 724+ diffractometer at station I19 of the Diamond Light Source synchrotron (undulator, λ = 0.6889 Å) and processed using Bruker APEXII software. All other crystal structures were acquired using a Bruker D8 Venture (Photon100 CMOS detector, IµS-microsource, focusing mirrors) 3-circle diffractometer with Mo Kα (λ =0.71073Å) or, for **10c**, Cu Kα λ = 1.54178 Å) radiation.

The structures were solved by direct methods and refined by full-matrix least squares on F2 for all data using the SHELX suite of programs[1] in Olex2.[2] All non-hydrogen atoms were refined in anisotropic approximation, hydrogen atoms were mainly placed in the calculated positions and refined in riding mode. Crystallographic data for the structures have been deposited with the Cambridge Crystallographic Data Centre as supplementary publication CCDC-2297952-2297973 (**1c**, **5b**F2, **2c**, **2b**, **10a**, **7b**F1, **6**, **7a**, **5c**, **9b**, **3**, **10c**, **7b**F2, **10b**, **2a**F1, **9c**, **9a**, **4**F1, **1b**, **1a**, **2a**F2, **4**F2), 2310612 (**5b**F1).

1.3 Rheometry

Oscillatory rheometry measurements were performed using a TA Instruments AR 2000 on a rough Peltier plate with a 25 mm rough plate geometry and 2.5 mm gap. Samples were prepared by boiling gelator solutions in sealed 7 cm^3 vials. The hot solutions were poured into a 25 mm cylindrical glass mold on the Peltier plate and the gels allowed to form over 30 minutes at 10-20 °C prior to analysis. The gels were equilibrated for 30 minutes at the experiment temperature before removing the mold and initiating the analysis. Frequency sweep experiments were performed with a constant applied stress of 1 Pa, and stress sweep experiments with a constant frequency of 1 Hz. Time sweep experiments were performed without removing the glass mold, and with a fixed stress and frequency of 1 Pa and 1 Hz respectively.

1.4 Scanning electron microscopy

SEM samples were prepared on silicon wafers, dried in air for 2 days, and coated with 2 nm of platinum using a Cressington 328 Ultra High Resolution EM Coating System. The images were obtained using an FEI Helios NanoLab DualBeam microscope in immersion mode, with beam settings of 1.5 kV and 0.17 nA.

1.5 NMR spectroscopy

Solution-state NMR spectra were recorded in DMSO-d_6 using a Bruker Avance 400 MHz spectrometer without an internal reference. The concentrations of samples were typically in the range 1-2% (w/v).

1.6 Computational work

Molecular dynamics

MD simulations were performed in GROMACS 4.6.2[3] using the General Amber Force Field (GAFF).[4] The Antechamber package[5] was used to assign bonded and non-bonded interaction parameters, and calculate atomic charges via the semi-empirical AM1 method with bond charge correction (AM1-BCC).[6] The initial structures were obtained from single-crystal data, bounded with a 50 nm cubic periodic box and subjected to an initial energy minimization step via a steepest-descent procedure. Production runs were performed in the absence of solvent using a constant-NVT ensemble, with random initial velocities assigned according to a Maxwell distribution at 300 K. The temperature was controlled via a Berendsen thermostat with a time constant of 0.1 ps[7]. Although it fails to generate a correct canonical ensemble, the Berendsen thermostat was chosen as it effects efficient convergence of both temperature and potential energy, allowing the

dynamics of many large assemblies to be compared under isothermal conditions over extended timescales.

Folding simulations were carried out for 1500 ps with a time step of 1 fs, whilst surface energy calculations were based on 150 ps simulations of model crystallites after equilibration under constant-NVT conditions for 150 ps. The crystallites consisted of between 16 and 40 lamellae, with each layer spanning 49–70 unit cells. The energy of each crystallite, E_{tot}, was calculated as the mean potential energy of the system over 150 ps, after equilibration for an equal period of time. The energy of each face, E_{face}, was estimated from a plot of E_{tot}/N against $1/N$, in accordance with equation S1:[8]

$$\frac{E_{\mathrm{tot}}}{N} = \frac{-E_{\mathrm{face}}}{N} + (E_{\mathrm{bulk}} + E_{\mathrm{face}}) \tag{S1}$$

No bond or angle constraints were applied. The neighbor list was updated every five steps with a cut-off distance of 0.9 nm, while cut-off distances of 1.4 and 1.5 nm were used for the van der Waals and electrostatic interactions respectively. Electrostatics were calculated using the reaction-field method with a relative dielectric constant of 1 inside the reaction field and 78 beyond the cut-off distance.

DFT calculations

DFT calculations were performed in Gaussian 09 using the B3LYP functional and Berny optimization algorithm, with redundant internal coordinates and default settings for the convergence thresholds.[9] Analyses were conducted using the cc-pVDZ basis set,[10] as this was found to produce more realistic (planar) aromatic ring geometries than the more commonly used Pople basis sets.[11] However, the cc-pVDZ basis set does not include a diffuse component, and incorporating such functions by switching to the aug-cc-pVDZ basis set resulted in an unacceptable increase in computation time. Conformational energy landscapes were constructed by optimizing the geometry of a molecule in the relevant crystal structure with no fixed variables, then repeating the optimization with fixed values of selected torsion angles.

1.7 Synthesis

Reagents and solvents were purchased from commercial sources and used without further purification. Reagent and solvent quantities detailed in the general procedures are typical, but the actual masses used may deviate from these values by a constant scaling factor of 0.5-2.0. Yields are scaled according to the reagent quantities listed.

General procedure for bis(urea) synthesis

Compounds **1**-**10** were synthesized by the addition of 1,3-bis(1-isocyanato-1-methylethyl)benzene (0.1 cm^3, 0.43 mmol) to a stirred solution of the necessary amine (0.97 mmol) in chloroform (20 cm^3) under air at 20 °C. In the synthesis of **5b**, the amine was introduced as a hydrochloride salt with triethylamine (2.1 eq.) to aid dissolution. The reaction mixture was left to stand for 24 hours at 20 °C then concentrated *in vacuo* and filtered under suction. The collected solids were washed with chloroform (2 x 20 cm^3) and dried in a drying pistol.

Compound 1a

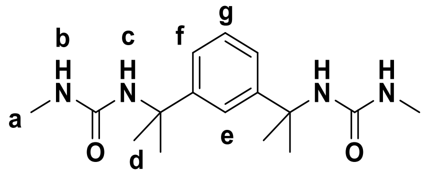

Compound **1a** was obtained as a white solid (125 mg, 0.41 mmol, 94%), m/z (ESI-MS) 329.5 [M+Na]$^+$. ^{1}H NMR (400 MHz, DMSO-d_6) δ 7.34 (t, *J* = 1.9 Hz, 1H, *e*), 7.23 – 7.08 (m, 3H, *f*, *g*), 6.17 (s, 2H, *c*), 5.65 (q, *J* = 4.6 Hz, 2H, *b*), 2.49 (d, *J* = 4.6 Hz, 6H, *a*), 1.52 (s, 12H, *d*). ^{13}C NMR (101 MHz, DMSO-d_6) δ 158.20, 148.92, 127.70, 122.82, 121.84, 54.68, 30.59, 26.49. Elem. Anal. Calc. (%) ($C_{16}H_{26}N_4O_2$) C 62.72, H 8.55, N 18.29; Found (%) C 62.56, H 8.52, N 18.11.

Compound 1b

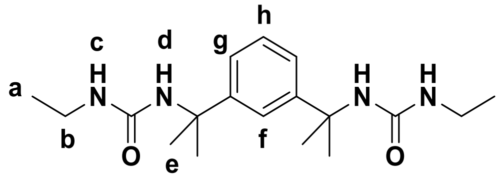

Compound **1b** was obtained as a white solid (129 mg, 0.38 mmol, 89%), m/z (ESI-MS) 357.6 [M+Na]$^+$. ^{1}H NMR (400 MHz, DMSO-d_6) δ 7.34 (t, *J* = 1.8 Hz, 1H, *f*), 7.23 – 7.10 (m, 3H, *g*, *h*), 6.12 (s, 2H, *d*), 5.73 (t, *J* = 5.6 Hz, 2H, *c*), 2.94 (dq, *J* = 7.2, 5.6 Hz, 4H, *b*), 1.52 (s, 12H, *e*), 0.96 (t, *J* = 7.2 Hz, 6H, *a*). ^{13}C NMR (101 MHz, DMSO-d_6) δ 157.52, 148.95, 127.70, 122.76, 121.79, 54.67, 34.16, 30.64, 16.23. Elem. Anal. Calc. (%) ($C_{18}H_{30}N_4O_2$) C 64.64, H 9.04, N 16.75; Found (%) C 64.37, H 8.92, N 16.63.

Compound 1c

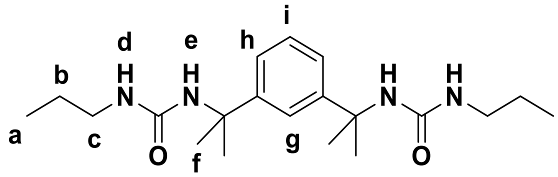

Compound **1c** was obtained as a white solid (120 mg, 0.33 mmol, 76%), m/z (ESI-MS) 385.8 $[M+Na]^+$. ^1H NMR (400 MHz, DMSO-d_6) δ 7.34 (t, *J* = 1.8 Hz, 1H, *g*), 7.23 – 7.11 (m, 3H, *h*, *i*), 6.14 (s, 2H, *e*), 5.79 (t, *J* = 5.8 Hz, 2H, *d*), 2.89 (dt, *J* = 7.0, 5.8 Hz, 4H, *c*), 1.52 (s, 12H, *f*), 1.34 (tq, *J* = 7.4, 7.0 Hz, 4H, *b*), 0.82 (d, *J* = 7.4 Hz, 6H, *a*). ^{13}C NMR (101 MHz, DMSO-d_6) δ 157.61, 148.97, 127.69, 122.75, 121.77, 54.65, 41.15, 30.63, 23.76, 11.81. Elem. Anal. Calc. (%) ($C_{20}H_{34}N_4O_2$) C 66.26, H 9.45, N 15.46; Found (%) C 66.40, H 9.43, N 15.46.

Compound 1d

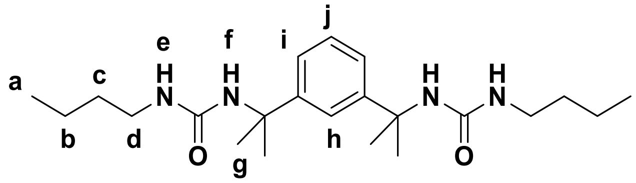

Compound **1d** was obtained as a white solid (152 mg, 0.39 mmol, 90%), m/z (ESI-MS) 413.7 $[M+Na]^+$. ^1H NMR (400 MHz, DMSO-d_6) δ 7.33 (t, *J* = 1.8 Hz, 1H, *h*), 7.20 – 7.08 (m, 3H, *i*, *j*), 6.12 (s, 2H, *f*), 5.75 (t, *J* = 5.7 Hz, 2H, *e*), 2.92 (dt, *J* = 6.3, 5.7 Hz, 4H, *d*), 1.51 (s, 12H, *g*), 1.38 – 1.18 (m, 8H, *b*, *c*), 0.95 – 0.73 (m, 6H, *a*). ^{13}C NMR (101 MHz, DMSO-d_6) δ 157.59, 148.97, 127.68, 122.75, 121.75, 54.65, 38.96, 32.71, 30.63, 19.99, 14.17. Elem. Anal. Calc. (%) ($C_{22}H_{38}N_4O_2$) C 67.66, H 9.81, N 14.35; Found (%) C 67.40, H 9.72, N 14.27.

Compound 1e

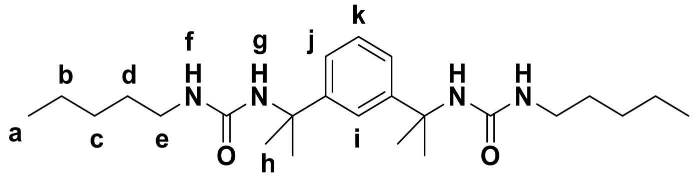

Compound **1e** was obtained as a white solid (140 mg, 0.33 mmol, 77%), m/z (ESI-MS) 441.3 $[M+Na]^+$. ^1H NMR (400 MHz, DMSO-d_6) δ 7.34 (t, *J* = 2.0 Hz, 1H, *i*), 7.22 – 7.10 (m, 3H, *j*, *k*), 6.12 (s, 2H, *g*), 5.76 (t, *J* = 5.7 Hz, 2H, *f*), 2.91 (dt, *J* = 6.6, 5.7 Hz, 4H, *e*), 1.51 (s, 12H, *h*), 1.39 – 1.13 (m, 12H, *b*, *c*, *d*), 0.88 (s, 6H, *a*). ^{13}C NMR (101 MHz, DMSO-d_6) δ 157.60, 148.97, 127.67, 122.76, 121.78, 54.67, 39.28, 30.65, 30.25, 29.10, 22.36, 14.43. Elem. Anal. Calc. (%) ($C_{24}H_{42}N_4O_2$) C 68.86, H 10.11, N 13.38; Found (%) C 68.69, H 10.11, N 13.33.

Compound 1f

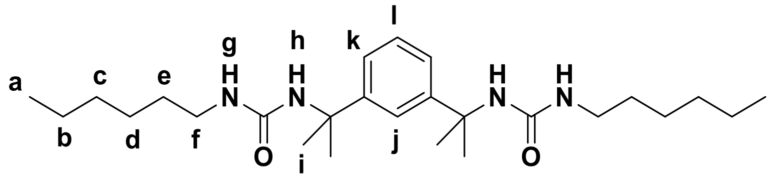

Compound **1f** was obtained as a white solid (171 mg, 0.38 mmol, 88%), m/z (ESI-MS) 469.7 [M+Na]+. ^{1}H NMR (400 MHz, DMSO-d_6) δ 7.33 (t, *J* = 2.0 Hz, 1H, *j*), 7.22 – 7.10 (m, 3H, *k*, *l*), 6.13 (s, 2H, *h*), 5.76 (t, *J* = 5.7 Hz, 2H, *g*), 2.91 (dt, *J* = 6.4, 5.7 Hz, 4H, *f*), 1.51 (s, 12H, *i*), 1.38 – 1.15 (m, 16H, *b*, *c*, *d*, *e*), 0.85 (s, 6H, *a*). ^{13}C NMR (101 MHz, DMSO-d_6) δ 157.58, 148.96, 127.65, 122.75, 121.77, 54.66, 39.31, 31.52, 30.65, 30.54, 26.53, 22.57, 14.39. Elem. Anal. Calc. (%) ($C_{26}H_{46}N_4O_2$) C 69.91, H 10.38, N 12.54; Found (%) C 69.70, H 10.22, N 12.42.

Compound 2a

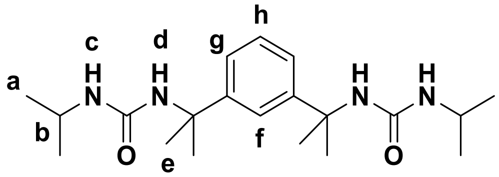

Compound **2a** was obtained as a white solid (128 mg, 0.35 mmol, 81%), m/z (ESI-MS) 385.7 [M+Na]+. ^{1}H NMR (400 MHz, DMSO-d_6) δ 7.34 (t, *J* = 1.8 Hz, 1H, *f*), 7.23 – 7.10 (m, 3H, *g*, *h*), 6.05 (s, 2H, *d*), 5.64 (d, *J* = 7.7 Hz, 2H, *c*), 3.58 (dspt, *J* = 7.7, 6.6 Hz, 2H, *b*), 1.51 (s, 12H, *e*), 1.00 (d, *J* = 6.6 Hz, 12H, *a*). ^{13}C NMR (101 MHz, DMSO-d_6) δ 157.03, 148.99, 127.70, 122.73, 121.77, 79.65, 54.68, 40.95, 30.69, 23.77. Elem. Anal. Calc. (%) ($C_{20}H_{34}N_4O_2$) C 66.26, H 9.45, N 15.46; Found (%) C 66.22, H 9.46, N 15.42.

Compound 2b

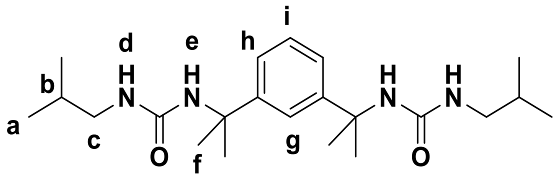

Compound **2b** was obtained as a white solid (134 mg, 0.34 mmol, 79%), m/z (ESI-MS) 413.8 [M+Na]+. ^{1}H NMR (400 MHz, DMSO-d_6) δ 7.34 (t, *J* = 1.9 Hz, 1H, *g*), 7.23 – 7.10 (m, 3H, *h*, *i*), 6.15 (s, 2H, *e*), 5.83 (t, *J* = 5.9 Hz, 2H, *d*), 2.76 (dd, *J* = 6.2, 5.9 Hz, 4H, *c*), 1.64 – 1.53 (m, 2H, *b*), 1.52 (s, 12H, *f*), 0.82 (d, *J* = 6.7 Hz, 12H, *a*). ^{13}C NMR (101 MHz, DMSO-d_6) δ 157.62, 148.99, 127.69, 122.76, 121.76, 54.64, 46.91, 30.64, 29.17, 20.50. Elem. Anal. Calc. (%) ($C_{22}H_{38}N_4O_2$) C 67.66, H 9.81, N 14.35; Found (%) C 67.52, H 9.75, N 14.29.

Compound 2c

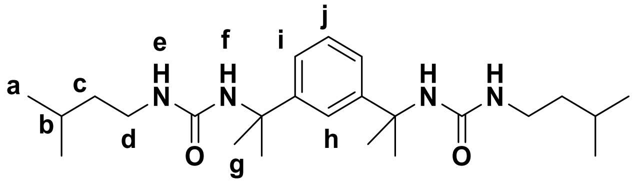

Compound **2c** was obtained as a white solid (117 mg, 0.28 mmol, 64%), m/z (ESI-MS) 441.8 [M+Na]+. ^{1}H NMR (400 MHz, DMSO-d_6) δ 7.33 (t, *J* = 1.8 Hz, 1H, *h*), 7.20 – 7.11 (m, 3H, *i*, *j*), 6.11 (s, 2H, *f*), 5.73 (t, *J* = 5.7 Hz, 2H, *e*), 2.94 (dt, *J* = 7.7, 5.7 Hz, 4H, *d*), 1.64 – 1.54 (m, 2H, *b*), 1.51 (s, 12H, *g*), 1.23 (dt, *J* = 7.7, 7.0 Hz, 4H, *c*), 0.85 (d, *J* = 6.6 Hz, 12H, *a*). ^{13}C NMR (101 MHz, DMSO-d_6) δ 157.58, 148.98, 127.69,

122.76, 121.76, 54.68, 39.63, 37.53, 30.65, 25.58, 22.89. Elem. Anal. Calc. (%) ($C_{24}H_{42}N_4O_2$) C 68.86, H 10.11, N 13.38; Found (%) C 68.64, H 10.09, N 13.32.

Compound 3

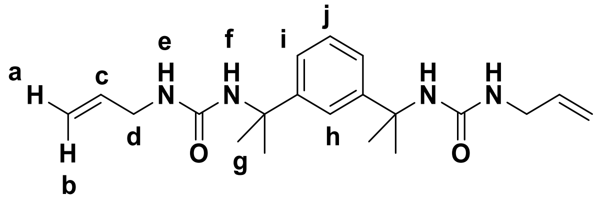

Compound **3** was obtained as a white solid (149 mg, 0.42 mmol, 96%), m/z (ESI-MS) 381.7 $[M+Na]^+$. ^{1}H NMR (400 MHz, DMSO-d_6) δ 7.35 (t, *J* = 1.9 Hz, 1H, *h*), 7.22 – 7.12 (m, 3H, *i, j*), 6.26 (s, 2H, *f*), 5.90 (t, *J* = 5.9 Hz, 2H, *e*), 5.80 (ddt, *J* = 17.2, 10.2, 5.0 Hz, 2H, *c*), 5.12 (dq, *J* = 17.2, 1.8 Hz, 2H, *b*), 5.01 (dq, *J* = 10.2, 1.8 Hz, 2H, *a*), 3.58 (ddt, *J* = 5.9, 5.0, 1.8 Hz, 4H, *d*), 1.53 (s, 12H, *g*). ^{13}C NMR (101 MHz, DMSO-d_6) δ 157.31, 148.88, 137.39, 127.74, 122.81, 121.78, 114.59, 54.74, 41.77, 30.59. Elem. Anal. Calc. (%) ($C_{20}H_{30}N_4O_2$) C 67.01, H 8.44, N 15.63; Found (%) C 66.71, H 8.38, N 15.53.

Compound 4

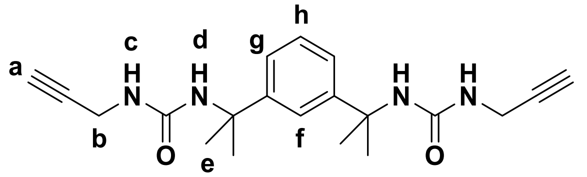

Compound **4** was obtained as a white solid (148 mg, 0.42 mmol, 96%), m/z (ESI-MS) 377.6 $[M+Na]^+$. ^{1}H NMR (400 MHz, DMSO-d_6) δ 7.34 (t, *J* = 1.7 Hz, 1H, *f*), 7.26 – 7.10 (m, 3H, *g*, *h*), 6.32 (s, 2H, *d*), 6.09 (t, *J* = 5.7 Hz, 2H, *c*), 3.74 (dd, *J* = 5.7, 2.5 Hz, 4H, *b*), 3.04 (t, *J* = 2.5 Hz, 2H, *a*), 1.53 (s, 12H, *e*). ^{13}C NMR (101 MHz, DMSO-d_6) δ 156.90, 148.67, 127.80, 122.87, 121.79, 83.01, 72.96, 54.92, 30.51, 28.93. Elem. Anal. Calc. (%) ($C_{20}H_{26}N_4O_2$) C 67.77, H 7.39, N 15.81; Found (%) C 67.25, H 7.35, N 15.73.

Compound 5b

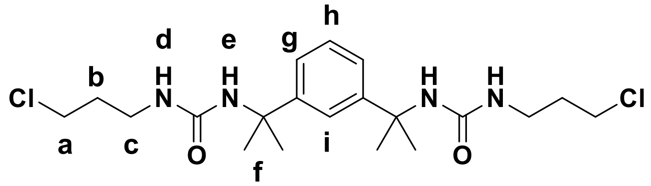

Compound **5b** was obtained as a white solid (72 mg, 69%), m/z 453.3 $[M+Na]^+$. ^{1}H NMR (400 MHz, DMSO-d_6) 7.33 (t, J = 1.8 Hz, 1H, *f*), 7.24 – 7.10 (m, 3H, *g*, *h*), 6.18 (s, 2H, *e*), 5.90 (t, J = 5.9 Hz, 2H, *d*), 3.60 (t, J = 6.6 Hz, 4H, *a*), 3.04 (dt, J = 5.9, 6.4 Hz, 4H, *c*), 1.78 (tt, J = 6.4, 6.6 Hz, 4H, *b*), 1.51 (s, 12H, *f*). ^{13}C NMR (101 MHz, DMSO-d_6) 157.69, 148.92, 127.74, 122.76, 121.75, 54.74, 43.57, 36.81, 33.54, 30.60. Elem. Anal. Calc. (%) ($C_{20}H_{32}N_4O_2Cl_2$) C 55.68, H 7.48, N 12.99; Found (%) C 55.82, H 7.47, N 13.10.

Compound 5c

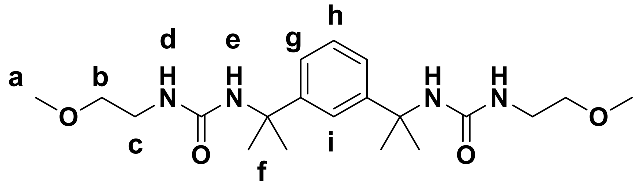

Compound **5c** is soluble in chloroform. After evaporation of the reaction mixture and washing with cold chloroform, the compound was obtained as a white solid (127 mg, 75%), m/z 417.4 $[M+Na]^+$. ^1H NMR (400 MHz, DMSO-d_6) 7.32 (t, J = 1.8 Hz, 1H, *i*), 7.22 – 7.09 (m, 3H, *g*, *h*), 6.31 (s, 2H, *e*), 5.89 (t, J = 5.7 Hz, 2H, *d*), 3.26 (m, 10H, *a*, *b*), 3.08 (q, J = 5.7 Hz, 4H, *c*), 1.51 (s, 12H, *f*). ^{13}C NMR (101 MHz, DMSO-d_6) 157.55, 148.87, 127.72, 122.75, 121.77, 72.19, 58.36, 54.71, 39.18, 30.62. Elem. Anal. Calc. (%) ($C_{20}H_{34}N_4O_4$) C 60.89, H 8.69, N 14.20; Found (%) C 60.97, H 8.80, N 14.32.

Compound 6

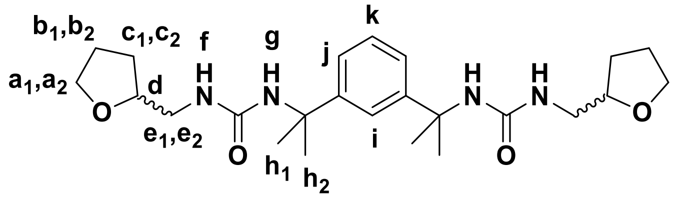

Compound **6** was synthesised from racemic tetrahydrofurfurylamine as a mixture of diastereomers. The product was obtained as a white solid (120 mg, 0.27 mmol, 62%), m/z (ESI-MS) 469.9 $[M+Na]^+$. ^1H NMR (400 MHz, DMSO-d_6) δ 7.33 (t, *J* = 1.9 Hz, 1H, *i*), 7.22 – 7.10 (m, 3H, *j*, *k*), 6.32 (s, 2H, *g*), 5.89 (t, *J* = 5.9 Hz, 2H, *f*), 3.81 – 3.69 (m, 4H, a_1, *d*), 3.61 (dt, *J* = 6.9, 6.4 Hz, 2H, a_2), 3.12 – 3.01 (m, 2H, e_2), 3.00-2.90 (m, 2H, e_1), 1.81 (m, 6H, b_1, b_2, c_2), 1.52 (s, 6H, h_2), 1.51 (s, 6H, h_1), 1.50 – 1.41 (m, 2H, c_1). ^{13}C NMR (101 MHz, DMSO-d_6) δ 157.57, 148.92, 127.67, 122.73, 121.75, 78.50, 67.60, 54.68, 43.38, 30.75, 30.72, 30.54, 30.51, 28.55, 25.83. Elem. Anal. Calc. (%) ($C_{24}H_{38}N_4O_4$) C 64.55, H 8.58, N 12.55; Found (%) C 64.17, H 8.46, N 12.23.

Compound 7a

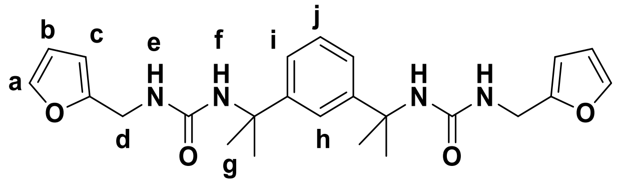

Compound **7a** was obtained as a white solid (195 mg, 0.36 mmol, 83%), m/z (ESI-MS) 461.6 $[M+Na]^+$. ^1H NMR (400 MHz, DMSO-d_6) δ 7.56 (dd, *J* = 1.8, 0.9 Hz, 2H, *a*), 7.35 (t, *J* = 1.9 Hz, 1H, *h*), 7.21 – 7.06 (m, 3H, *i*, *j*), 6.38 (dd, *J* = 3.1, 1.8 Hz, 2H, *b*), 6.30 (s, 2H, *f*), 6.19 (t, *J* = 5.8 Hz, 2H, *e*), 6.17 (dd, *J* = 3.1, 0.9 Hz, 2H, *c*), 4.13 (d, *J* = 5.8 Hz, 4H, *d*), 1.53 (s, 12H, *g*). ^{13}C NMR (101 MHz, DMSO-d_6) δ 157.14, 154.21, 148.76, 142.27, 127.75, 122.83, 121.77, 110.83, 106.46, 54.82, 36.52, 30.53. Elem. Anal. Calc. (%) ($C_{24}H_{30}N_4O_4$) C 65.73, H 6.90, N 12.78; Found (%) C 65.69, H 6.92, N 12.81.

Compound 7b

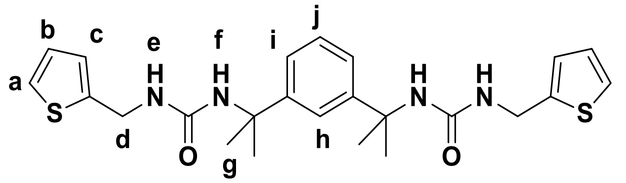

Compound **7b** was obtained as a white solid (184 mg, 0.39 mmol, 90%), m/z (ESI-MS) 493.8 [M+Na]$^+$. ^{1}H NMR (400 MHz, DMSO-d_6) δ 7.39 – 7.33 (m, 3H, *a*, *h*), 7.23 – 7.12 (m, 3H, *i*, *j*), 6.98 – 6.88 (m, 4H, *b*, *c*), 6.32 (t, *J* = 6.0 Hz, 2H, *e*), 6.31 (s, 2H, *f*), 4.32 (d, *J* = 6.0 Hz, 4H, *d*), 1.54 (s, 12H, *g*). ^{13}C NMR (101 MHz, DMSO-d_6) δ 157.15, 148.76, 144.95, 127.73, 127.06, 125.03, 124.93, 122.85, 121.77, 54.86, 38.26, 30.60. Elem. Anal. Calc. (%) ($C_{24}H_{30}N_4O_2S_2$) C 61.25, H 6.42, N 11.90; Found (%) C 61.27, H 6.40, N 11.90.

Compound 8

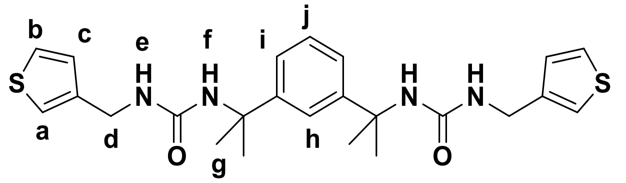

Compound **8** was obtained as a white solid (164 mg, 0.35 mmol, 80%), m/z (ESI-MS) 493.6 [M+Na]$^+$. ^{1}H NMR (400 MHz, DMSO-d_6) δ 7.47 (dd, *J* = 4.9, 2.9 Hz, 2H, *b*), 7.37 (t, *J* = 1.9 Hz, 1H, *h*), 7.25 – 7.13 (m, 5H, *a*, *i*, *j*), 6.99 (dd, *J* = 4.9, 1.3 Hz, 2H, *c*), 6.28 (s, 2H, *f*), 6.20 (t, *J* = 5.9 Hz, 2H, *e*), 4.13 (d, *J* = 5.9 Hz, 4H, *d*), 1.54 (s, 12H, *g*). ^{13}C NMR (101 MHz, DMSO-d_6) δ 157.41, 148.87, 142.47, 127.81, 127.74, 126.66, 122.84, 121.76, 121.32, 54.77, 38.72, 30.60. Elem. Anal. Calc. (%) ($C_{24}H_{30}N_4O_2S_2$) C 61.25, H 6.42, N 11.90; Found (%) C 60.90, H 6.41, N 11.84.

Compound 9a

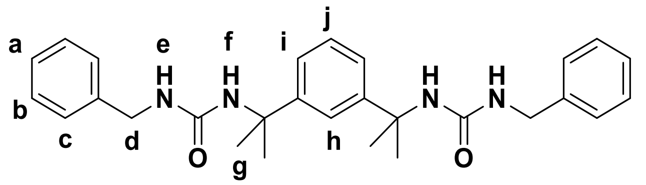

Compound **9a** was obtained as a white solid (117 mg, 0.26 mmol, 59%), m/z (ESI-MS) 481.8 [M+Na]$^+$. ^{1}H NMR (400 MHz, DMSO-d_6) δ 7.40 (t, *J* = 1.9 Hz, 1H, *h*), 7.35 – 7.26 (m, 4H, *b*), 7.26 – 7.14 (m, 9H, *a*, *c*, *i*, *j*), 6.32 (s, 2H, *f*), 6.27 (t, *J* = 6.0 Hz, 2H, *e*), 4.16 (d, *J* = 5.9 Hz, 4H, *d*), 1.55 (s, 12H, *g*). ^{13}C NMR (101 MHz, DMSO-d_6) δ 157.51, 148.90, 141.48, 128.65, 127.73, 127.35, 126.96, 122.83, 121.75, 54.79, 42.99, 30.63. Elem. Anal. Calc. (%) ($C_{28}H_{34}N_4O_2$) C 73.33, H 7.47, N 12.22; Found (%) C 73.24, H 7.46, N 12.22.

Compound 9b

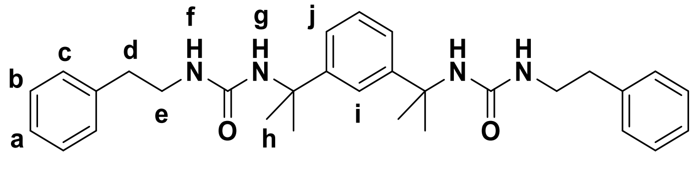

Compound **9b** was obtained as a white solid (185 mg, 0.38 mmol, 87%), m/z (ESI-MS) 509.8 [M+Na]+. ^{1}H NMR (400 MHz, DMSO-d_6) δ 7.34 (t, *J* = 1.9 Hz, 1H, *i*), 7.33 – 7.26 (m, 4H, *b*), 7.23 – 7.16 (m, 7H, *a*, *c*, *k*), 7.16 – 7.11 (m, 2H, *j*), 6.24 (s, 2H, *g*), 5.80 (t, *J* = 5.8 Hz, 2H, *f*), 3.17 (dt, *J* = 7.2, 5.8 Hz, 4H, *e*), 2.64 (t, *J* = 7.2 Hz, 4H, d), 1.52 (s, 12H, *h*). ^{13}C NMR (101 MHz, DMSO-d_6) δ 157.49, 148.93, 140.23, 129.10, 128.74, 127.71, 126.41, 122.77, 121.78, 54.70, 41.04, 36.72, 30.66. Elem. Anal. Calc. (%) ($C_{30}H_{38}N_4O_2$) C 74.04, H 7.87, N 11.51; Found (%) C 73.80, H 7.88, N 11.45.

Compound 9c

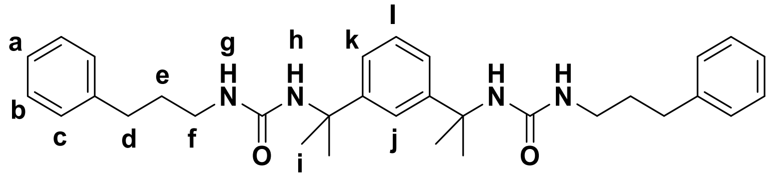

Compound **9c** was obtained as a white solid (177 mg, 0.34 mmol, 79%), m/z (ESI-MS) 537.8 [M+Na]+. ^{1}H NMR (400 MHz, DMSO-d_6) δ 7.35 (t, *J* = 1.9 Hz, 1H, *j*), 7.32 – 7.23 (m, 4H, *b*), 7.22 – 7.09 (m, 9H, *a*, *c*, *k*, *l*), 6.16 (s, 2H, *h*), 5.86 (t, *J* = 5.7 Hz, 2H, *g*), 2.94 (dt, *J* = 6.6, 5.7 Hz, 4H, *f*), 2.55 (t, *J* = 8.1 Hz, 4H, *d*), 1.68 – 1.58 (tt, *J* = 8.1, 6.6 Hz, 4H, *e*), 1.51 (s, 12H, *i*). ^{13}C NMR (101 MHz, DMSO-d_6) δ 157.59, 148.95, 142.26, 128.71, 127.70, 126.12, 125.99, 122.77, 121.77, 54.69, 38.90, 32.96, 32.41, 30.64. Elem. Anal. Calc. (%) ($C_{32}H_{42}N_4O_2$) C 74.67, H 8.23, N 10.89; Found (%) C 74.65, H 8.22, N 10.88.

Compound 10a

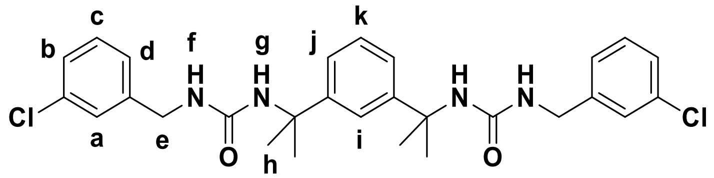

Compound **10a** was obtained as a white solid (211 mg, 0.40 mmol, 92%), m/z (ESI-MS) 549.6 [M+Na]+. ^{1}H NMR (400 MHz, DMSO-d_6) δ 7.38 – 7.25 (m, 7H, *a*, *b*, *c*, *i*), 7.24 – 7.07 (m, 5H, *d*, *j*, *k*), 6.38 (s, 2H, *g*), 6.35 (t, *J* = 6.0 Hz, 2H, *f*), 4.16 (d, *J* = 6.0 Hz, 4H, *e*), 1.53 (s, 12H, *h*). ^{13}C NMR (101 MHz, DMSO-d_6) δ 157.45, 148.84, 144.37, 133.39, 130.50, 127.72, 127.06, 126.84, 125.97, 122.76, 121.72, 54.83, 42.44, 30.64. Elem. Anal. Calc. (%) ($C_{28}H_{32}N_4O_2Cl_2$) C 63.76, H 6.11, N 10.62; Found (%) C 63.77, H 6.08, N 10.63.

Compound 10b

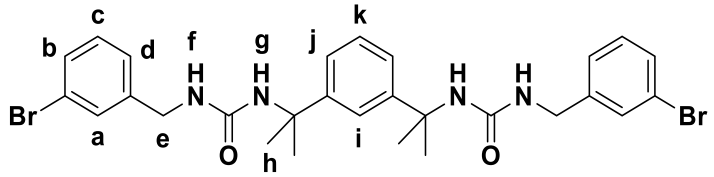

Compound **10b** was obtained as a white solid (233 mg, 0.38 mmol, 87%), m/z (ESI-MS) 639.3 [M+Na]+. ^{1}H NMR (400 MHz, DMSO-d_6) δ 7.45 – 7.38 (m, 4H, *a*, *b*), 7.35 (t, *J* = 2.0 Hz, 1H, *i*), 7.30 – 7.12 (m, 7H, *c*, *d*, *j*, *k*), 6.38 (s, 2H, *g*), 6.34 (t, *J* = 6.0 Hz, 2H, *f*), 4.16 (d, *J* = 6.0 Hz, 4H, *e*), 1.53 (s, 12H, *h*). ^{13}C NMR (101 MHz, DMSO-d_6) δ 157.44, 148.83, 144.64, 130.82, 129.96, 129.74, 127.76, 126.38, 122.74, 122.07,

121.72, 54.84, 42.40, 30.65. Elem. Anal. Calc. (%) ($C_{28}H_{32}N_4O_2Br_2$) C 54.56, H 5.23, N 9.09; Found (%) C 54.65, H 5.25, N 9.10.

Compound 10c

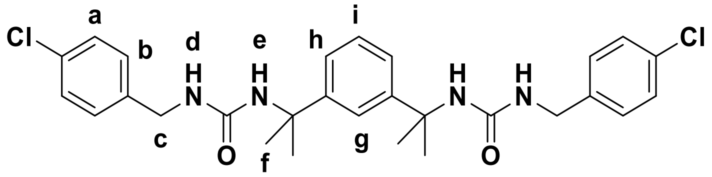

Compound **10c** was obtained as a white solid (188 mg, 0.36 mmol, 82%), m/z (ESI-MS) 550.4 $[M+Na]^+$. ^1H NMR (400 MHz, DMSO-d_6) δ 7.39 – 7.32 (m, 5H, *a*, *g*), 7.24 (d, *J* = 8.5 Hz, 4H, *b*), 7.22 – 7.12 (m, 3H, *h*, *i*), 6.35 (s, 2H, *e*), 6.31 (t, *J* = 6.0 Hz, 2H, *d*), 4.14 (d, *J* = 6.0 Hz, 4H, *c*), 1.52 (s, 12H, *f*). ^{13}C NMR (101 MHz, DMSO-d_6) δ 157.45, 148.84, 140.69, 131.42, 129.19, 128.55, 127.74, 122.80, 121.71, 54.80, 42.33, 30.60. Elem. Anal. Calc. (%) ($C_{28}H_{32}N_4O_2Cl_2$) C 63.76, H 6.11, N 10.62; Found (%) C 63.74, H 6.11, N 10.63.

2. Results

2.1 Characterization

Compound 1a

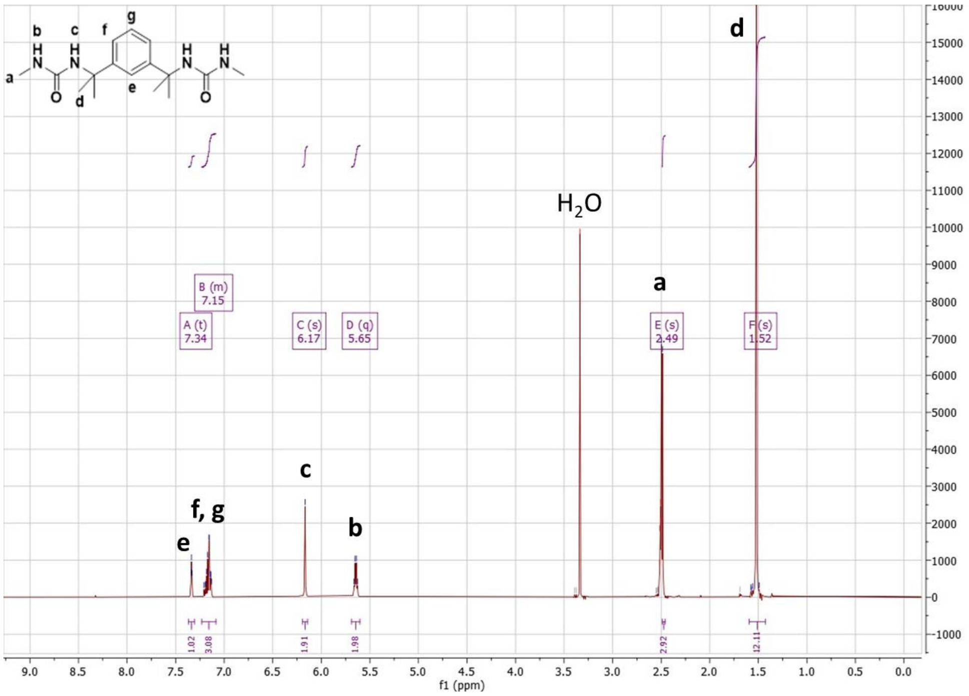

Figure S1 ^{1}H NMR spectrum of **1a** in DMSO-d_6.

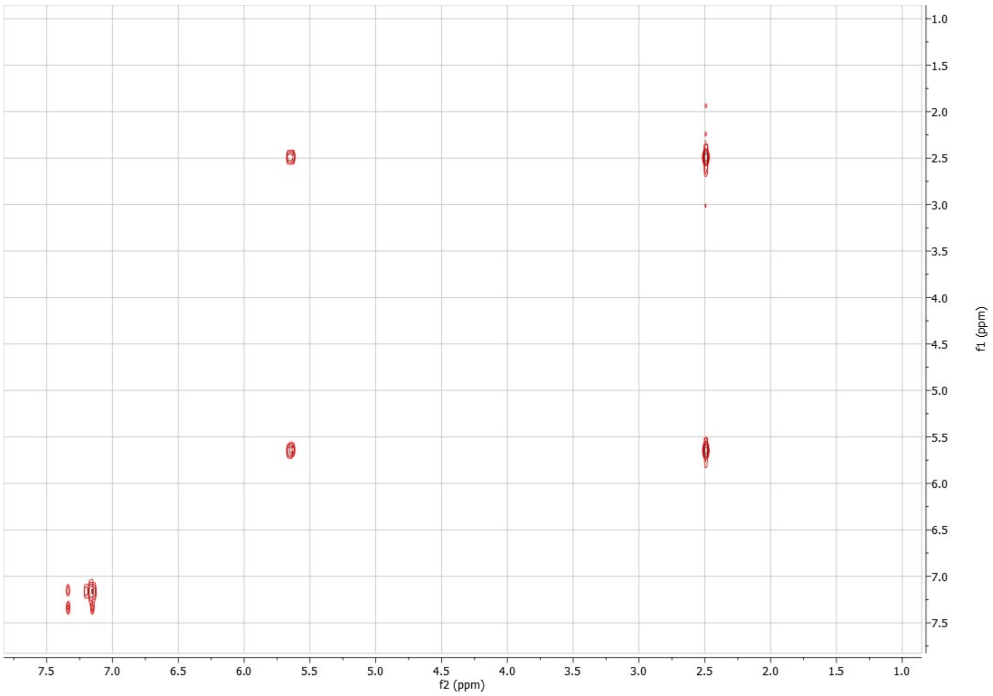

Figure S2 COSY NMR spectrum of **1a** in DMSO-d_6.

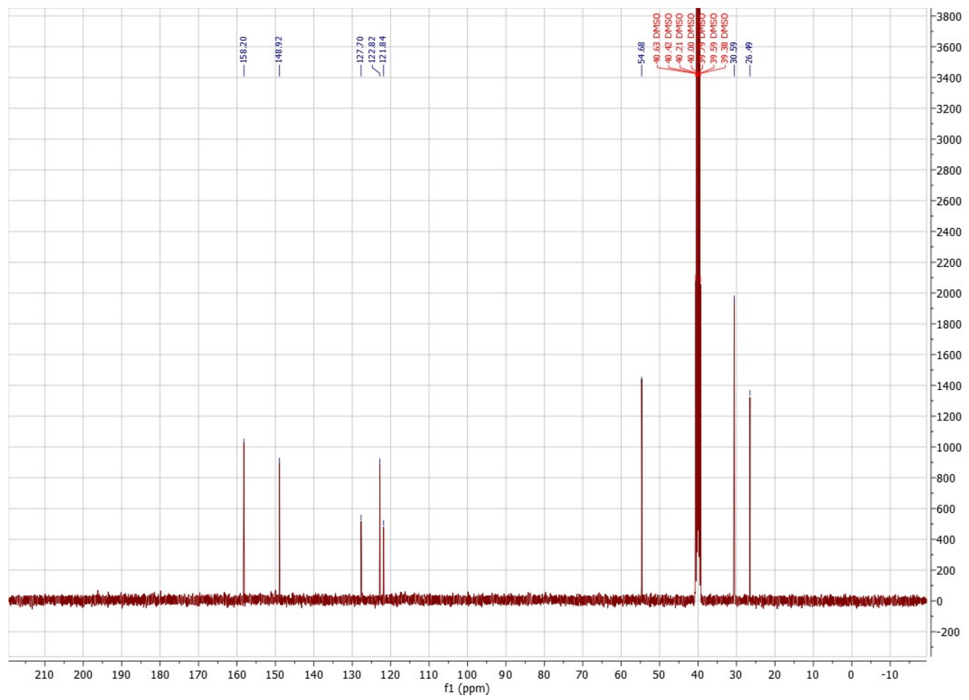

Figure S3 $^{13}C\{^{1}H\}$ NMR spectrum of **1a** in DMSO-d_6.

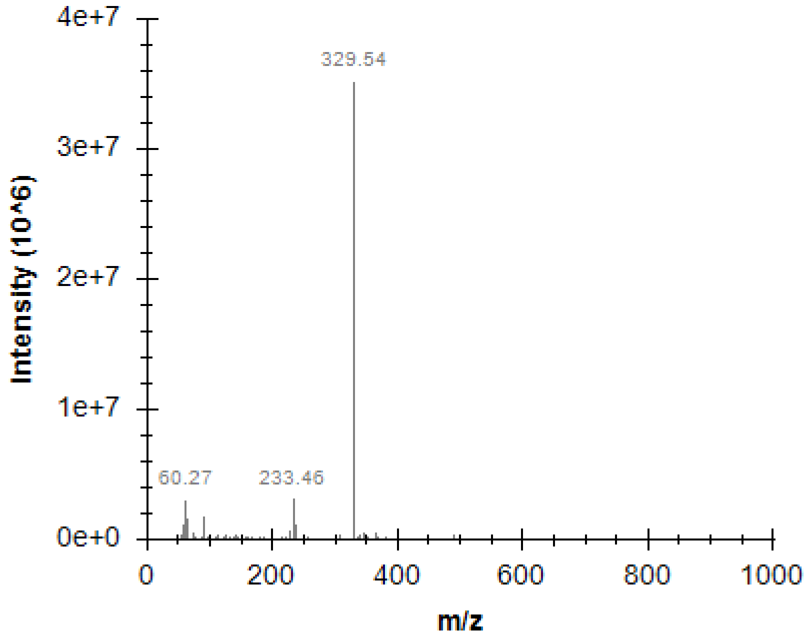

Figure S4 ESI-MS spectrum of **1a** in methanol.

Compound 1b

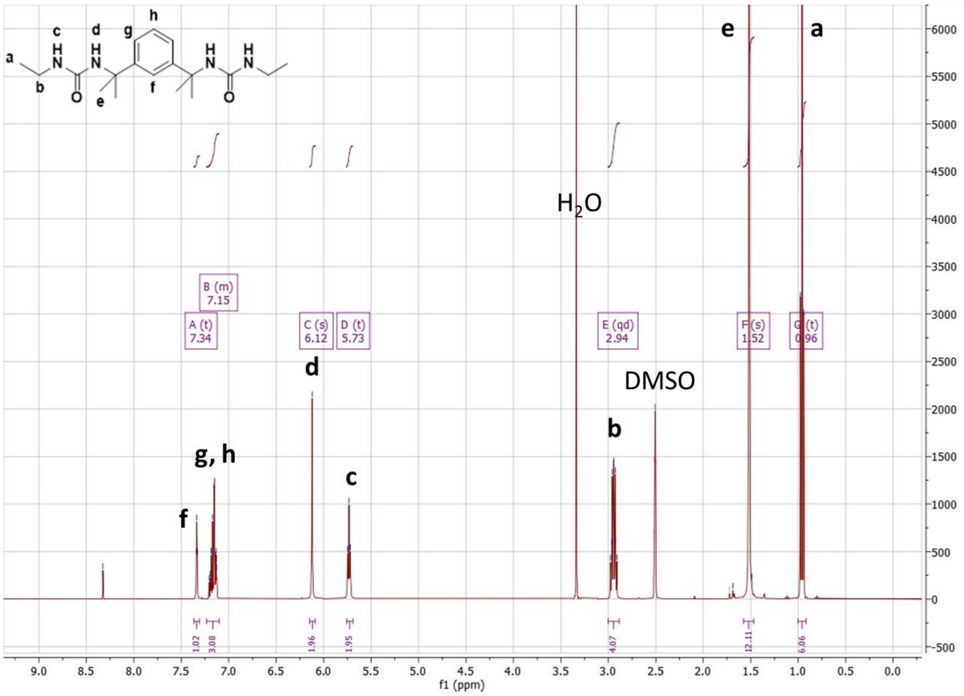

Figure S5 ¹H NMR spectrum of **1b** in DMSO-*d*₆.

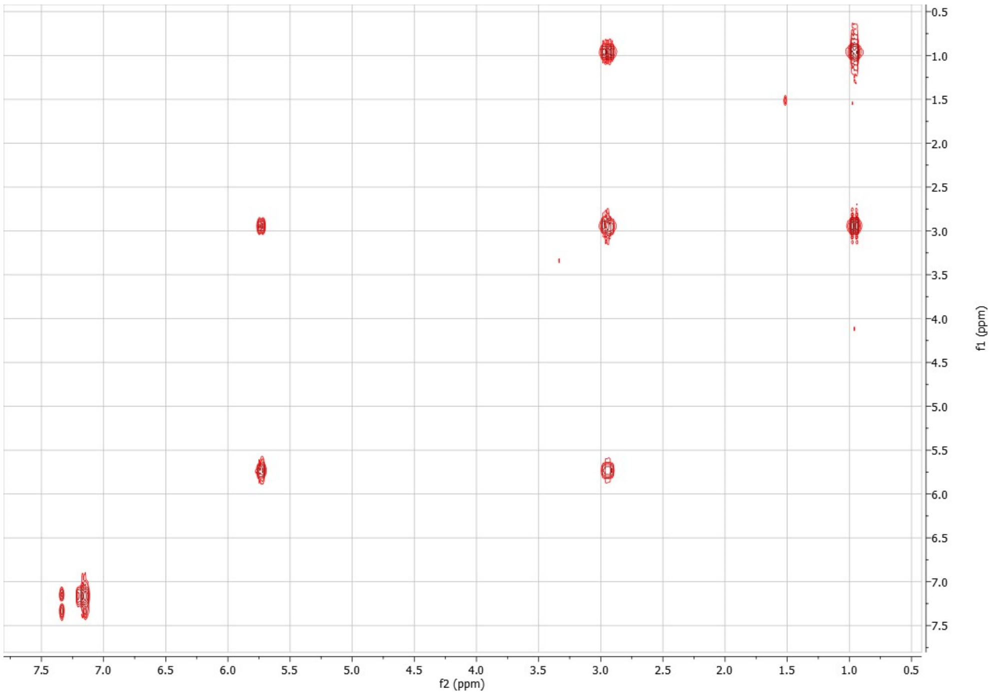

Figure S6 COSY NMR spectrum of **1b** in DMSO-*d*₆.

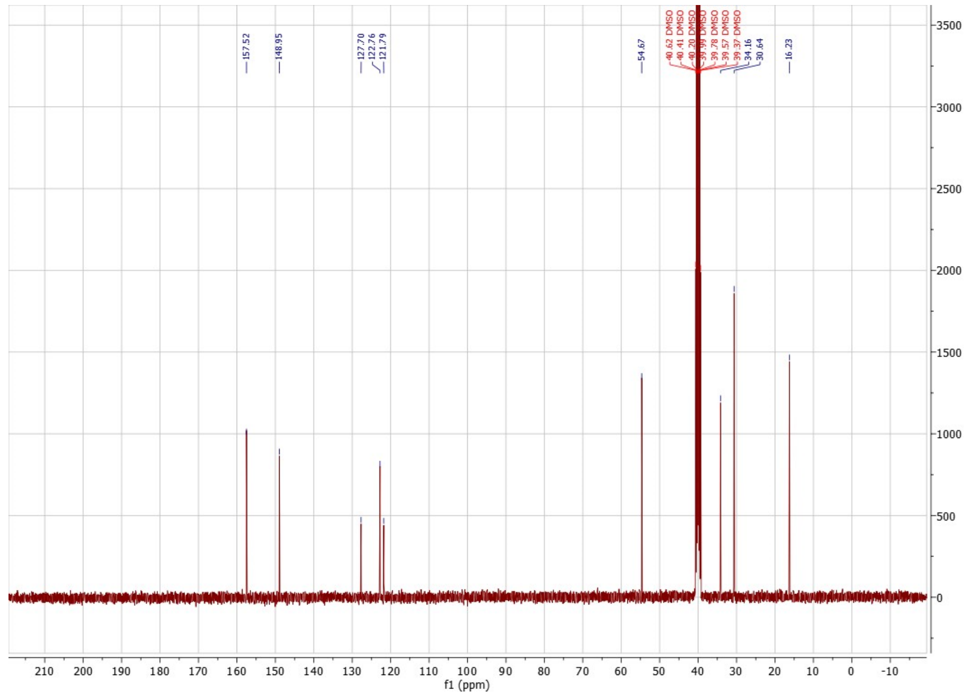

Figure S7 $^{13}C\{^1H\}$ NMR spectrum of **1b** in DMSO-d_6.

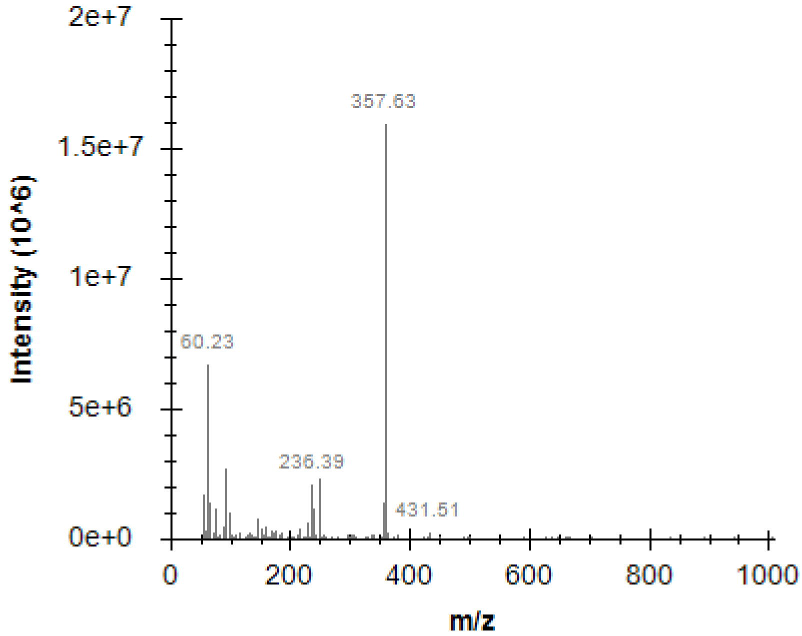

Figure S8 ESI-MS spectrum of **1b** in methanol.

Compound 1c

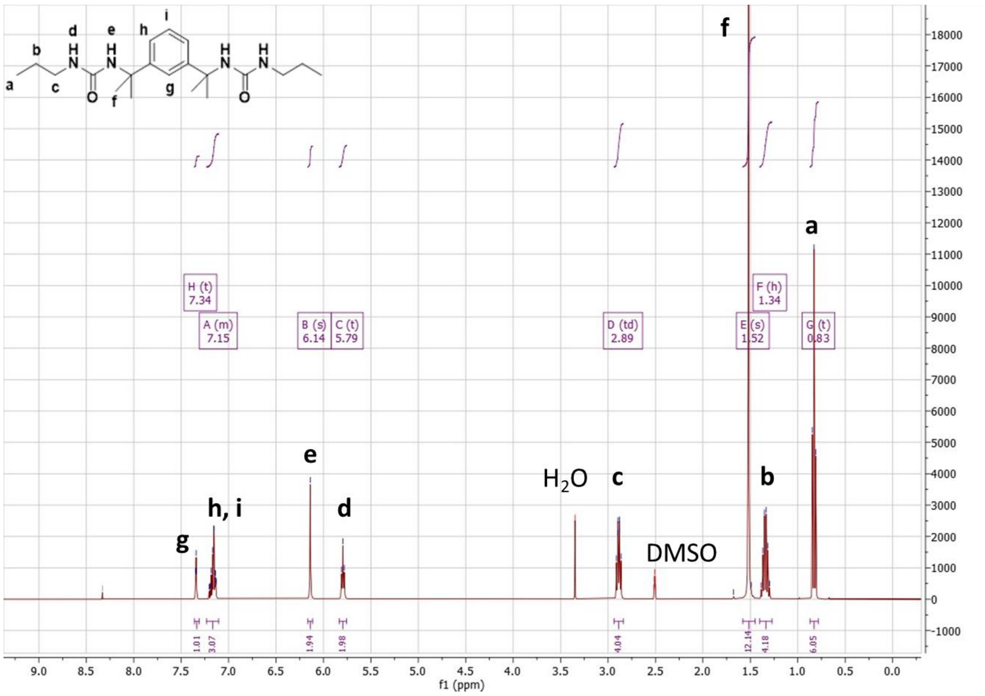

Figure S9 ^{1}H NMR spectrum of **1c** in DMSO-d_6.

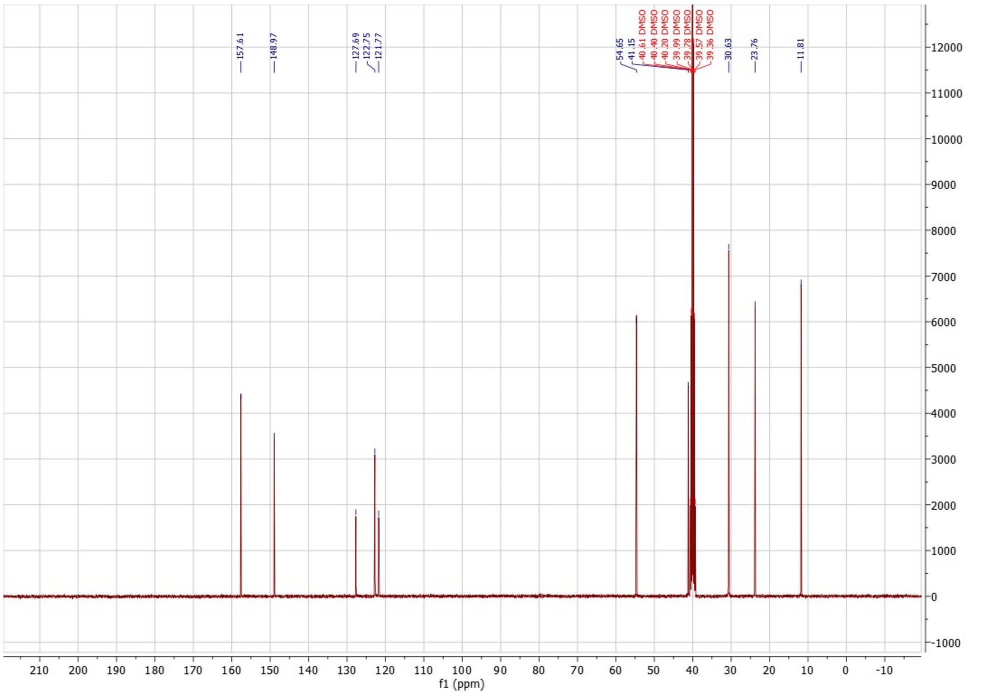

Figure S10 ^{13}C{^{1}H} NMR spectrum of **1c** in DMSO-d_6.

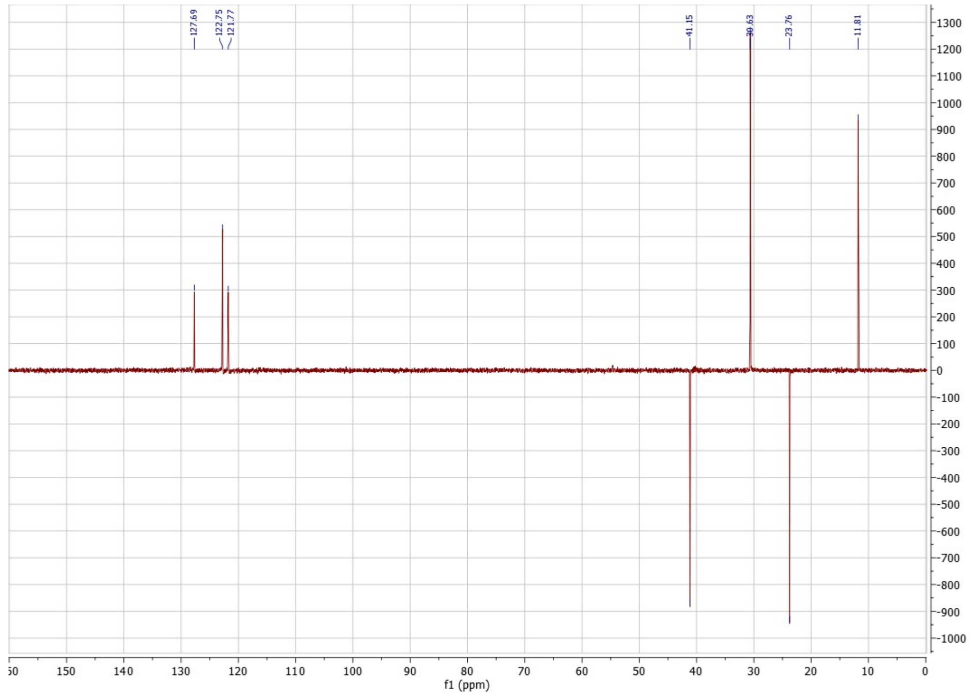

Figure S11 DEPT ^{13}C NMR spectrum of **1c** in DMSO-d_6.

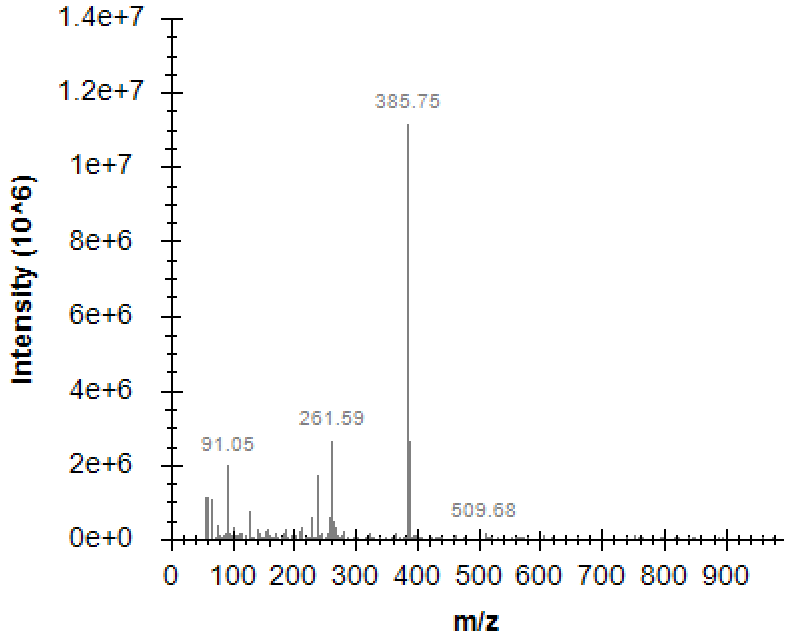

Figure S12 ESI-MS spectrum of **1c** in methanol.

Compound 1d

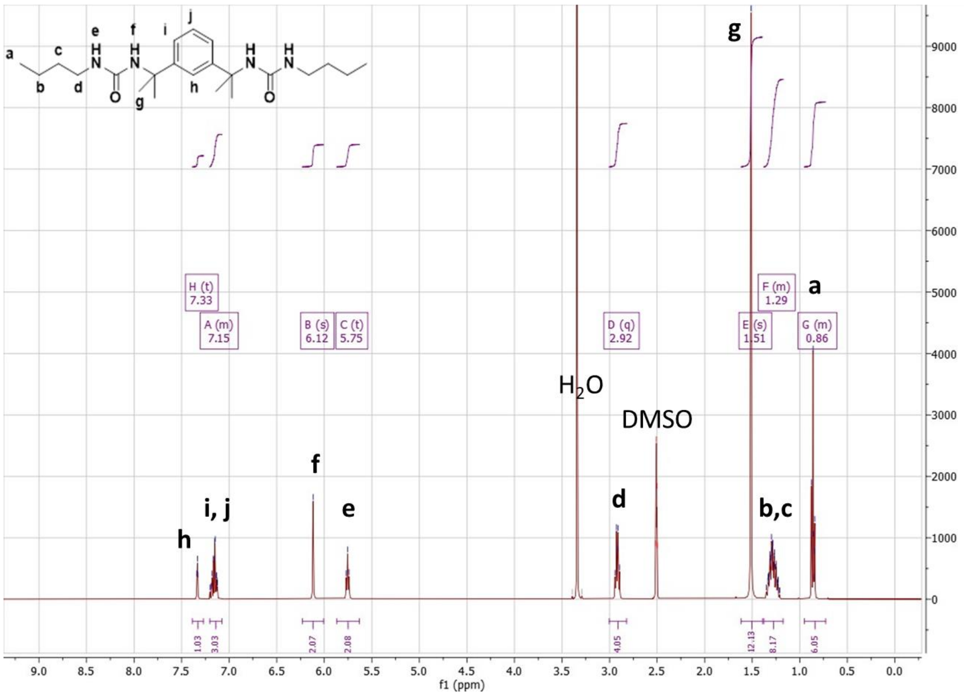

Figure S13 ^{1}H NMR spectrum of **1d** in DMSO-d_6.

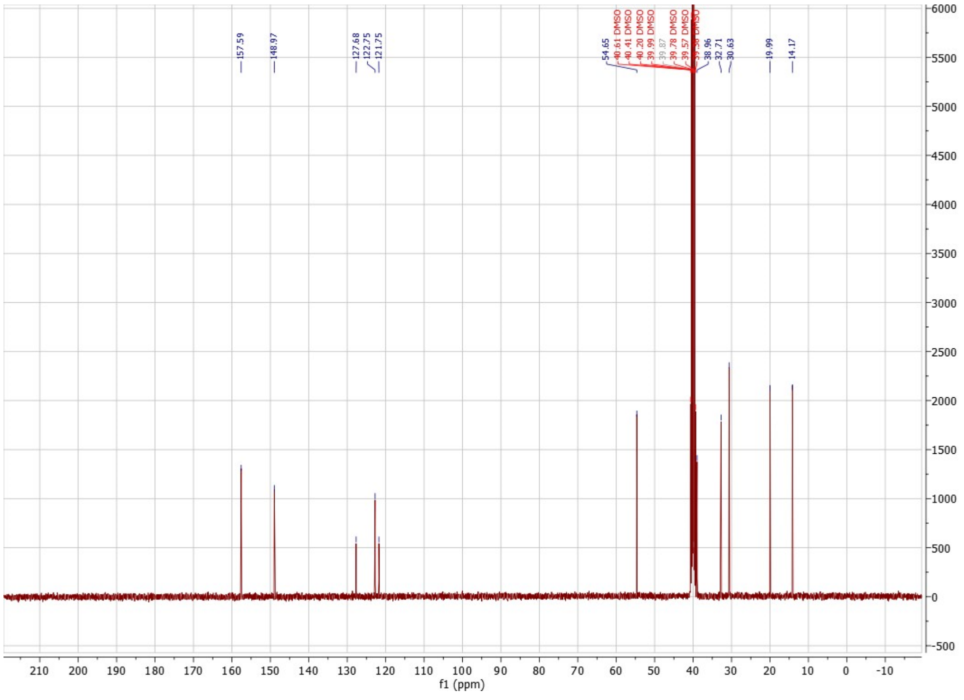

Figure S14 $^{13}C\{^{1}H\}$ NMR spectrum of **1d** in DMSO-d_6.

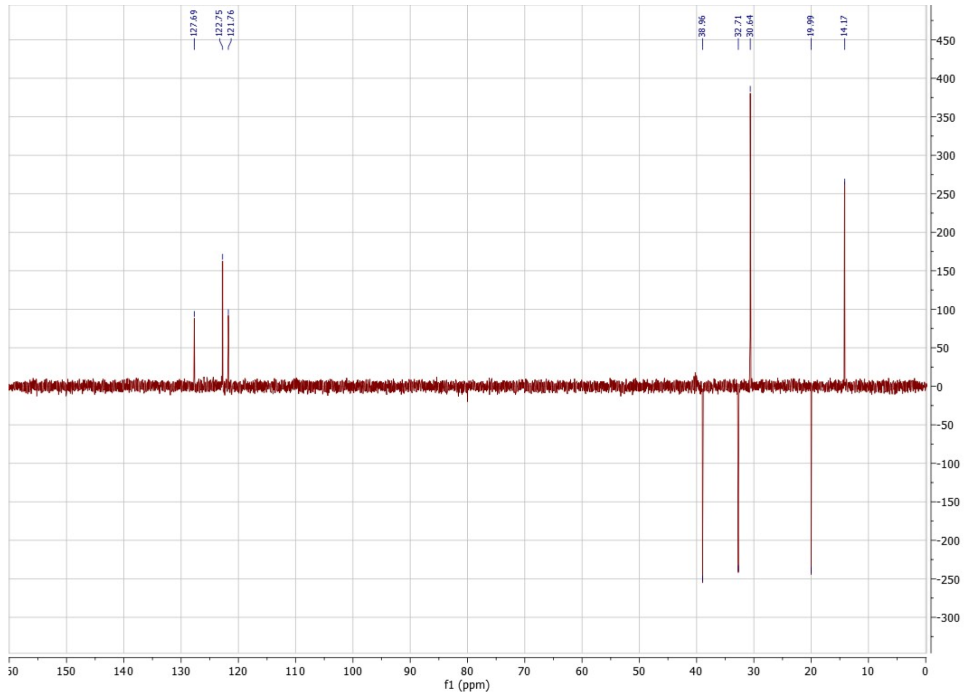

Figure S15 DEPT ^{13}C NMR spectrum of **1d** in DMSO-d_6.

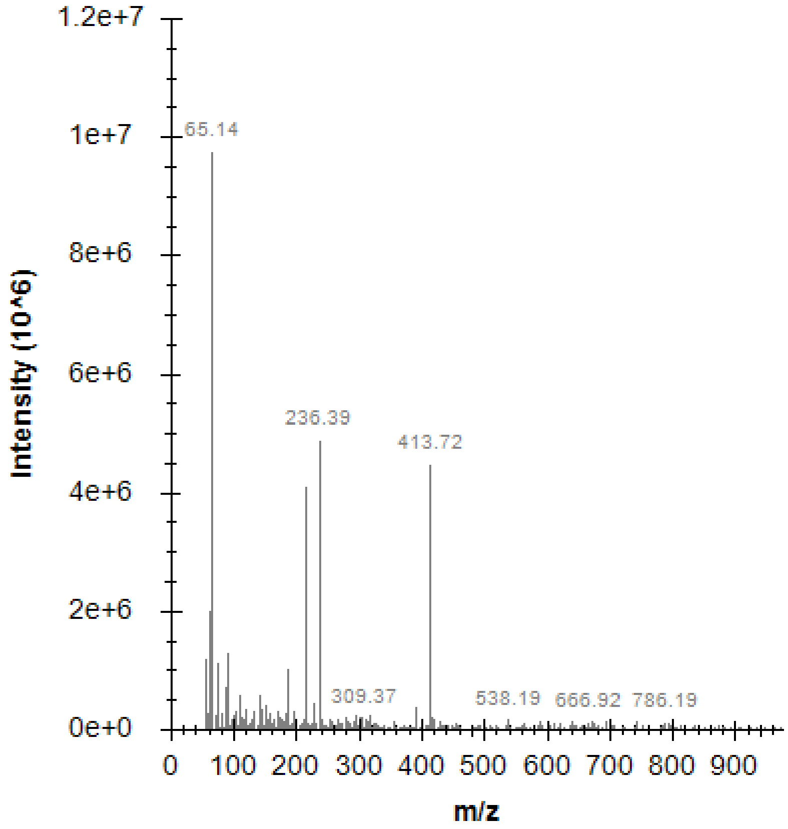

Figure S16 ESI-MS spectrum of **1d** in methanol.

Compound 1e

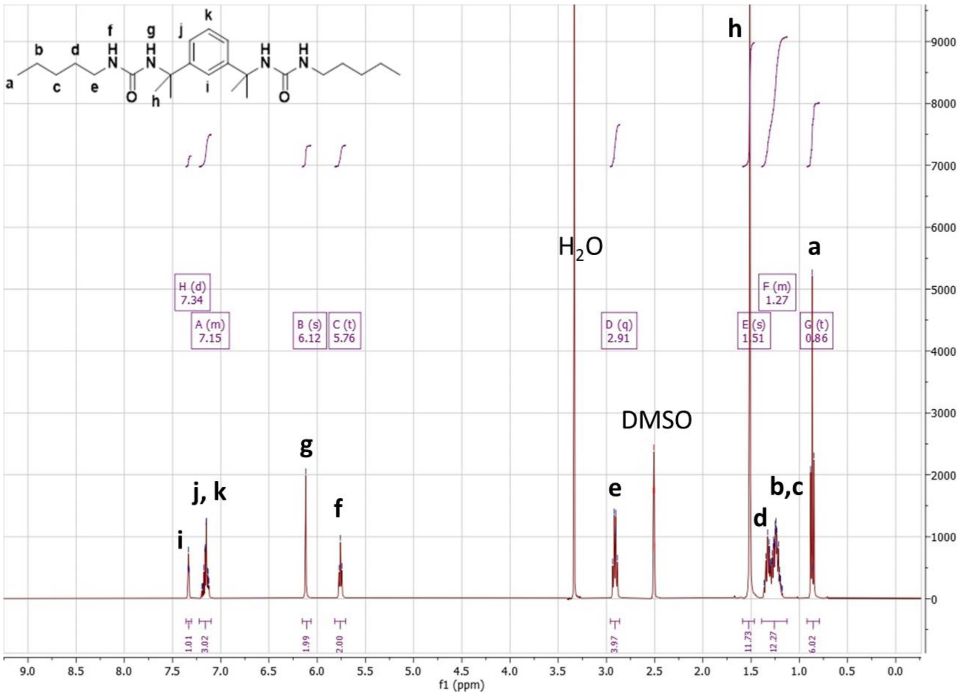

Figure S17 ^{1}H NMR spectrum of **1e** in DMSO-d_6.

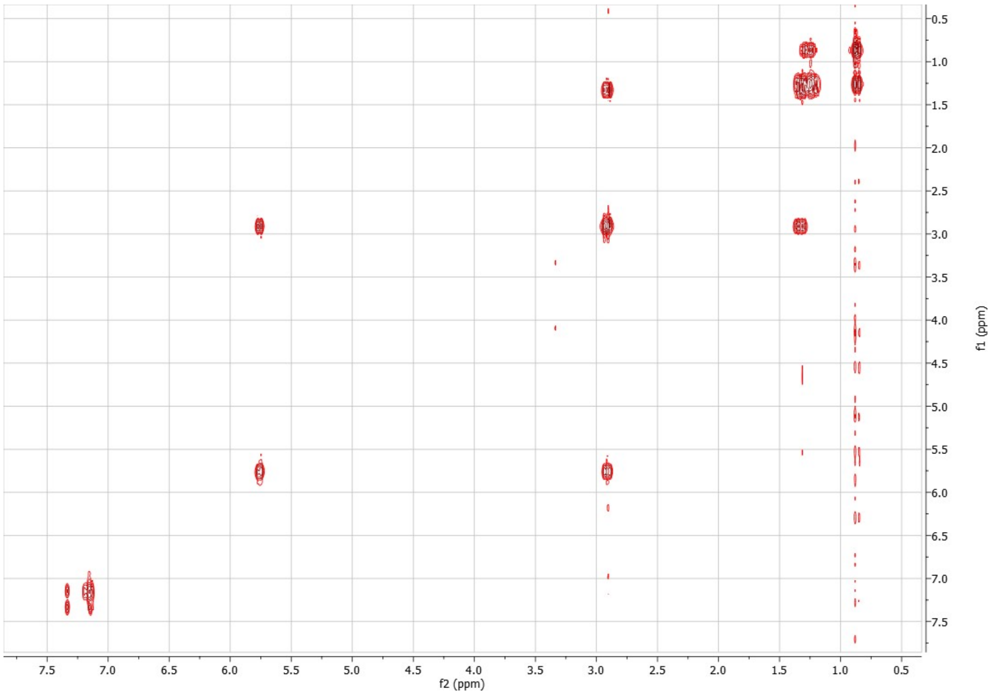

Figure S18 COSY NMR spectrum of **1e** in DMSO-d_6.

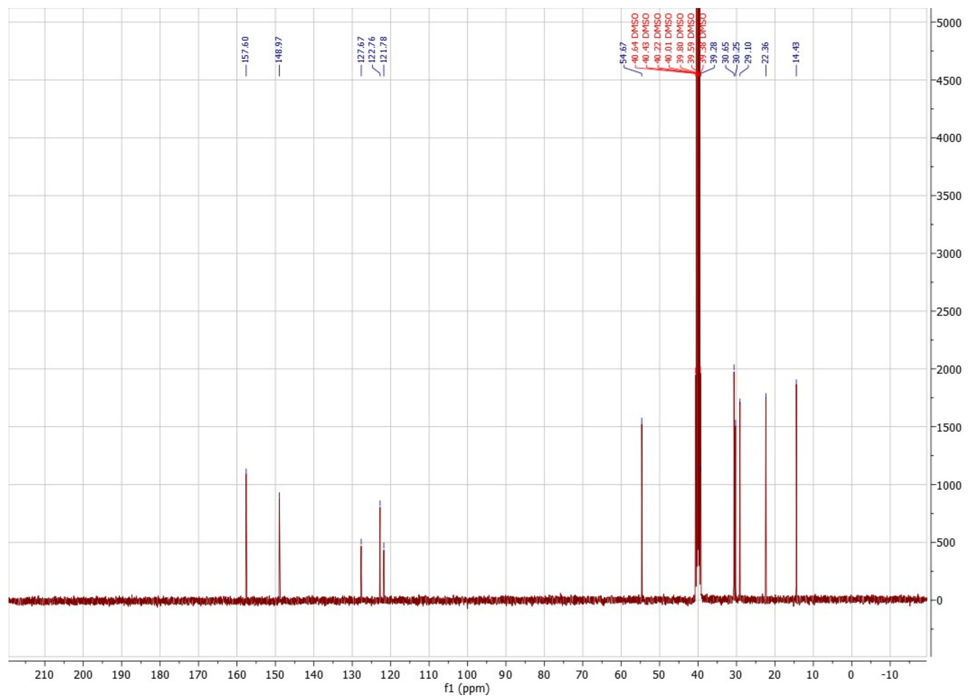

Figure S19 $^{13}C\{^{1}H\}$ NMR spectrum of **1e** in DMSO-d_6.

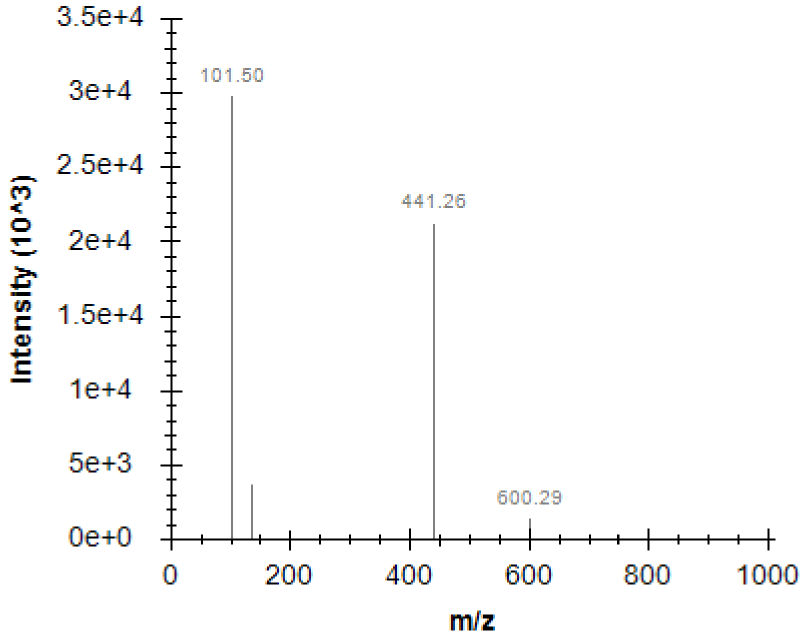

Figure S20 ESI-MS spectrum of **1e** in methanol.

Compound 1f

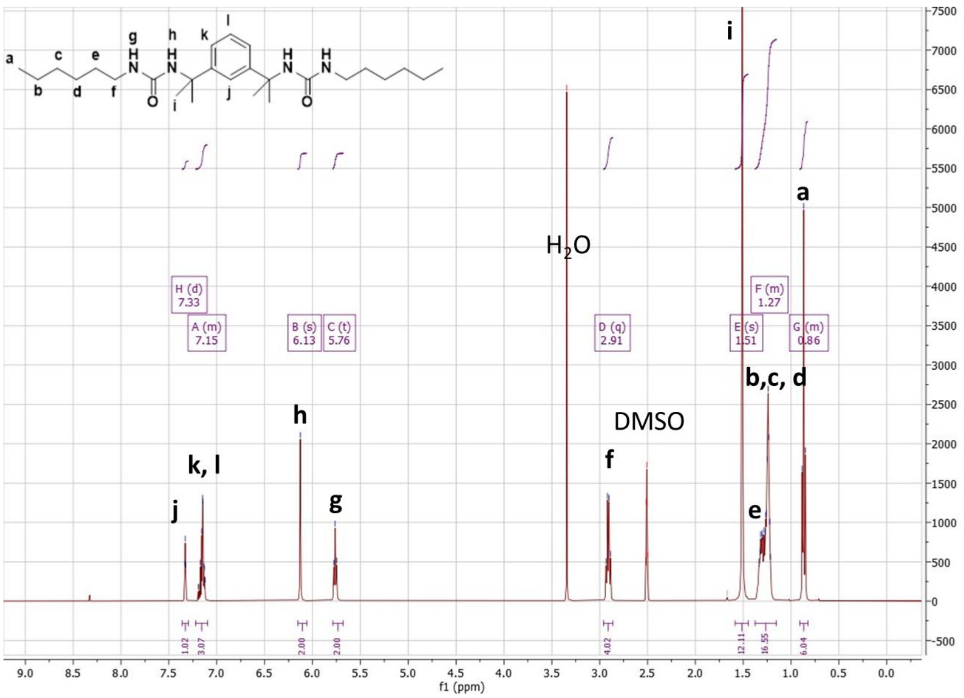

Figure S21 ^{1}H NMR spectrum of **1f** in DMSO-d_6.

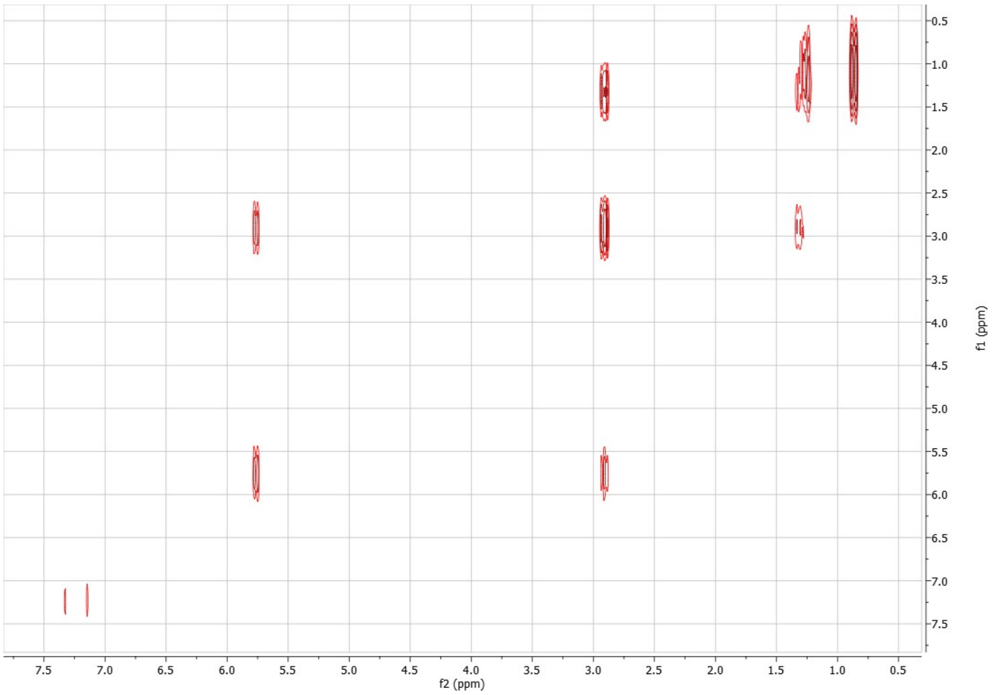

Figure S22 COSY NMR spectrum of **1f** in DMSO-d_6.

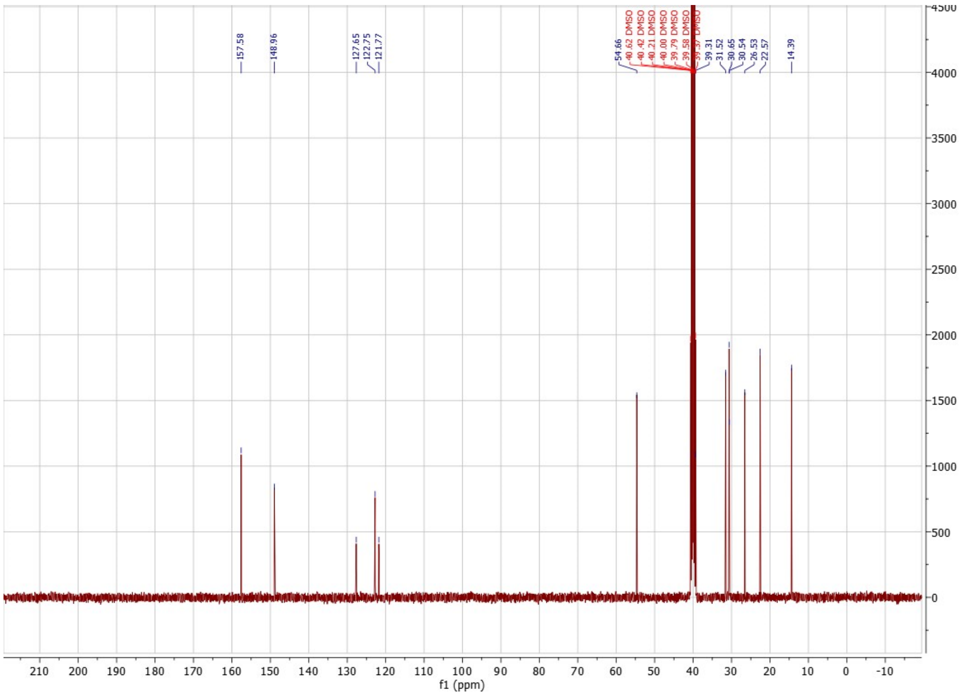

Figure S23 $^{13}C\{^{1}H\}$ NMR spectrum of **1f** in DMSO-d_6.

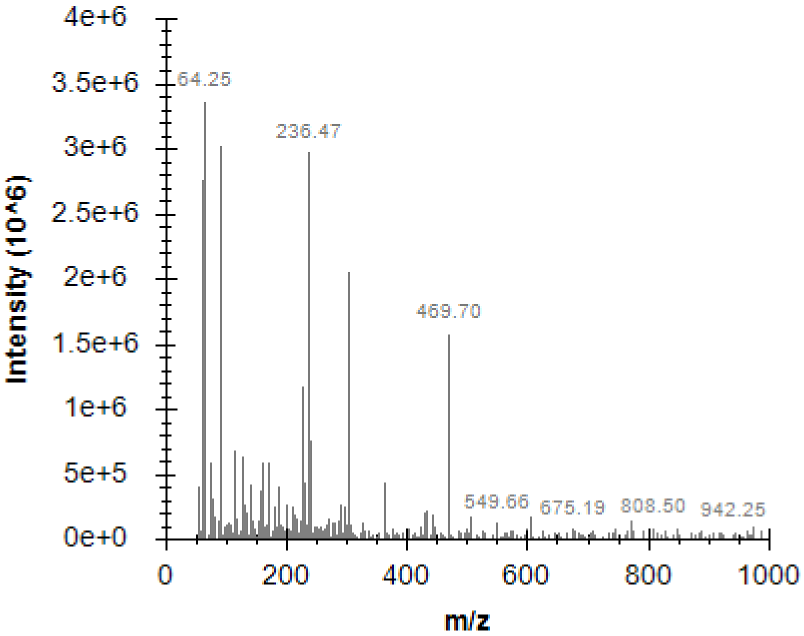

Figure S24 ESI-MS spectrum of **1f** in methanol.

Compound 2a

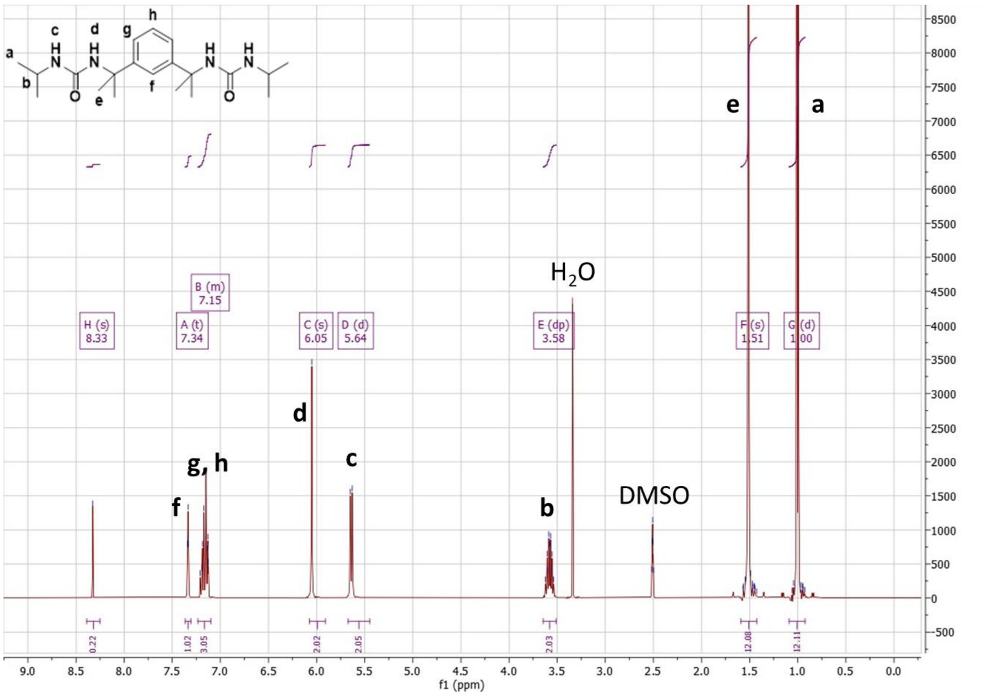

Figure S25 ^{1}H NMR spectrum of **2a** in DMSO-d_6.

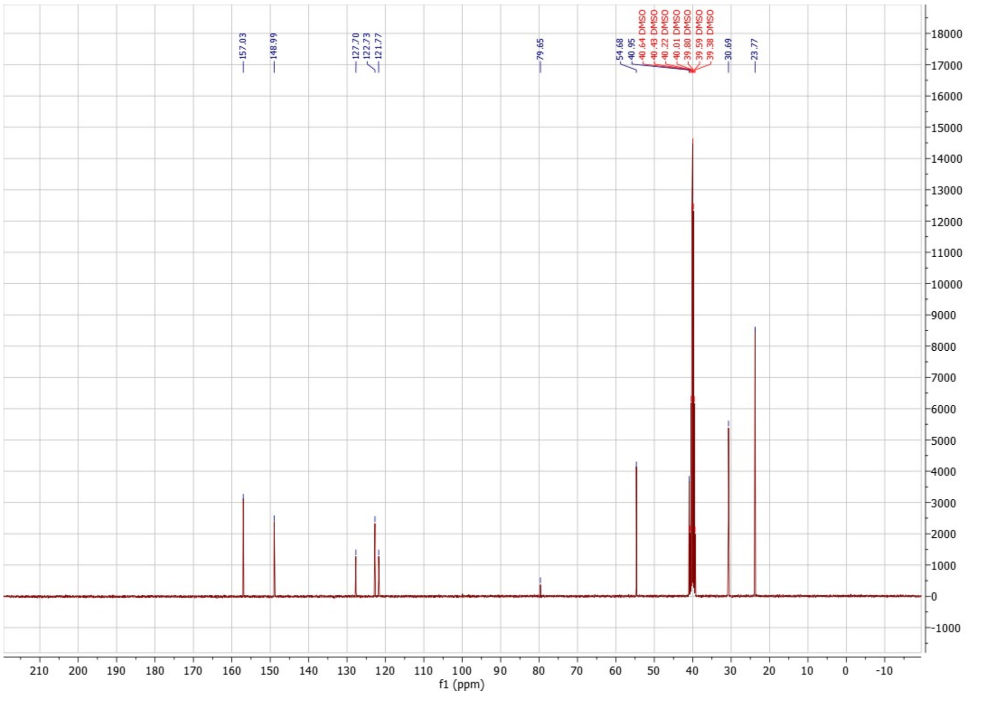

Figure S26 ^{13}C{^{1}H} NMR spectrum of **2a** in DMSO-d_6.

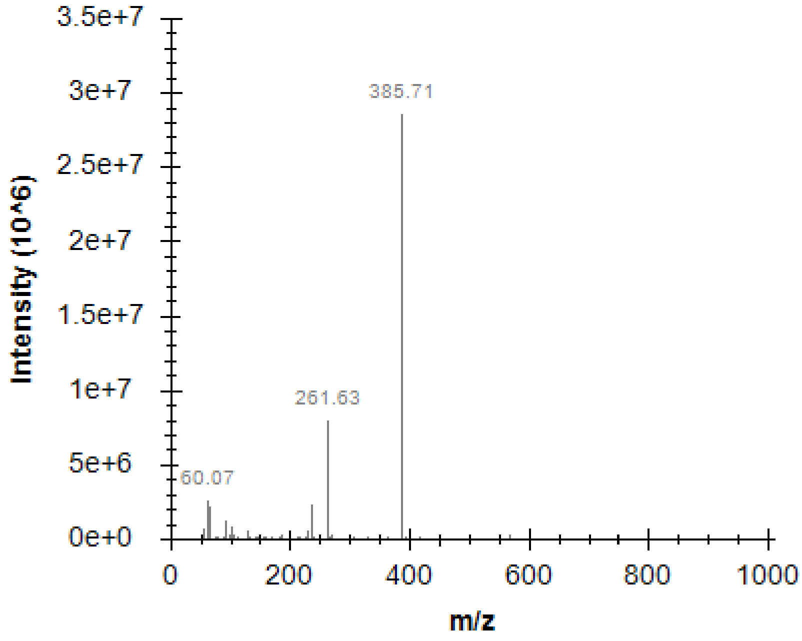

Figure S27 ESI-MS spectrum of **2a** in methanol.

Compound 2b

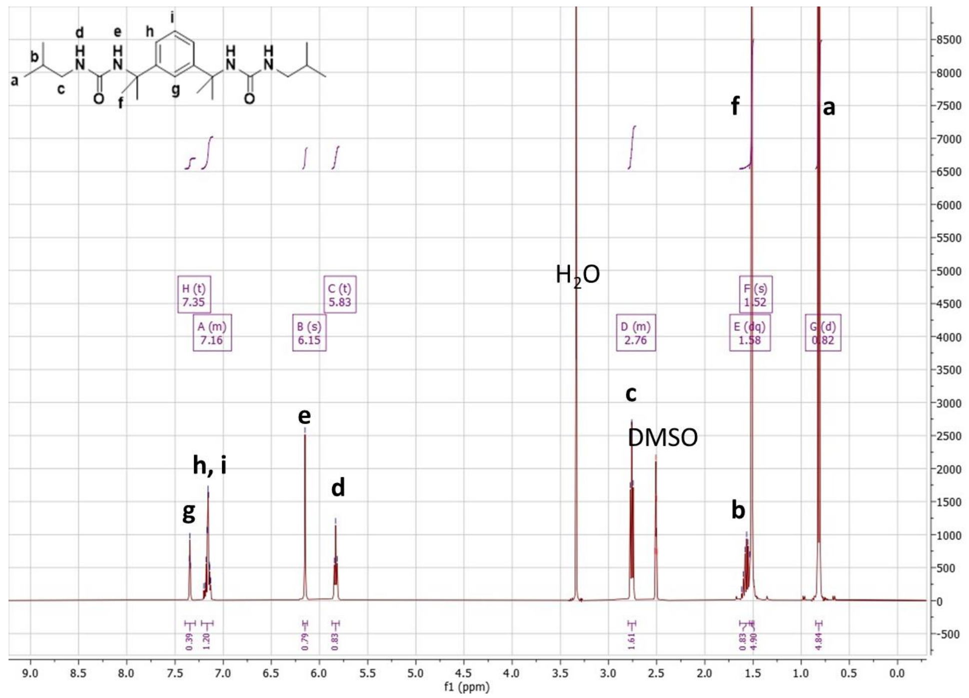

Figure S28 ^{1}H NMR spectrum of **2b** in DMSO-d_6.

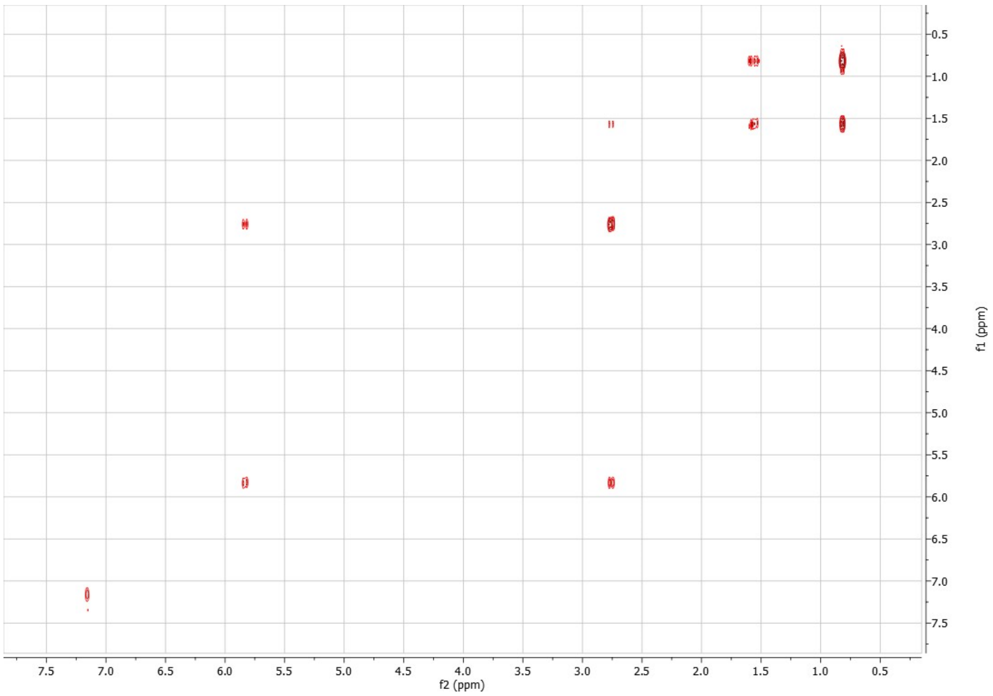

Figure S29 COSY NMR spectrum of **2b** in DMSO-d_6.

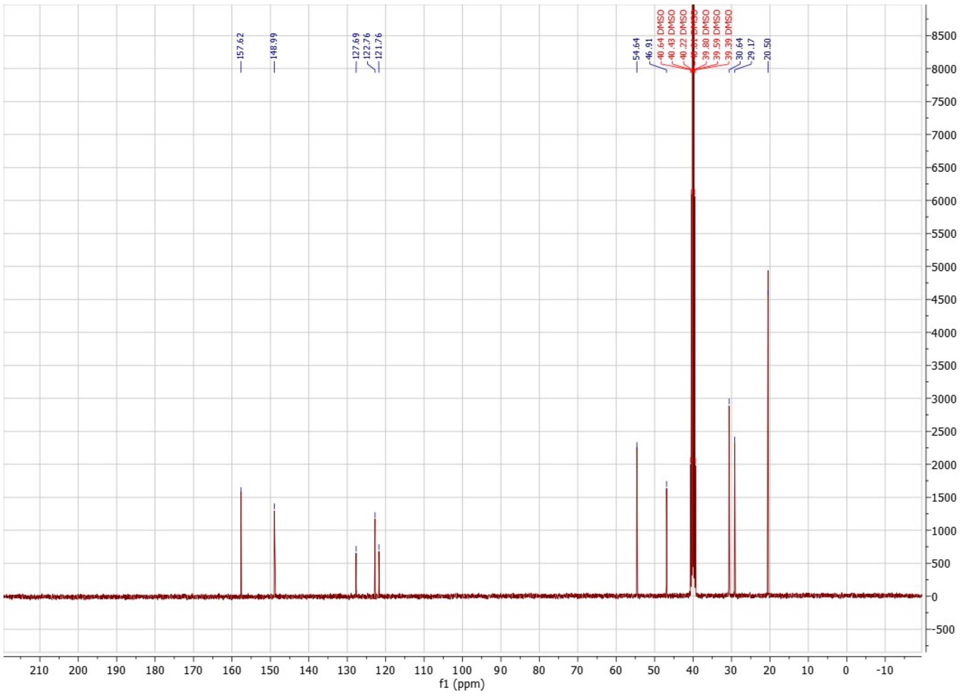

Figure S30 $^{13}C\{^1H\}$ NMR spectrum of **2b** in DMSO-d_6.

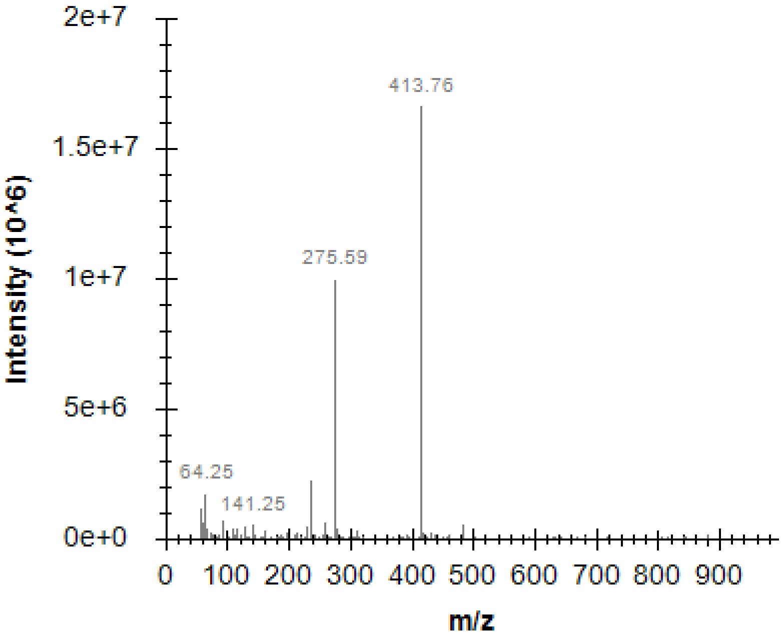

Figure S31 ESI-MS spectrum of **2b** in methanol.

Compound 2c

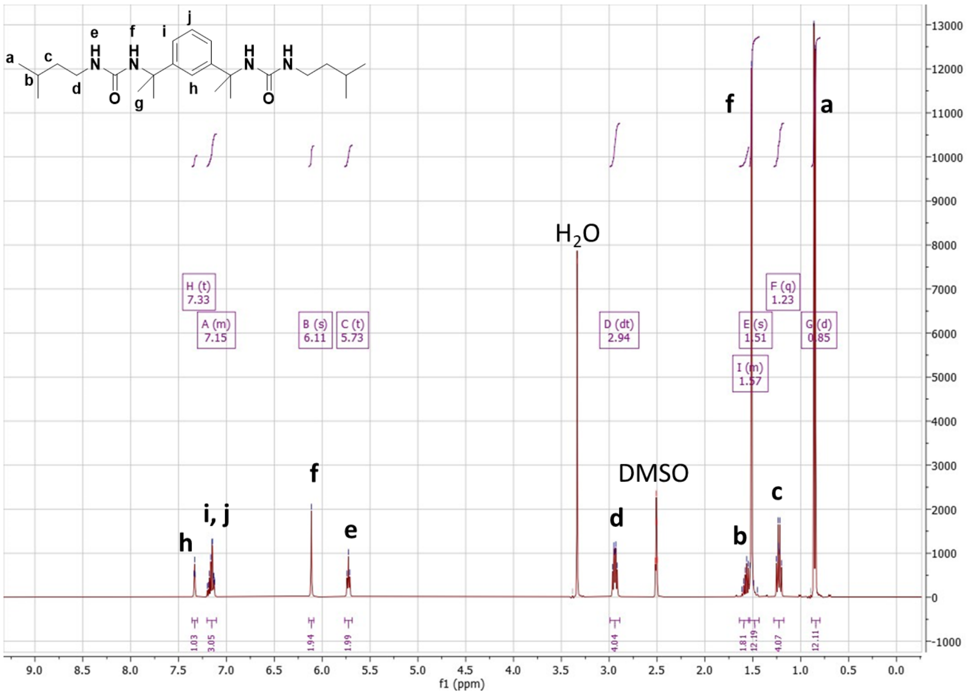

Figure S32 ^{1}H NMR spectrum of **2c** in DMSO-d_6.

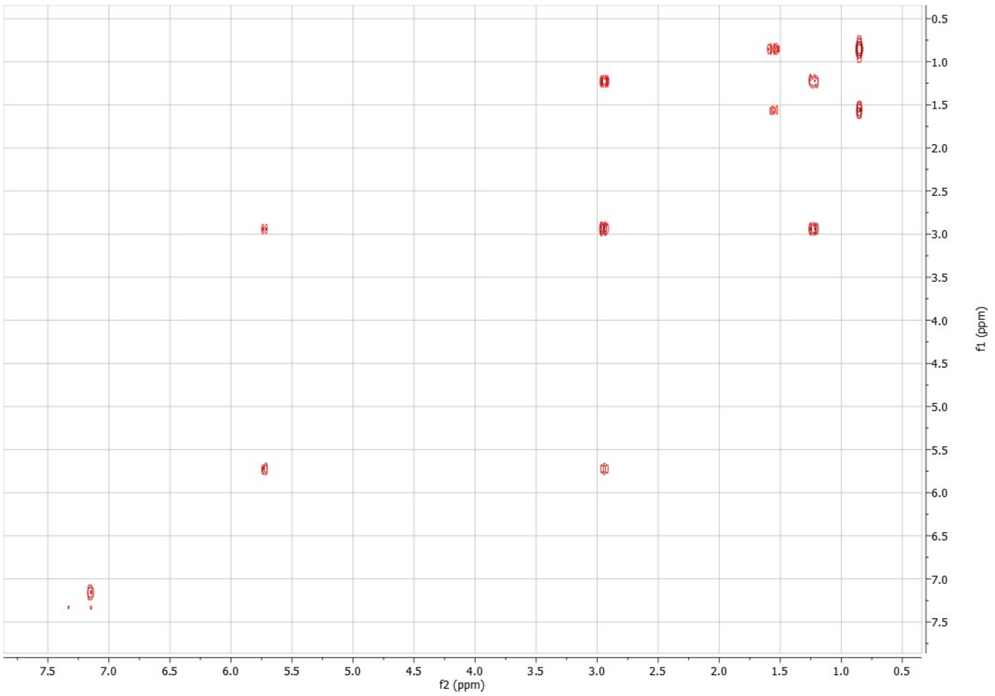

Figure S33 COSY NMR spectrum of **2c** in DMSO-d_6.

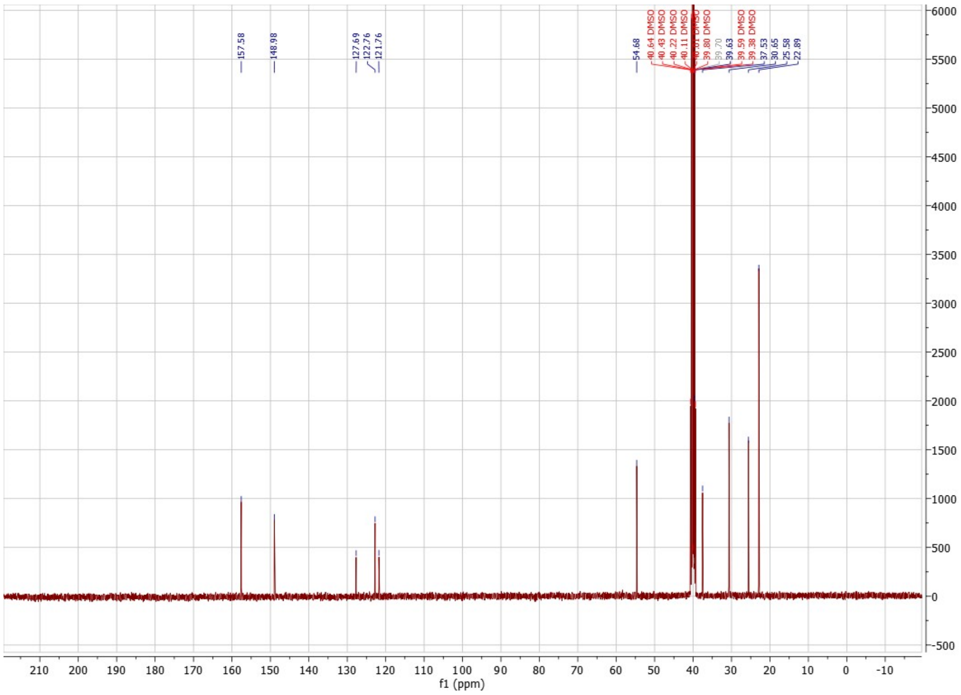

Figure S34 $^{13}C\{^1H\}$ NMR spectrum of **2c** in DMSO-d_6.

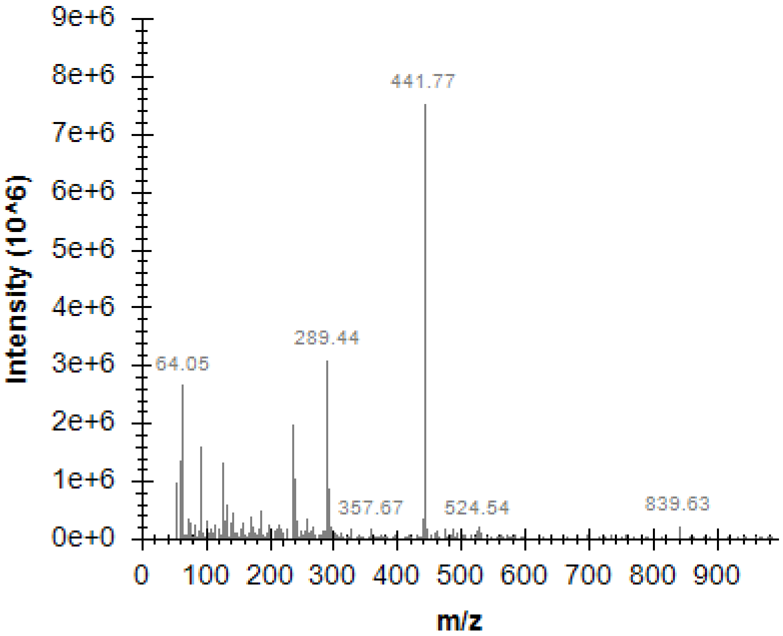

Figure S35 ESI-MS spectrum of **2c** in methanol.

Compound 3

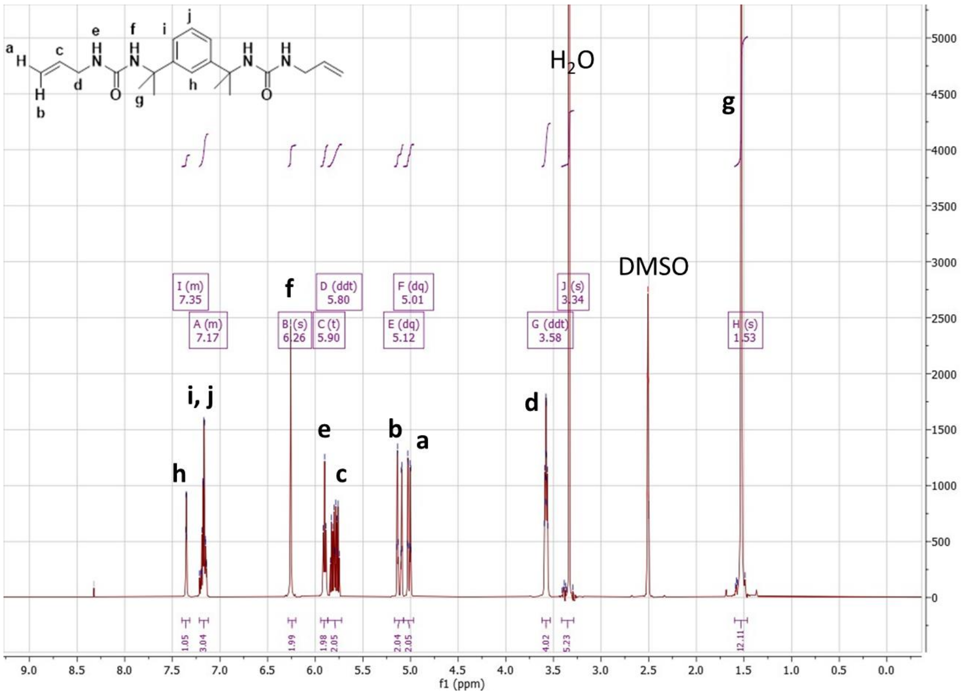

Figure S36 ^{1}H NMR spectrum of **3** in DMSO-d_6.

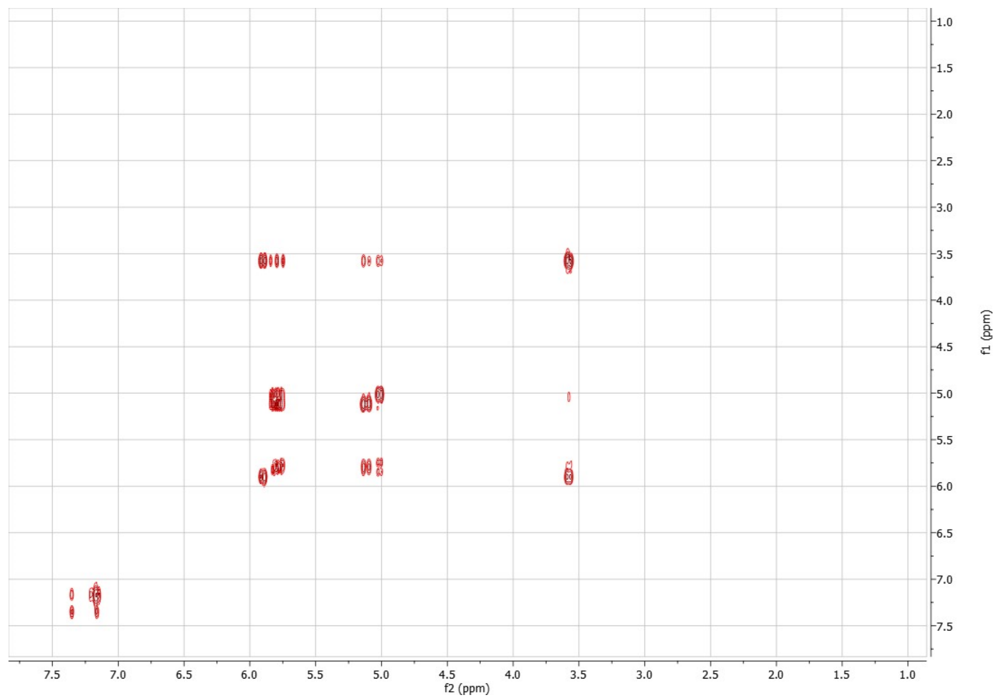

Figure S37 COSY NMR spectrum of **3** in DMSO-d_6.

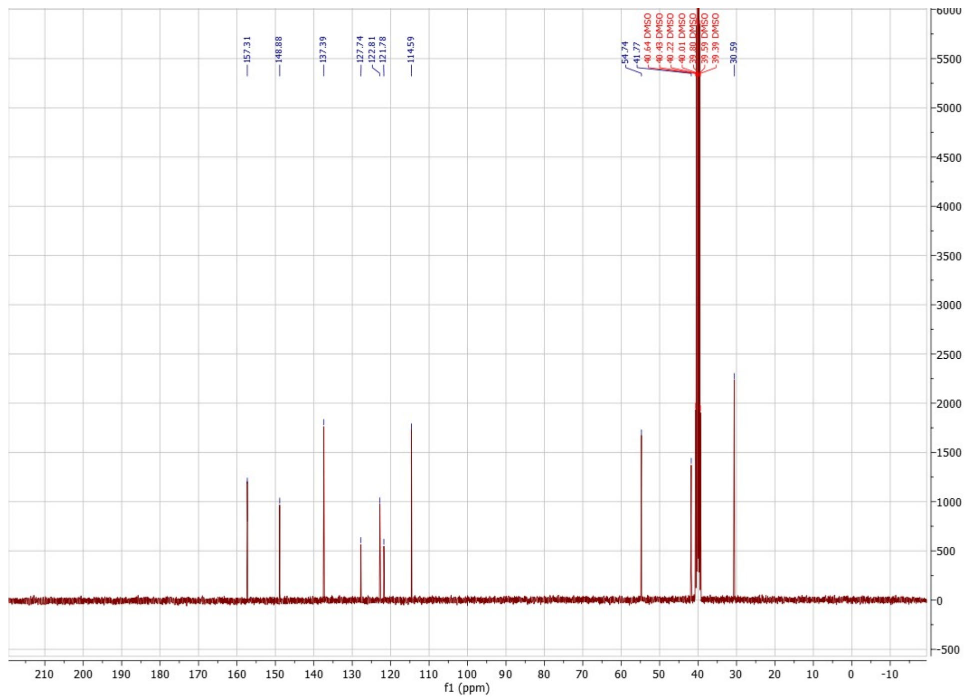

Figure S38 $^{13}C\{^1H\}$ NMR spectrum of **3** in DMSO-d_6.

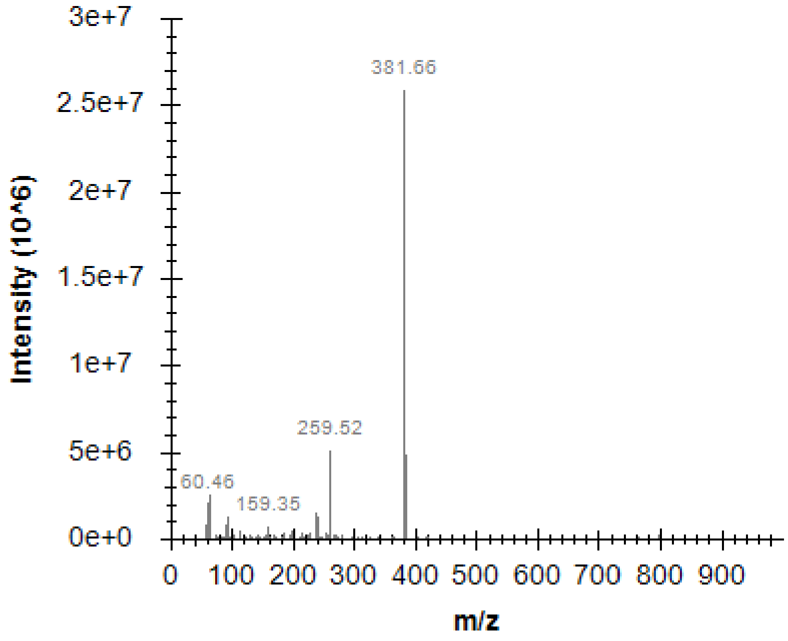

Figure S39 ESI-MS spectrum of **3** in methanol.

Compound 4

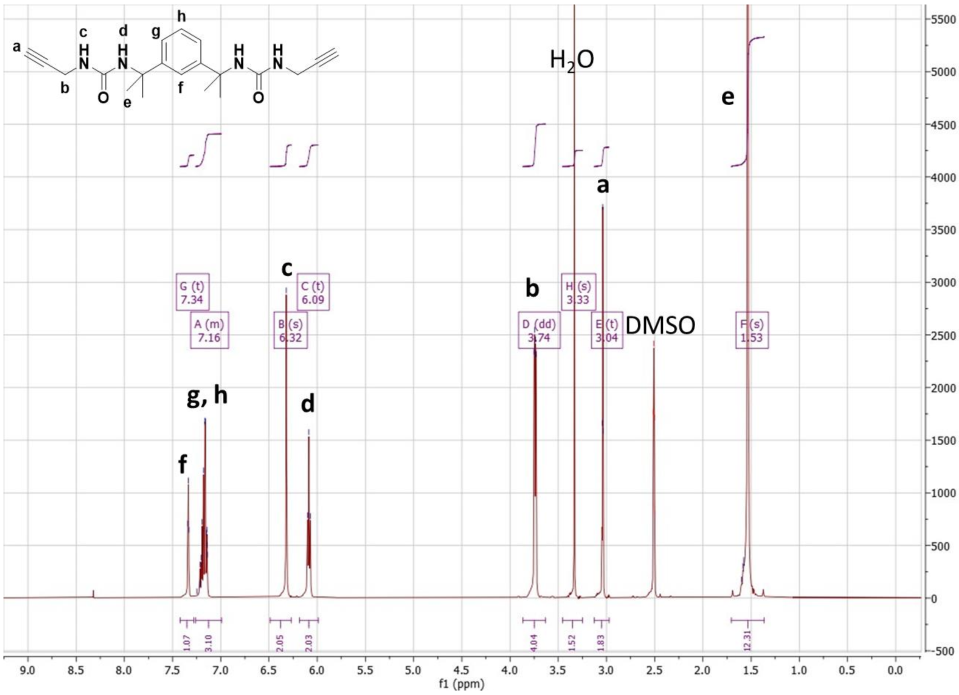

Figure S40 ^{1}H NMR spectrum of **4** in DMSO-d_6.

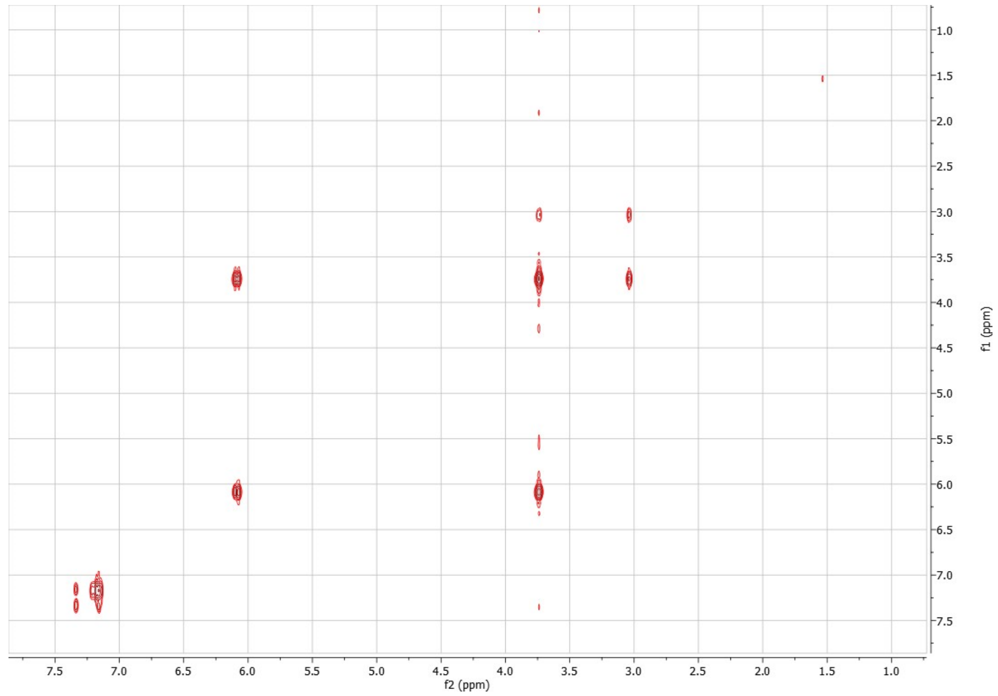

Figure S41 COSY NMR spectrum of **4** in DMSO-d_6.

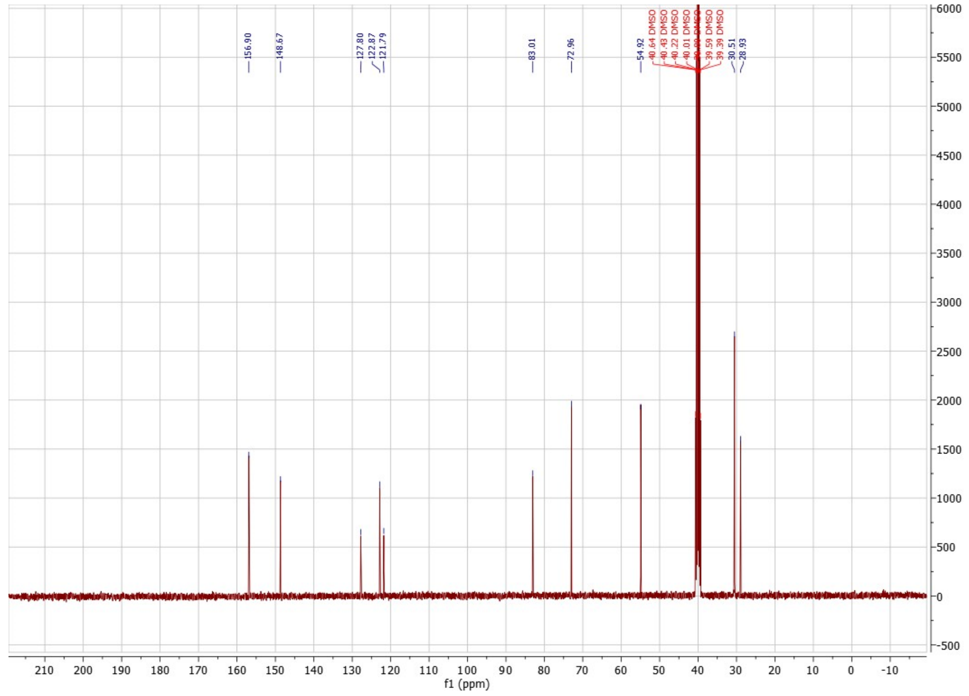

Figure S42 $^{13}C\{^{1}H\}$ NMR spectrum of **4** in DMSO-d_6.

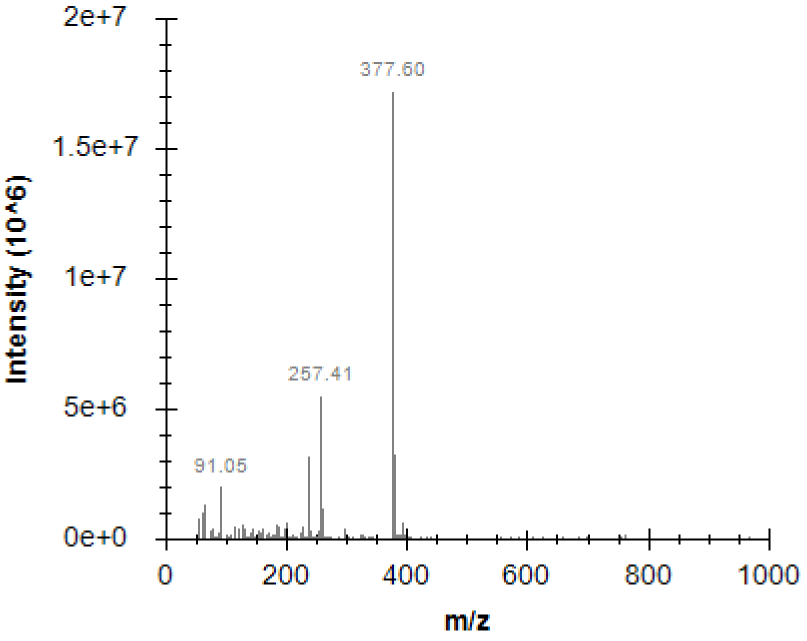

Figure S43 ESI-MS spectrum of **4** in methanol.

Compound 5b

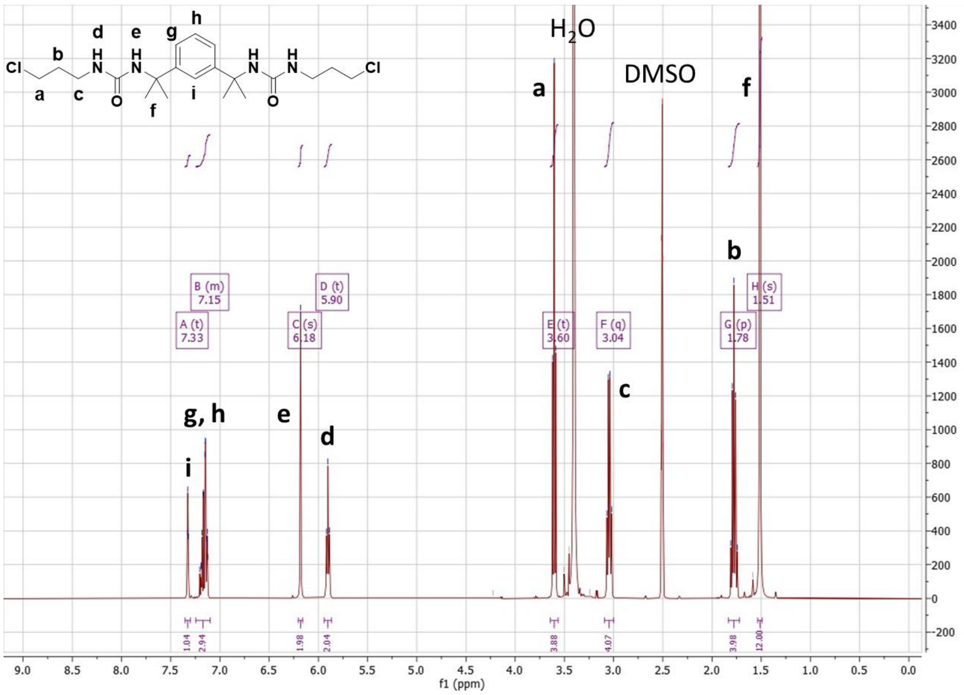

Figure S44 ^{1}H NMR spectrum of **5b** in DMSO-d_6.

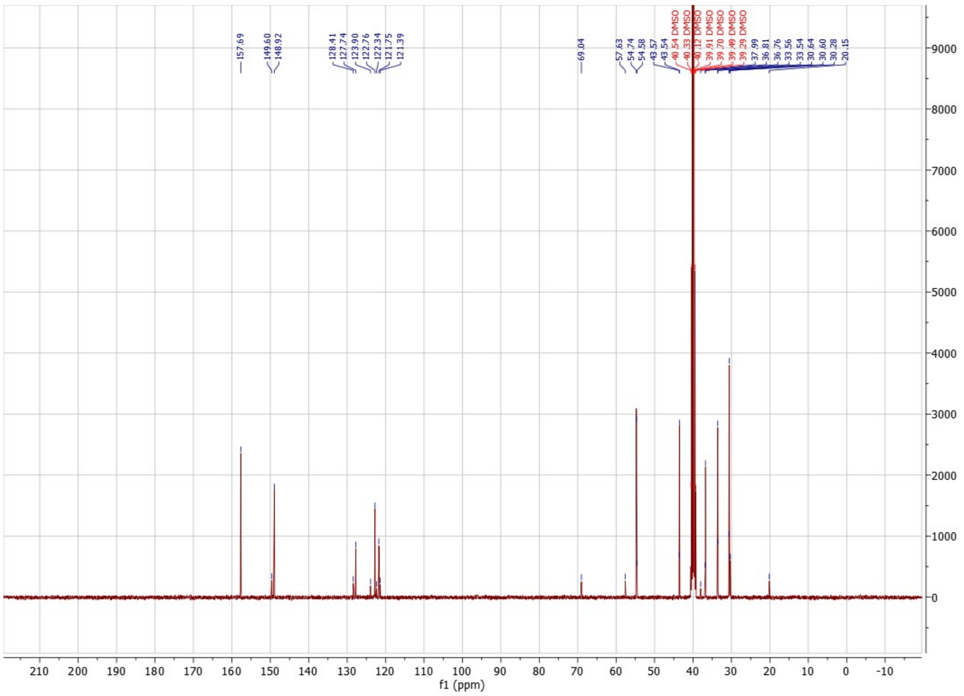

Figure S45 $^{13}C\{^{1}H\}$ NMR spectrum of **5b** in DMSO-d_6.

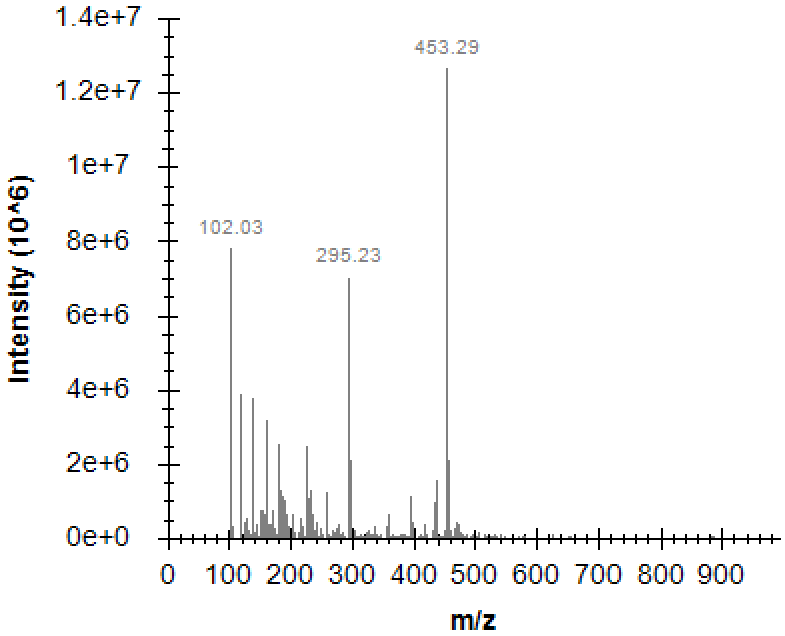

Figure S46 ESI-MS spectrum of **5b** in methanol.

Compound 5c

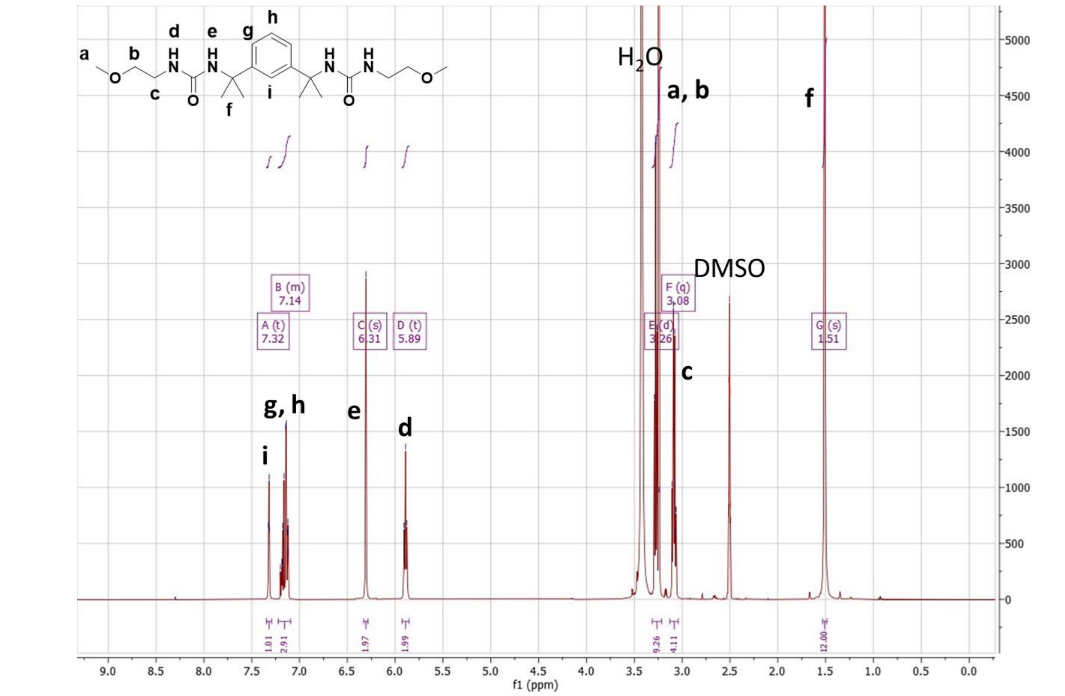

Figure S47 ^{1}H NMR spectrum of **5c** in DMSO-d_6.

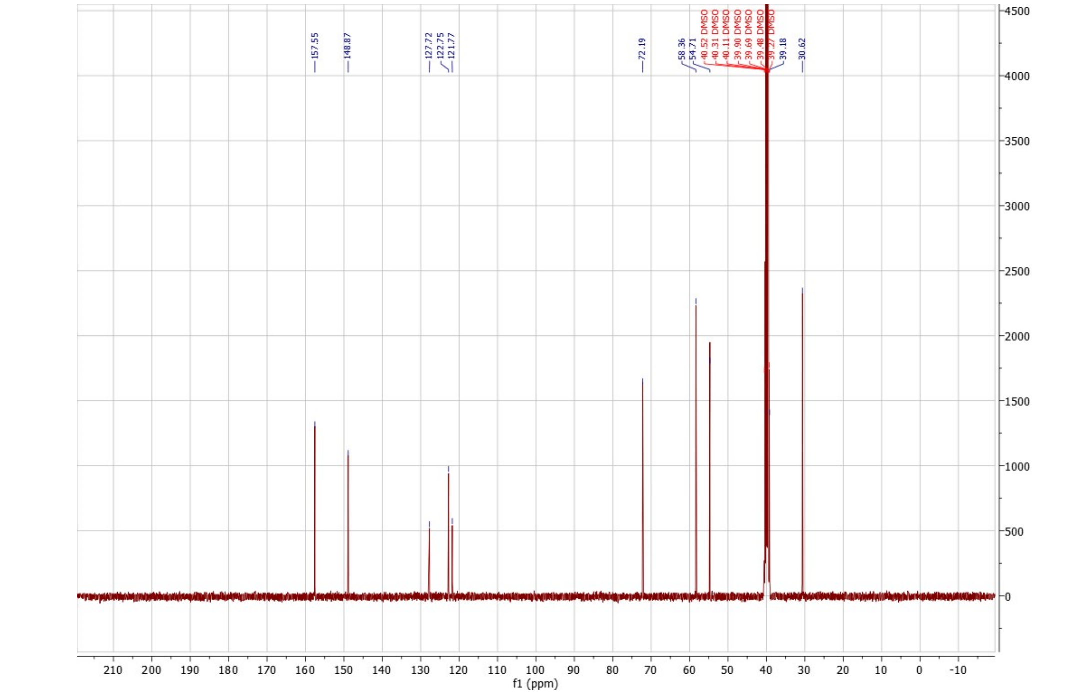

Figure S48 $^{13}C\{^{1}H\}$ NMR spectrum of **5c** in DMSO-d_6.

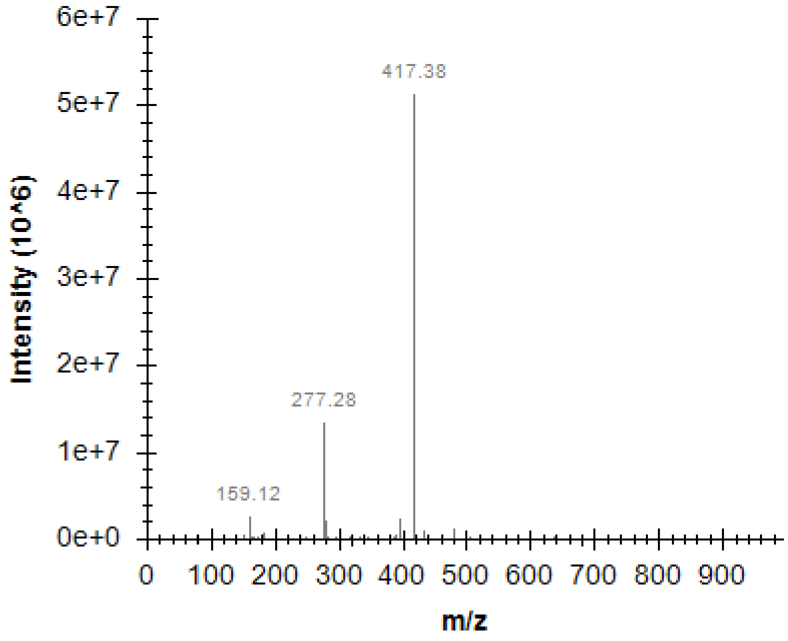

Figure S49 ESI-MS spectrum of **5c** in methanol.

Compound 6

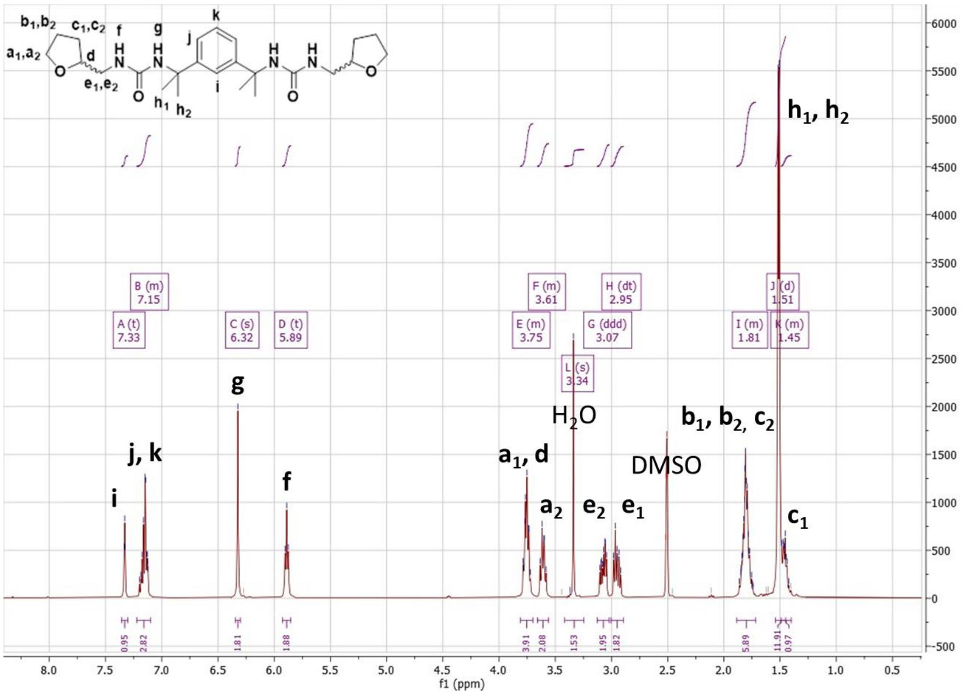

Figure S50 ^{1}H NMR spectrum of **6** in DMSO-d_6.

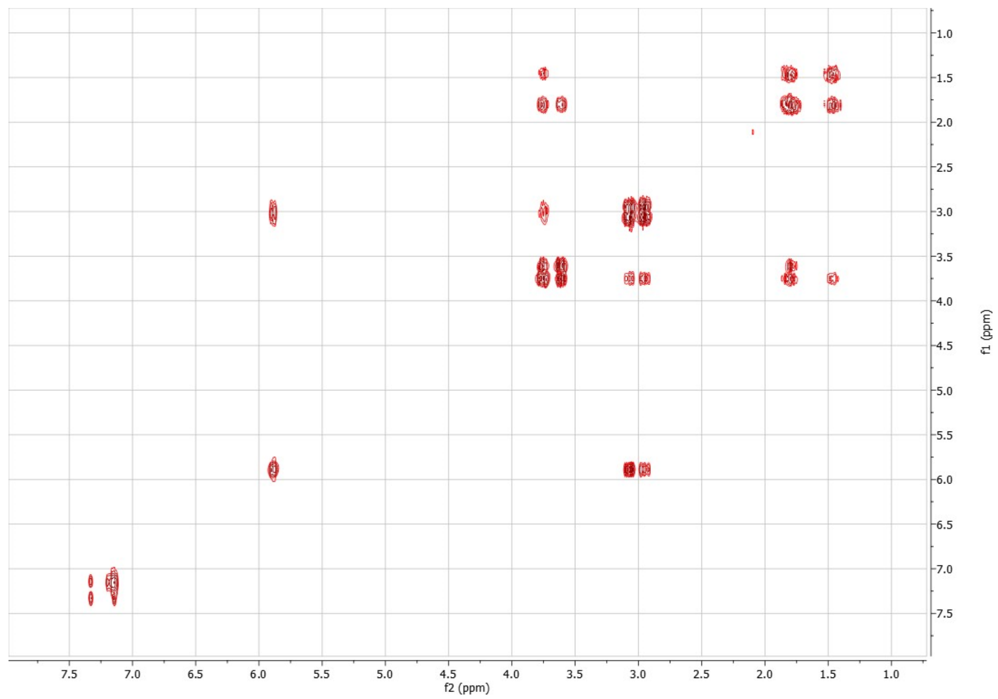

Figure S51 COSY NMR spectrum of **6** in DMSO-d_6.

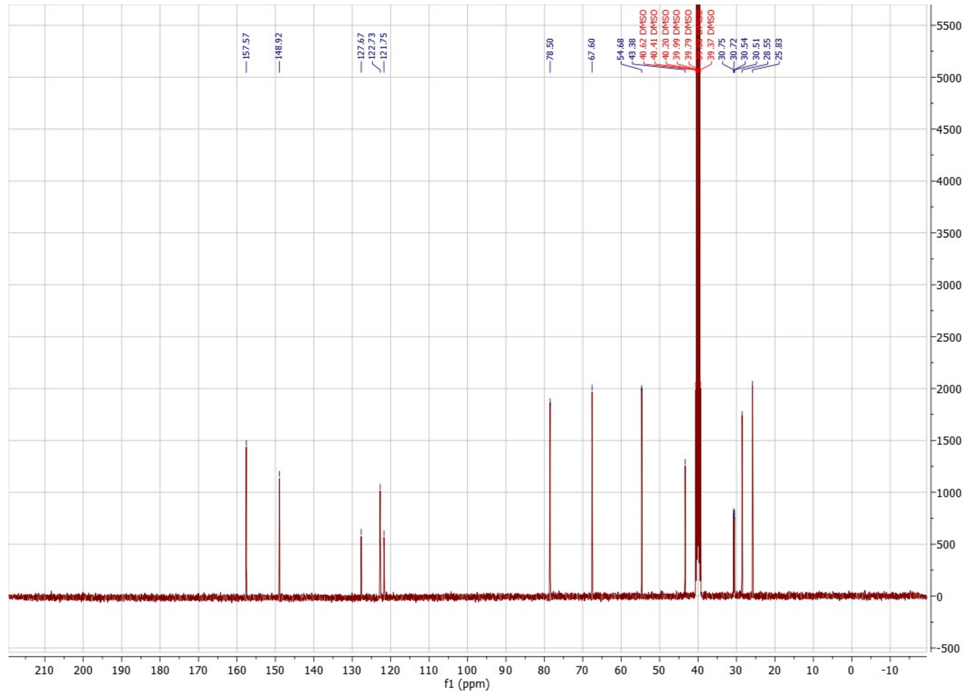

Figure S52 $^{13}C\{^{1}H\}$ NMR spectrum of **6** in DMSO-d_6.

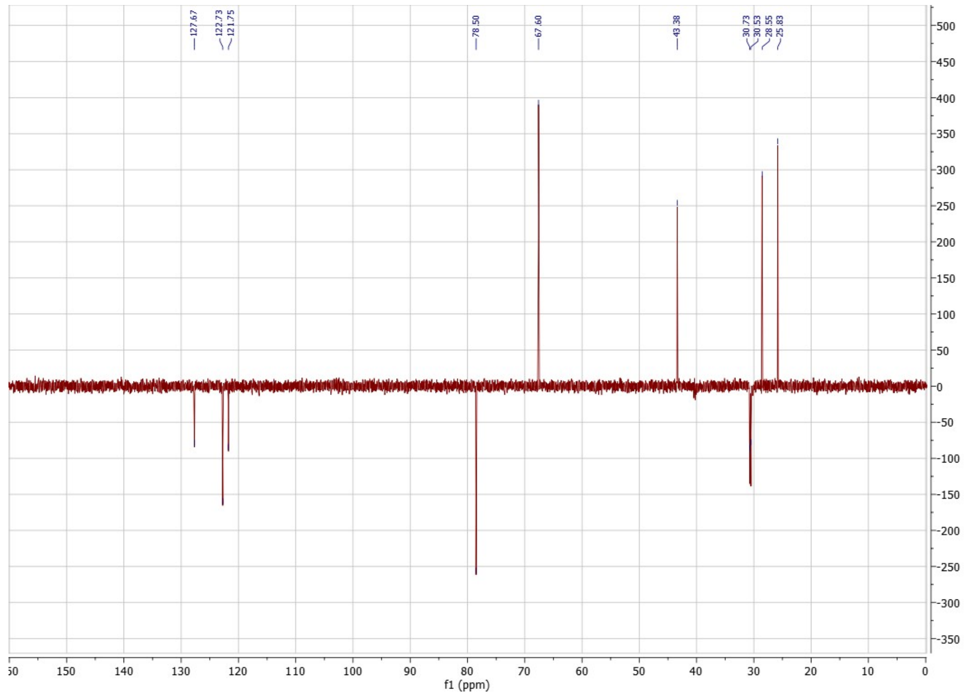

Figure S53 DEPT ^{13}C NMR spectrum of **6** in DMSO-d_6.

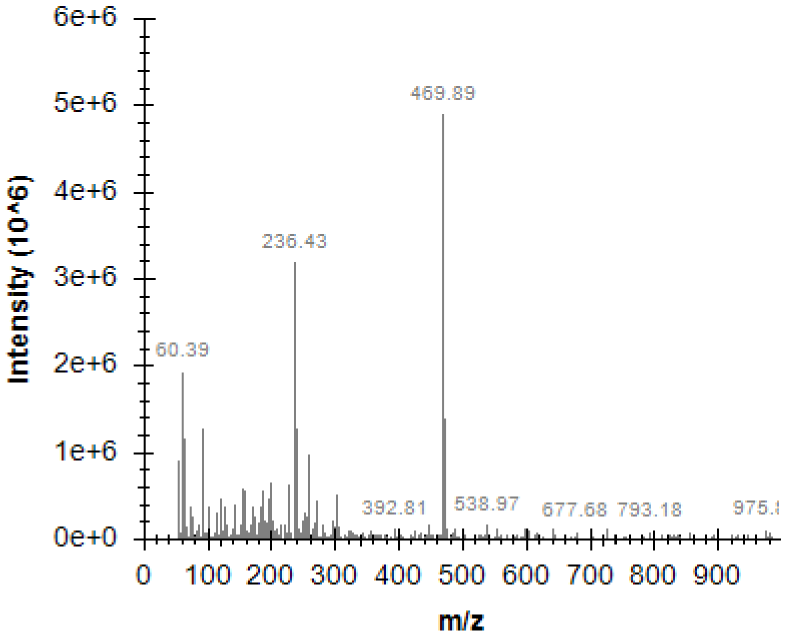

Figure S54 ESI-MS spectrum of **6** in methanol.

Compound 7a

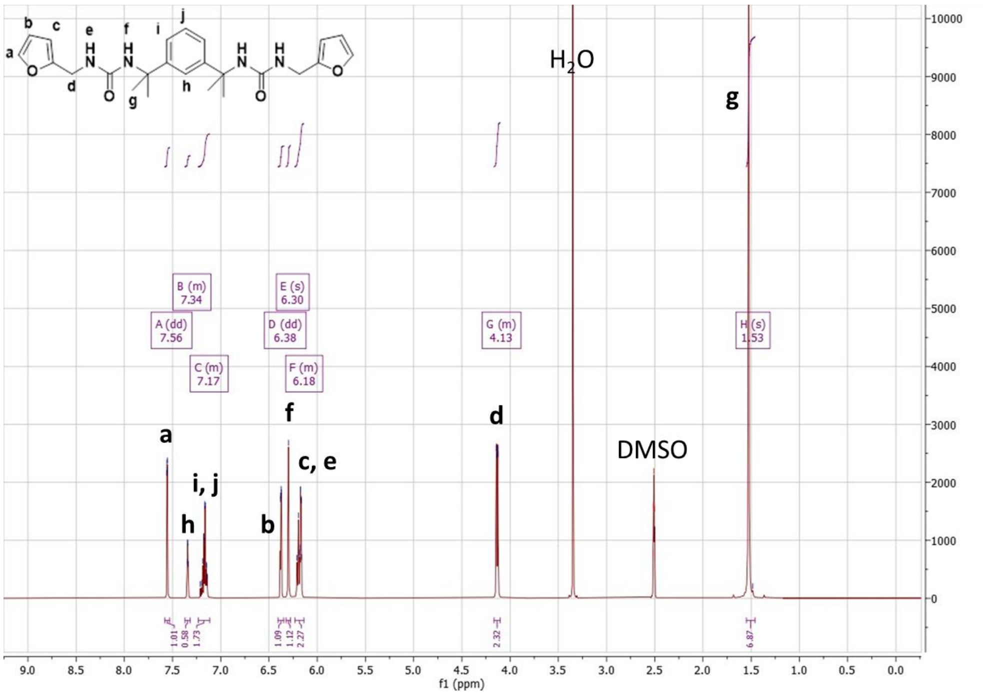

Figure S55 ^{1}H NMR spectrum of **7a** in DMSO-d_6.

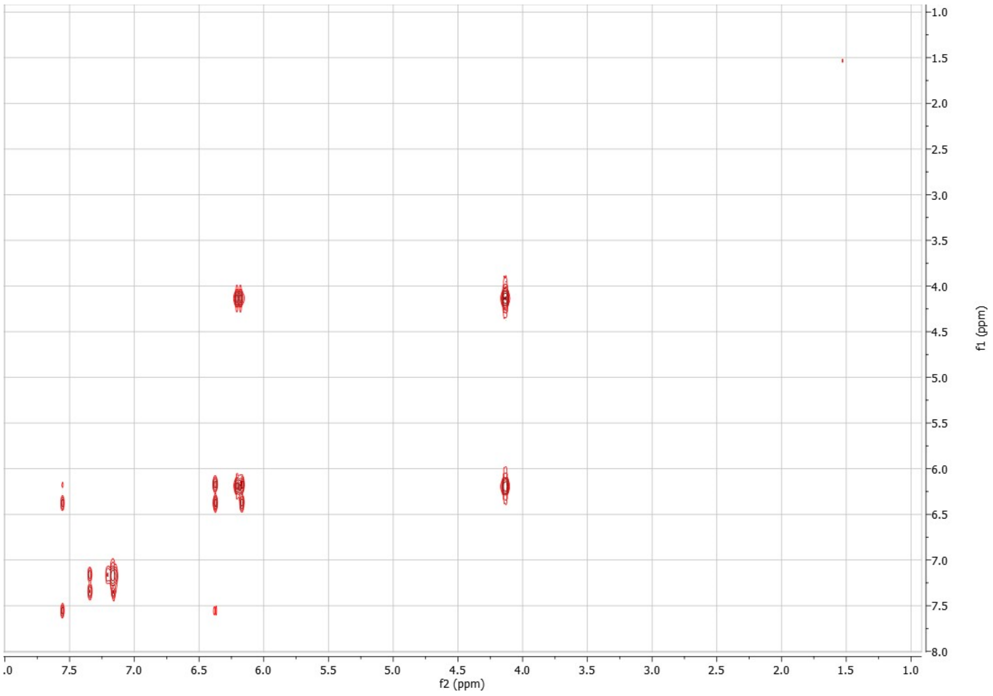

Figure S56 COSY NMR spectrum of **7a** in DMSO-d_6.

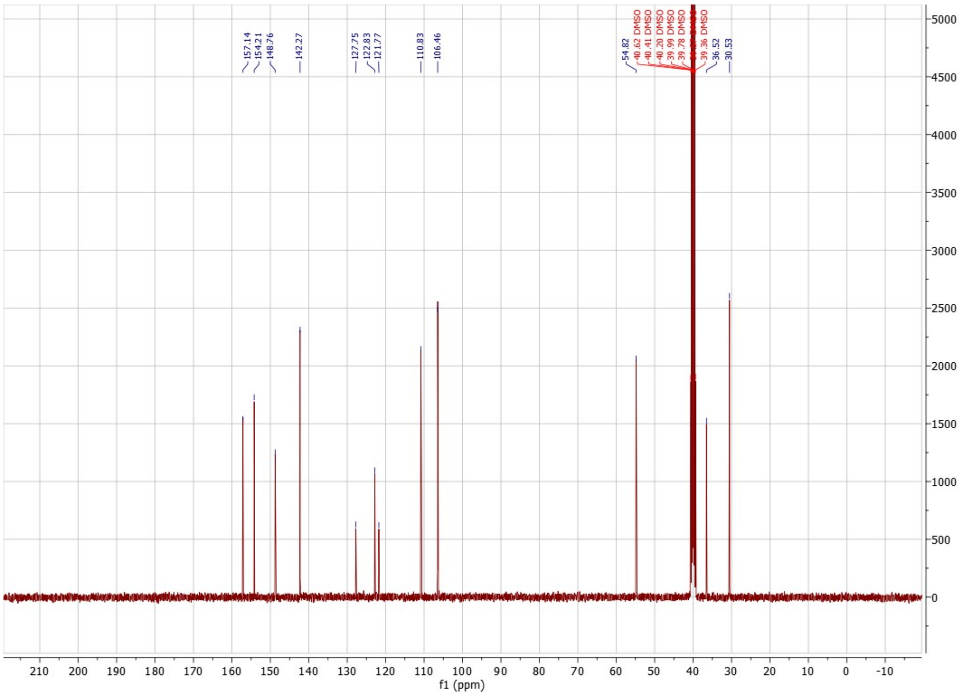

Figure S57 $^{13}C\{^1H\}$ NMR spectrum of **7a** in DMSO-d_6.

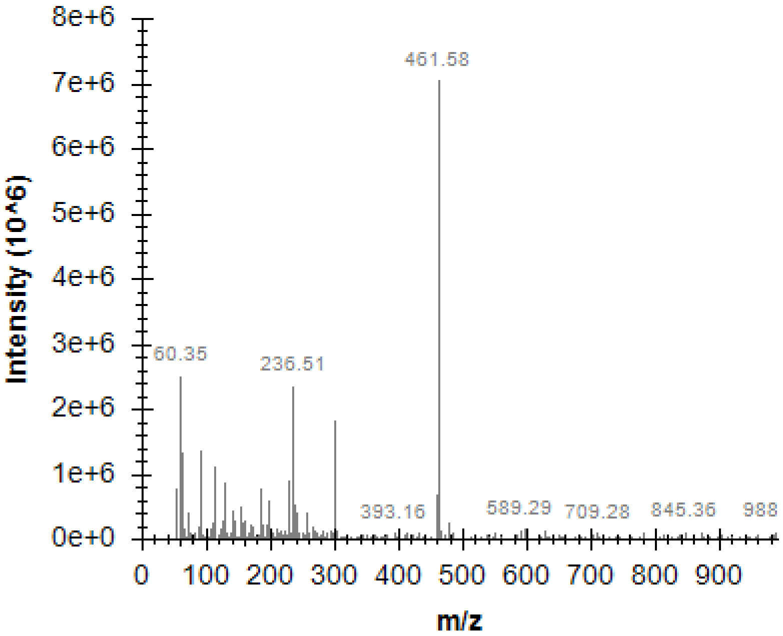

Figure S58 ESI-MS spectrum of **7a** in methanol.

Compound 7b

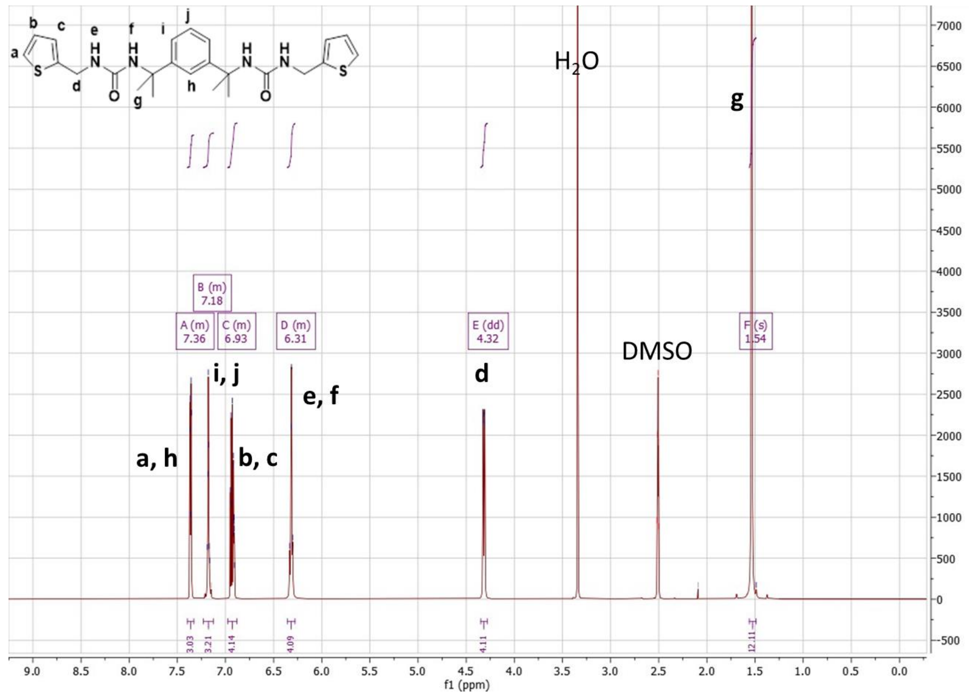

Figure S59 ^{1}H NMR spectrum of **7b** in DMSO-d_6.

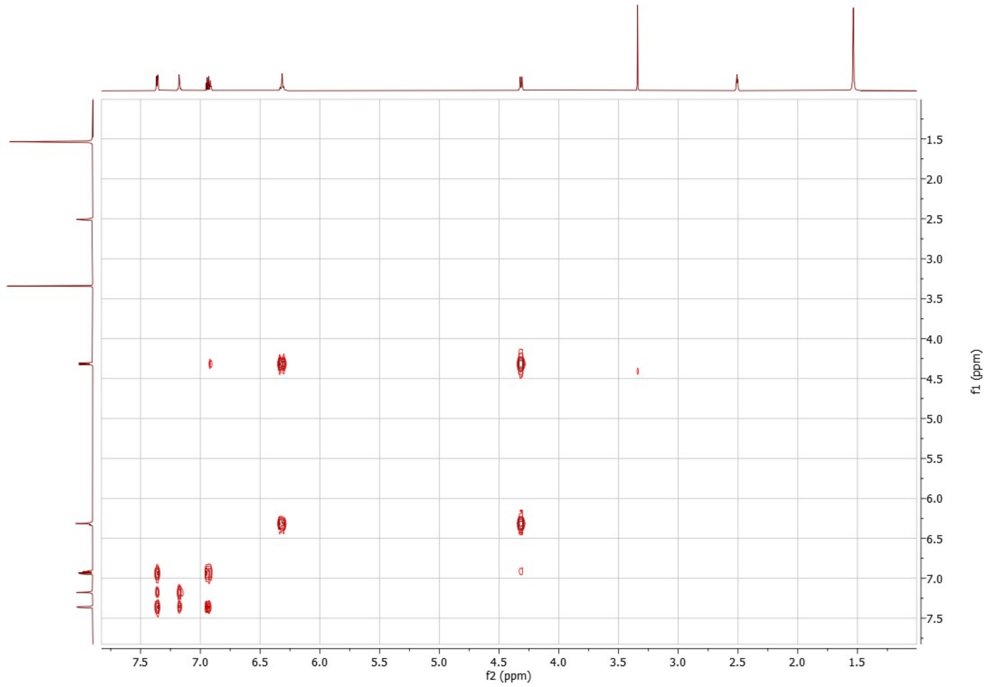

Figure S60 COSY NMR spectrum of **7b** in DMSO-d_6.

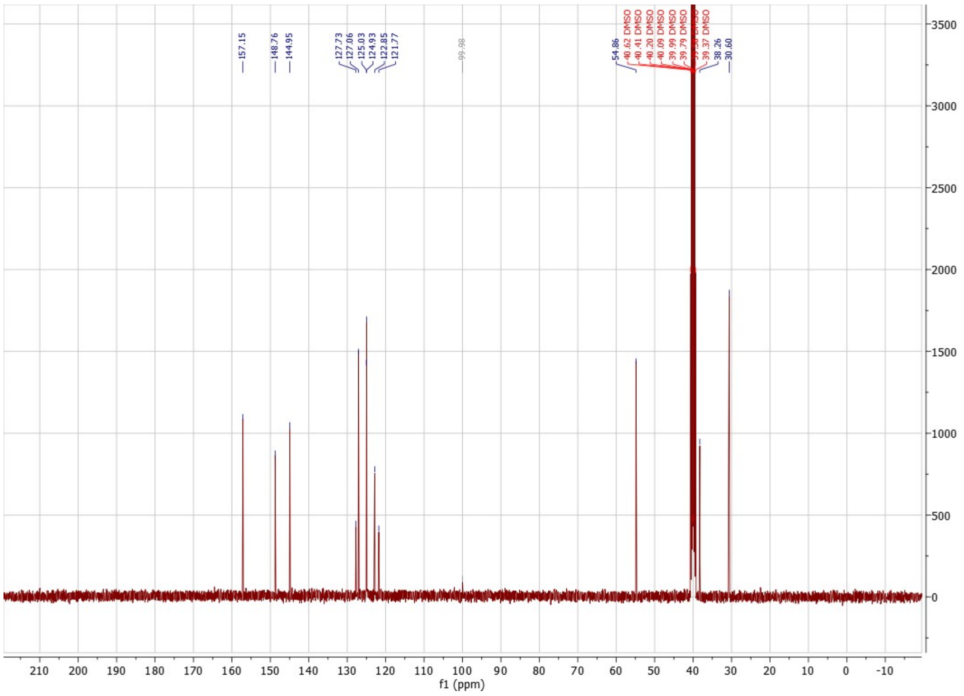

Figure S61 $^{13}C\{^{1}H\}$ NMR spectrum of **7b** in DMSO-d_6.

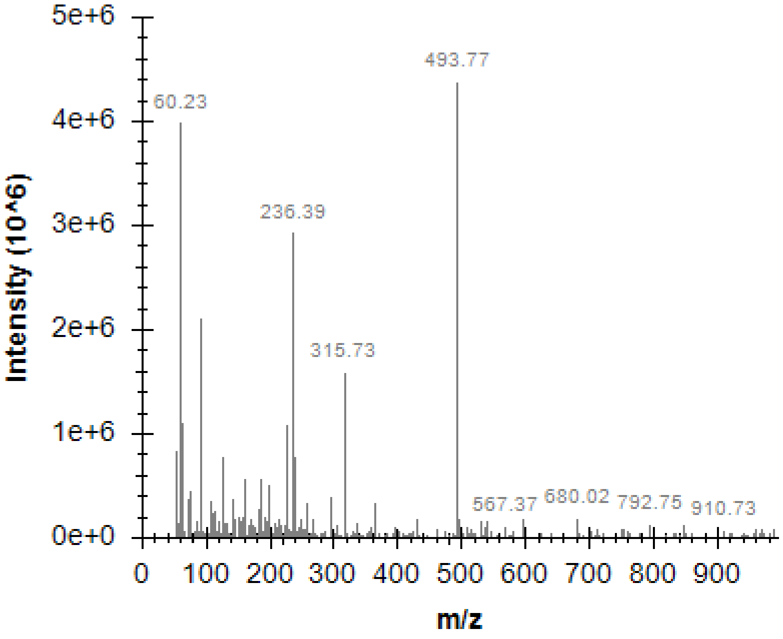

Figure S62 ESI-MS spectrum of **7b** in methanol.

Compound 8

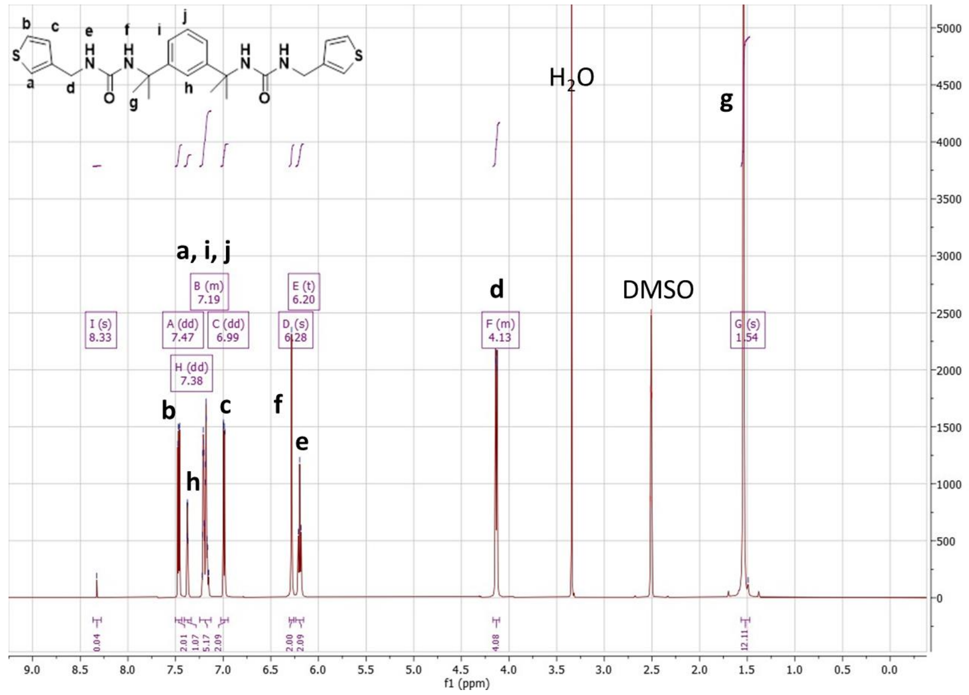

Figure S63 ^{1}H NMR spectrum of **8** in DMSO-d_6.

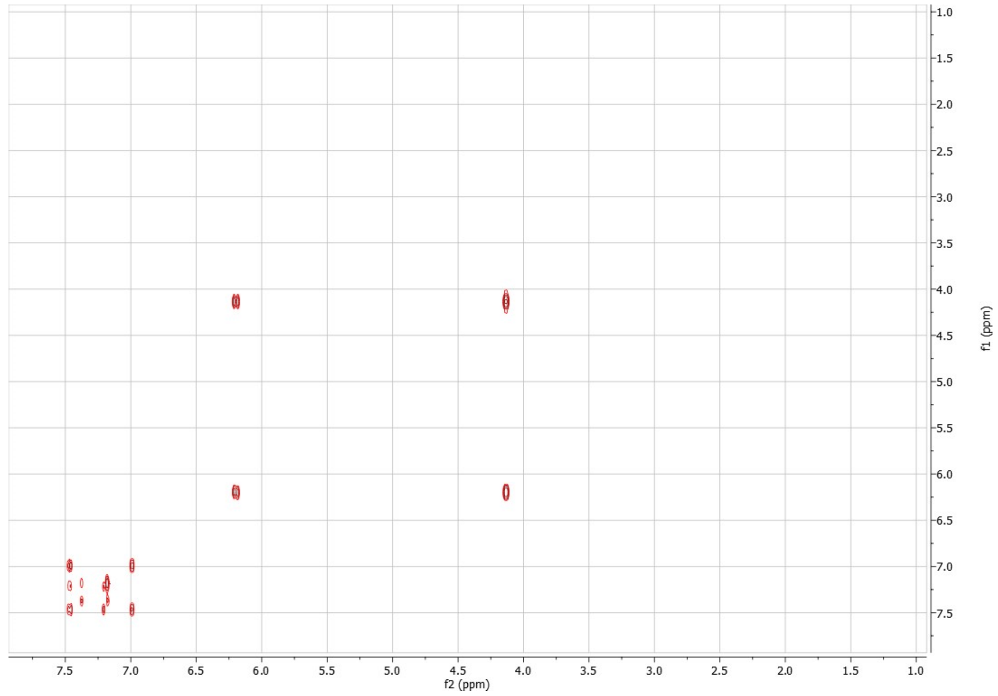

Figure S64 COSY NMR spectrum of **8** in DMSO-d_6.

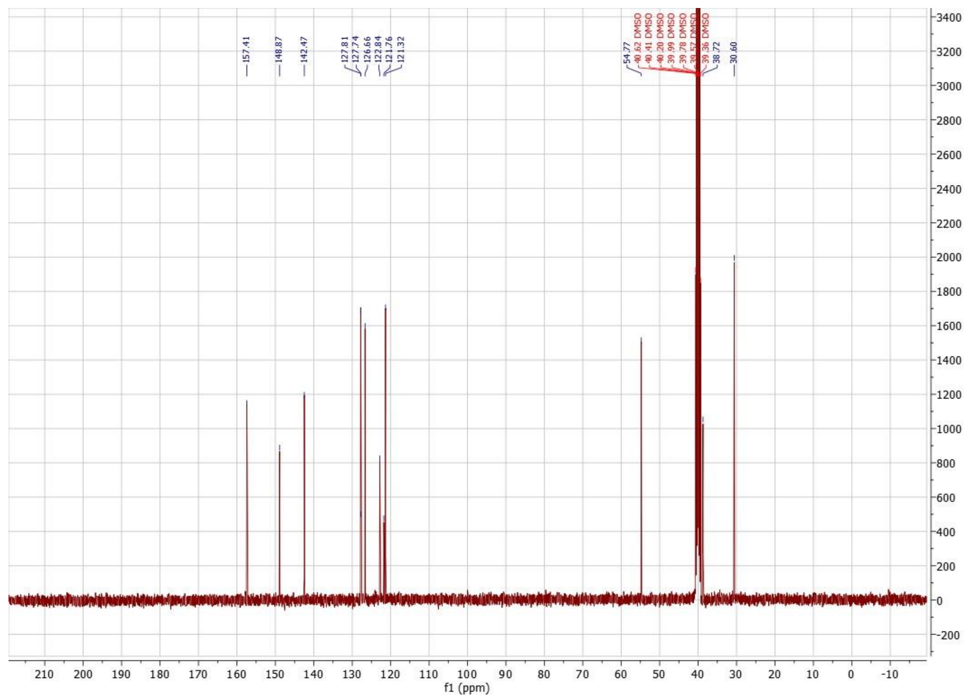

Figure S65 $^{13}C\{^{1}H\}$ NMR spectrum of **8** in DMSO-d_6.

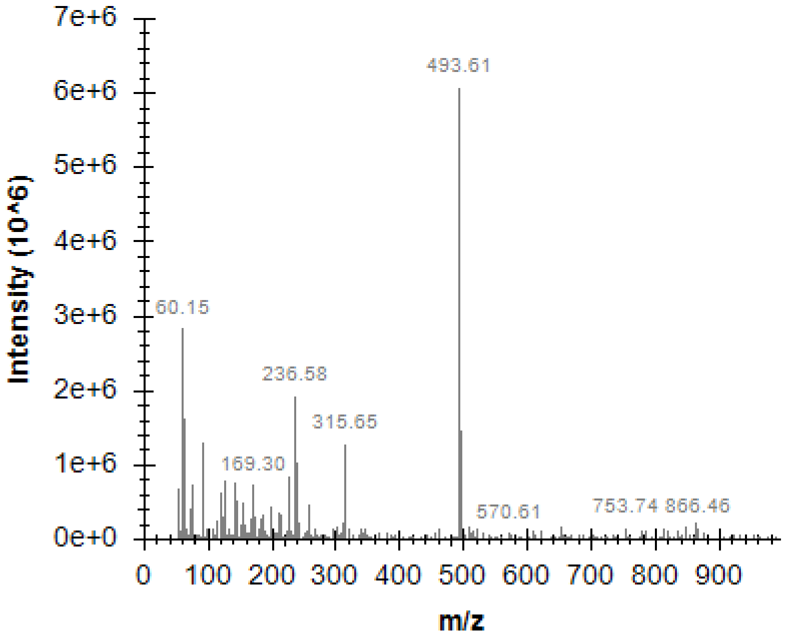

Figure S66 ESI-MS spectrum of **8** in methanol.

Compound 9a

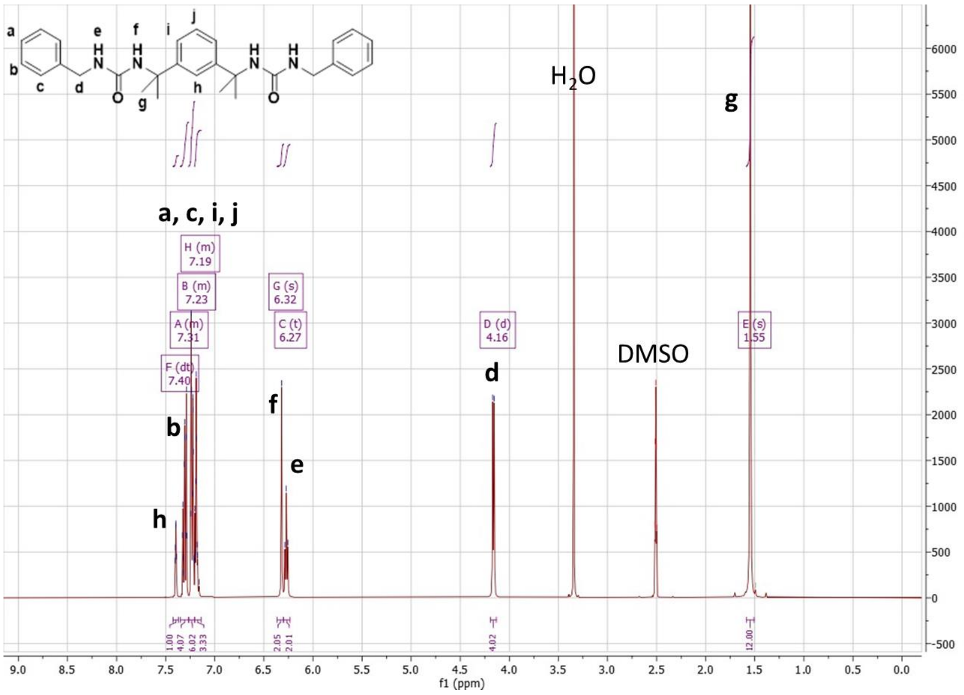

Figure S67 ^{1}H NMR spectrum of **9a** in DMSO-d_6.

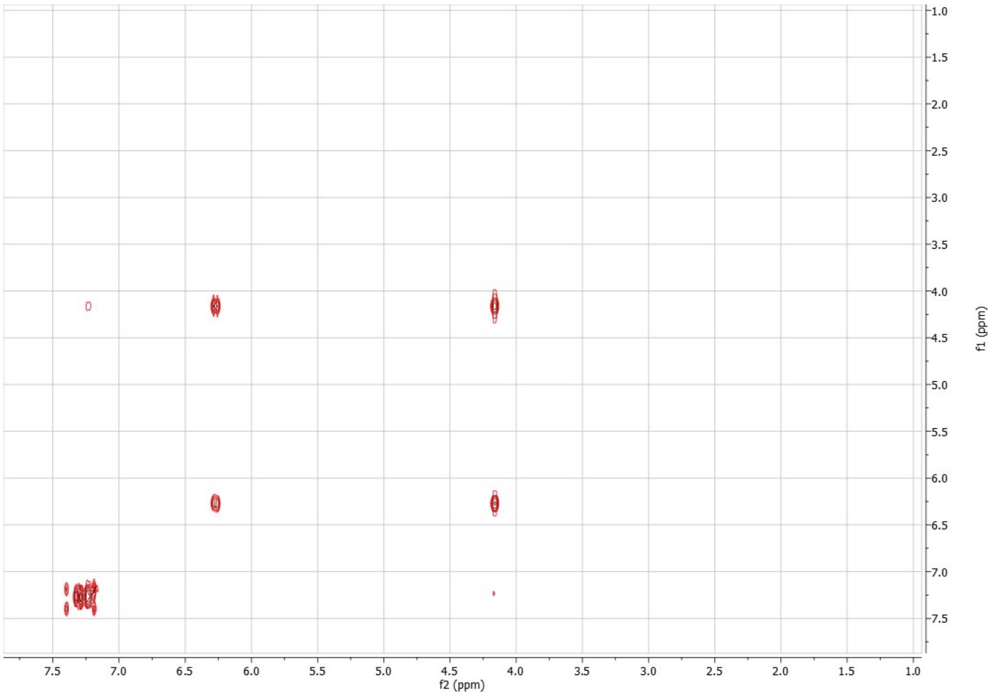

Figure S68 COSY NMR spectrum of **9a** in DMSO-d_6.

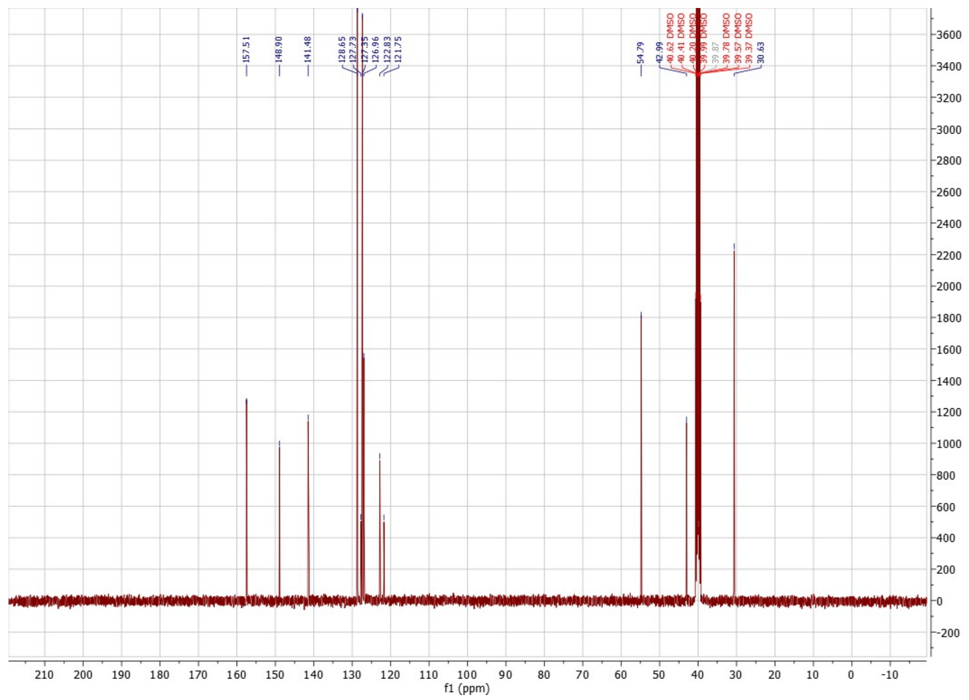

Figure S69 $^{13}C\{^{1}H\}$ NMR spectrum of **9a** in DMSO-d_6.

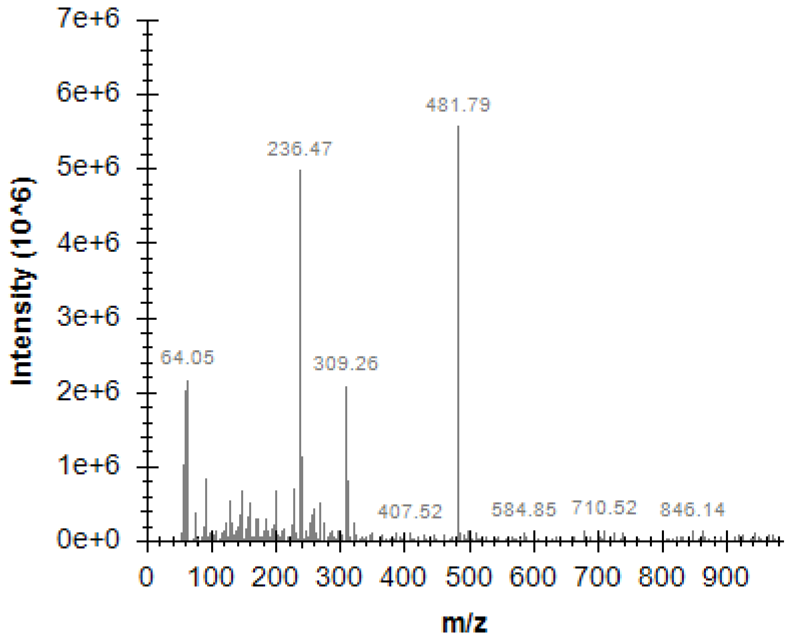

Figure S70 ESI-MS spectrum of **9a** in methanol.

Compound 9b

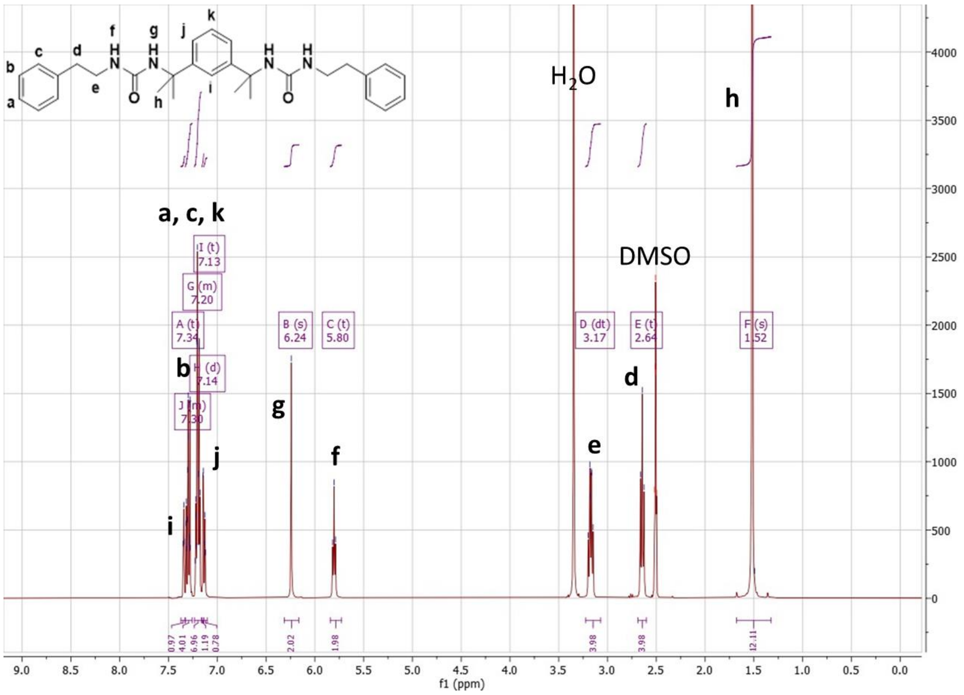

Figure S71 ^{1}H NMR spectrum of **9b** in DMSO-d_6.

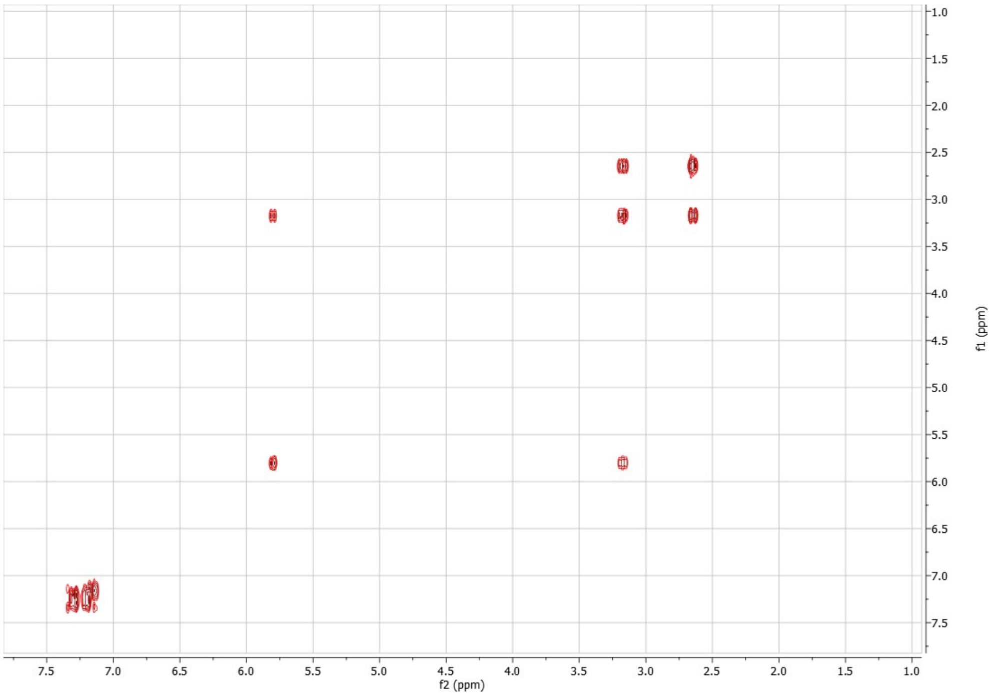

Figure S72 COSY NMR spectrum of **9b** in DMSO-d_6.

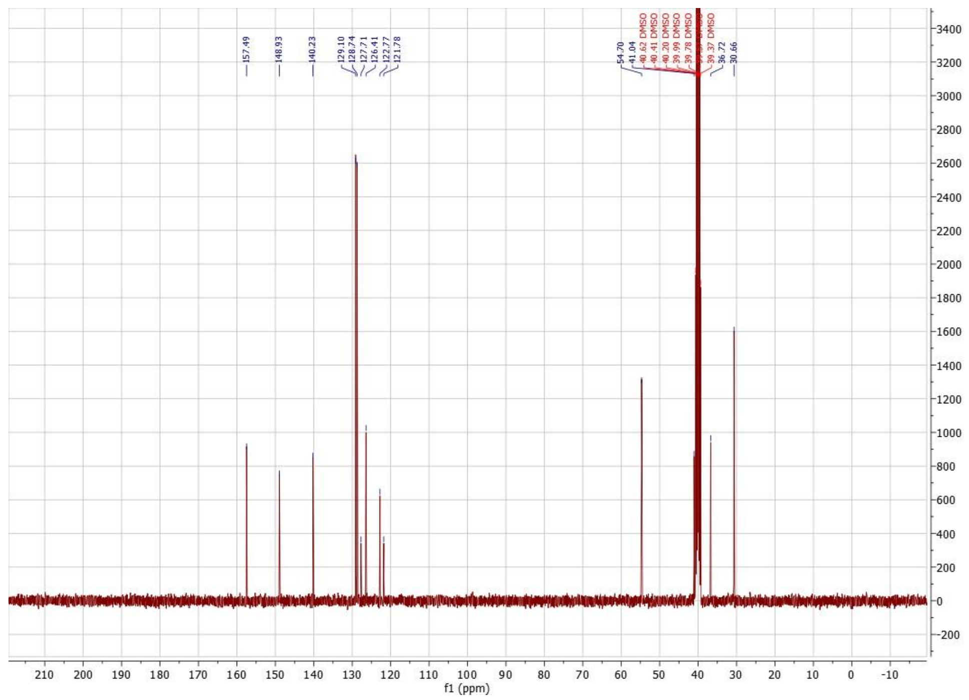

Figure S73 $^{13}C\{^{1}H\}$ NMR spectrum of **9b** in DMSO-d_6.

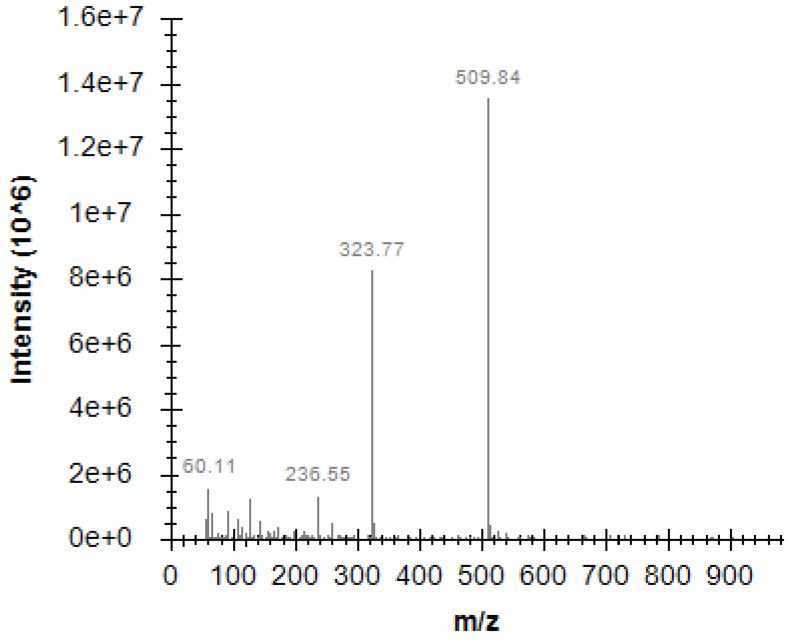

Figure S74 ESI-MS spectrum of **9b** in methanol.

Compound 9c

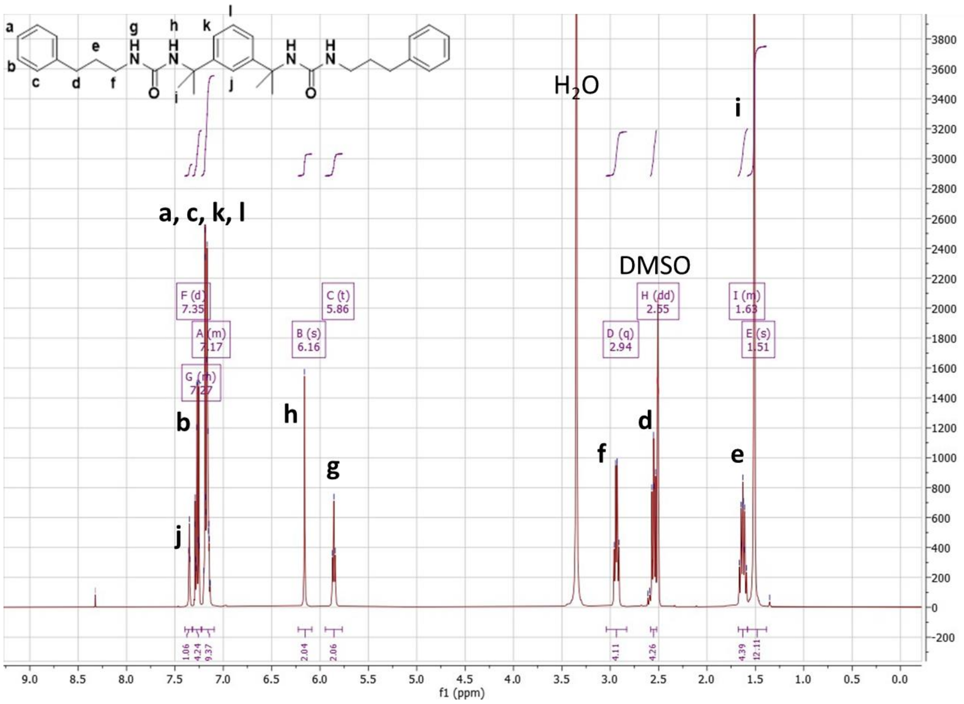

Figure S75 ^{1}H NMR spectrum of **9c** in DMSO-*d*$_6$.

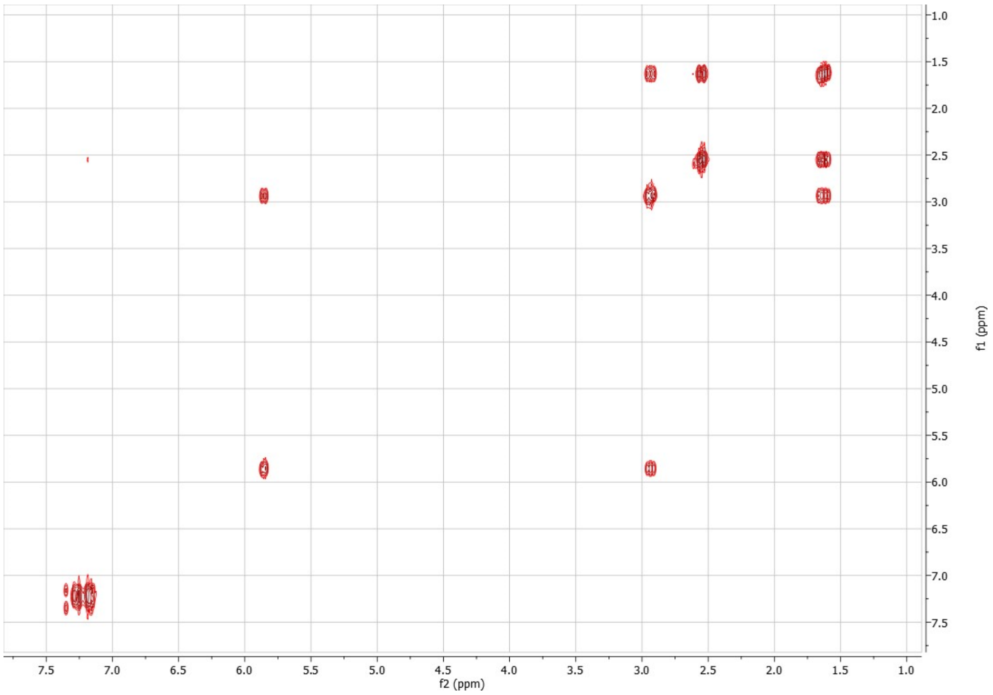

Fig. S76 COSY NMR spectrum of **9c** in DMSO-*d*$_6$.

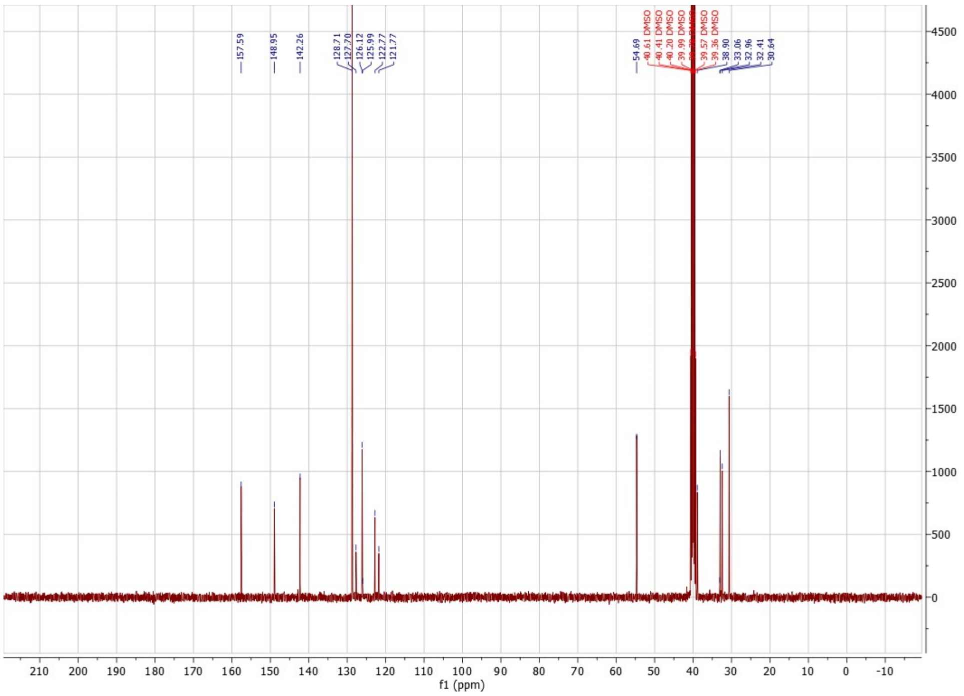

Figure S77 $^{13}C\{^{1}H\}$ NMR spectrum of **9c** in DMSO-d_6.

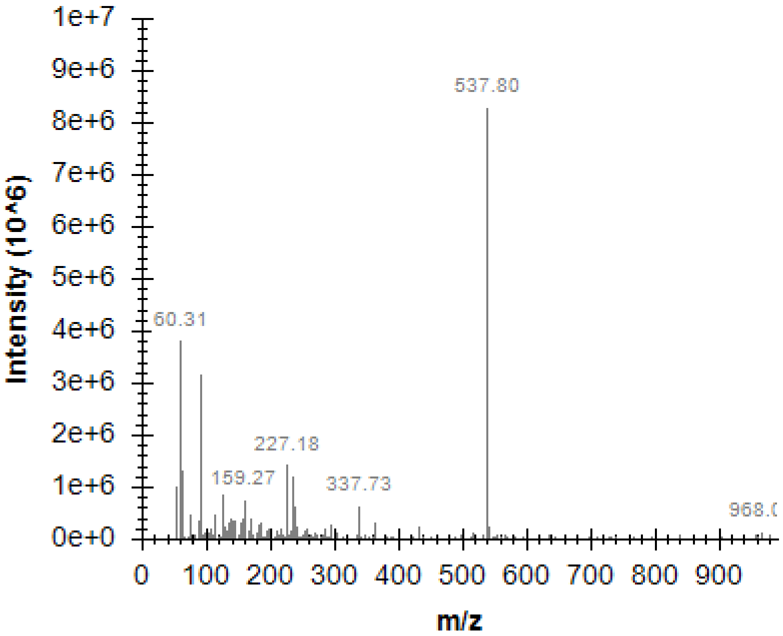

Figure S78 ESI-MS spectrum of **9c** in methanol.

Compound 10a

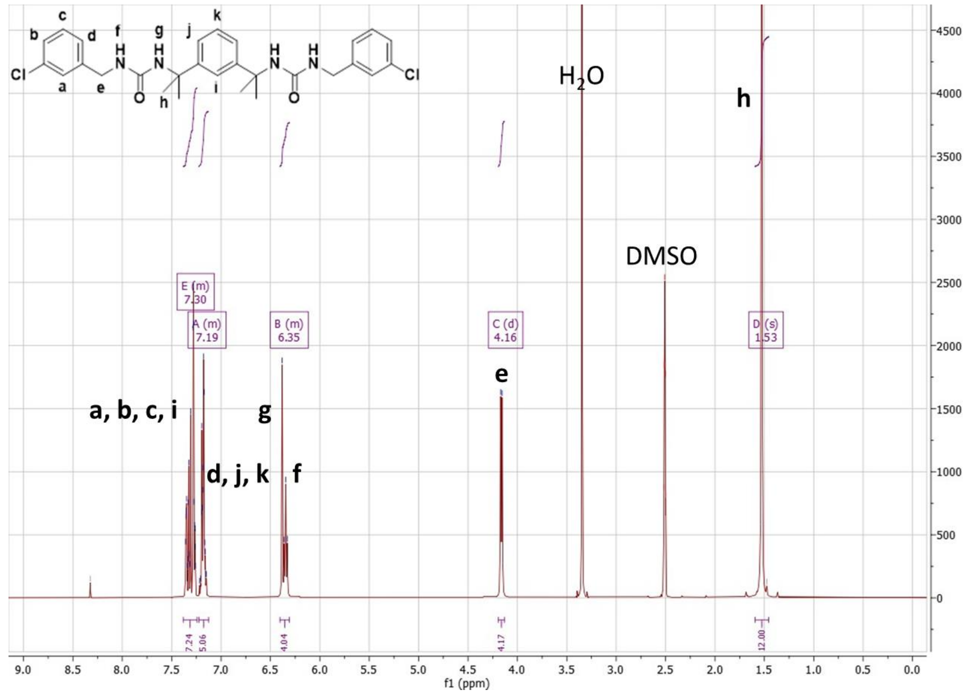

Figure S79 ^{1}H NMR spectrum of **10a** in DMSO-d_6.

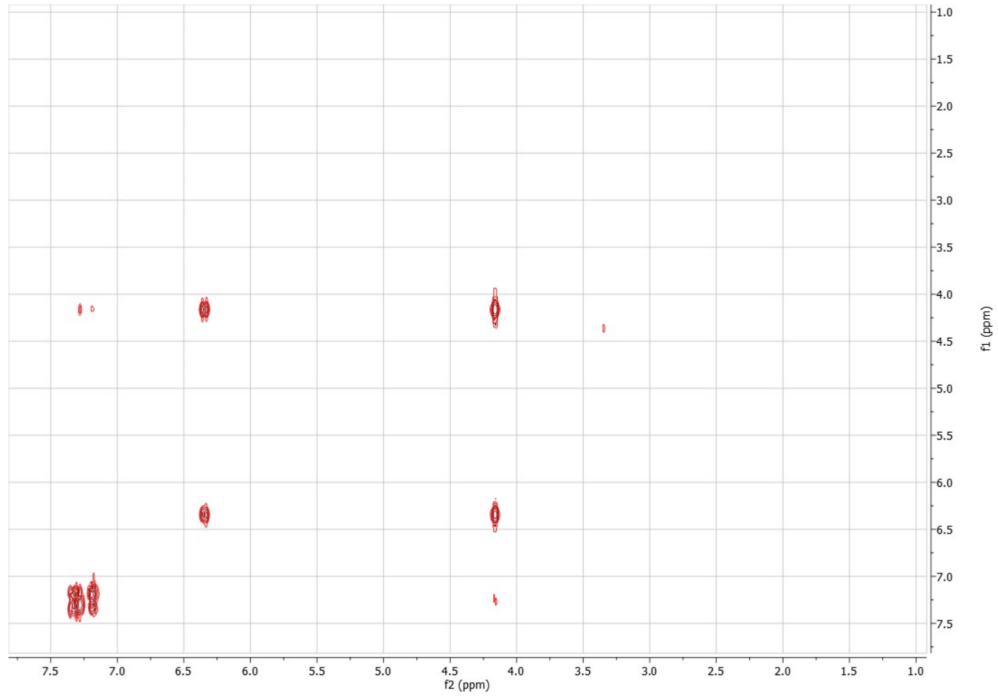

Figure S80 COSY NMR spectrum of **10a** in DMSO-d_6.

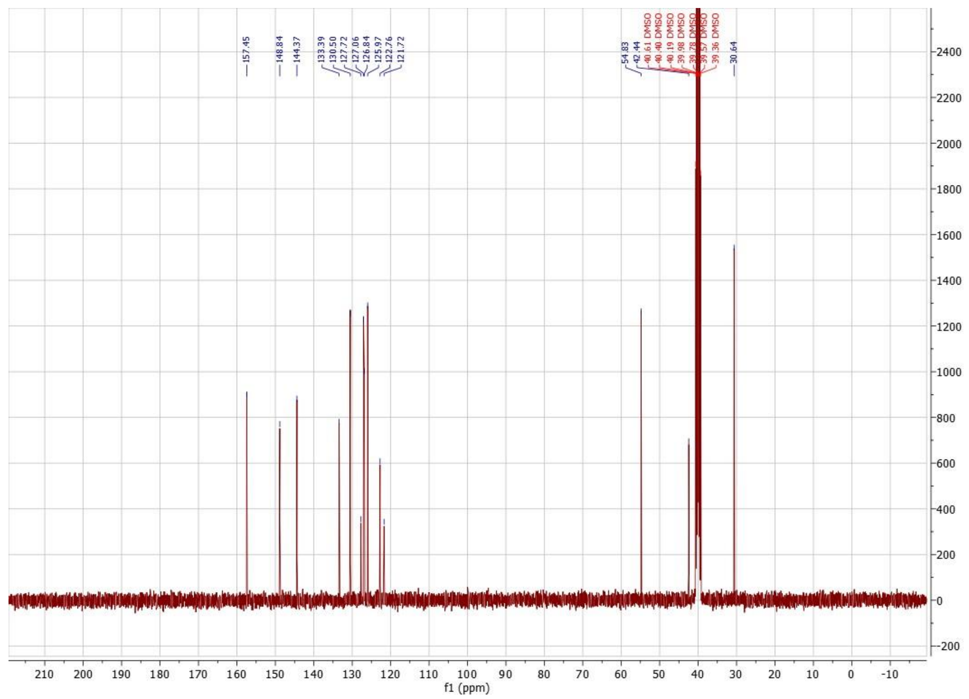

Figure S81 $^{13}C\{^{1}H\}$ NMR spectrum of **10a** in DMSO-d_6.

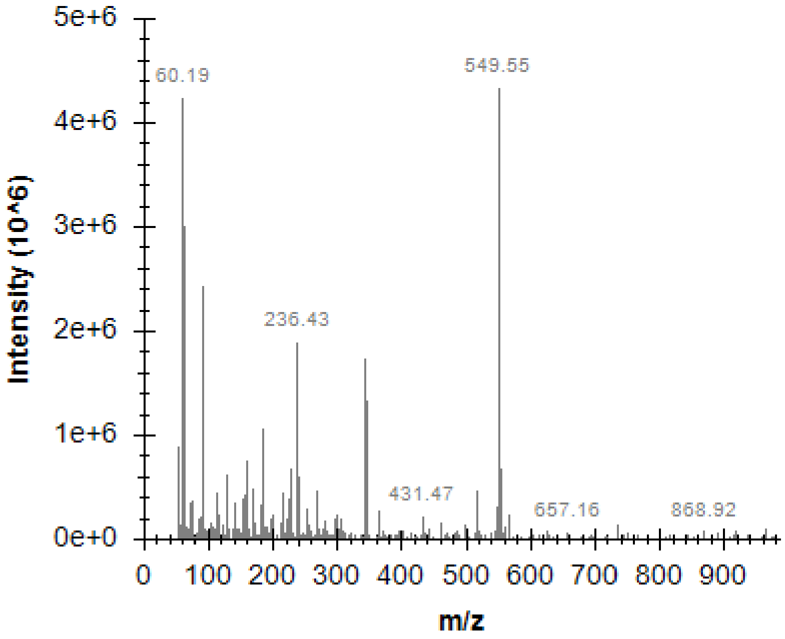

Figure S82 ESI-MS spectrum of **10a** in methanol.

Compound 10b

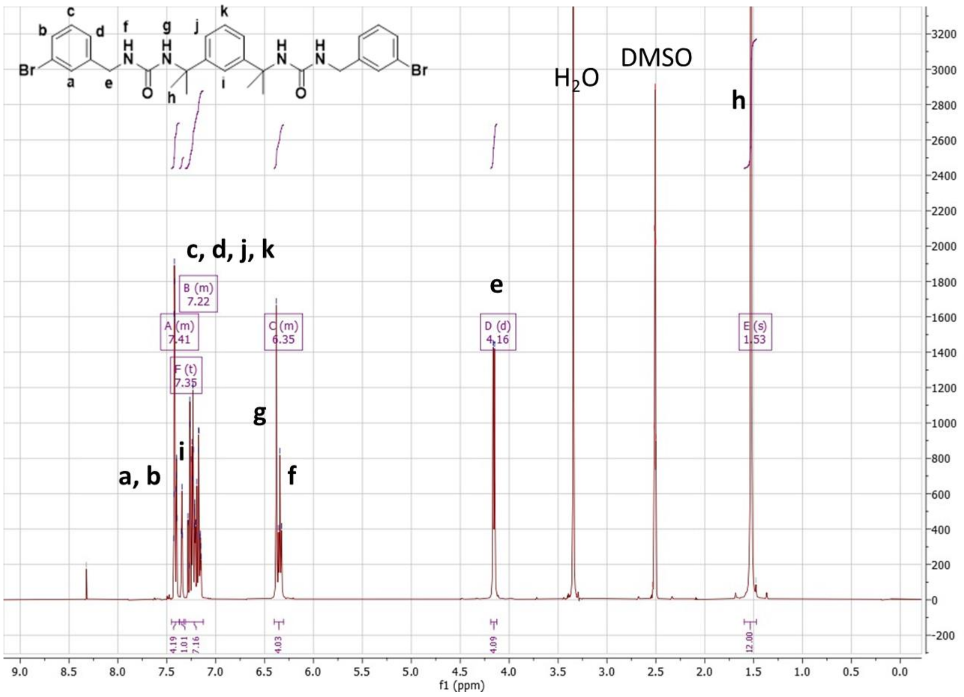

Figure S83 ^{1}H NMR spectrum of **10b** in DMSO-d_6.

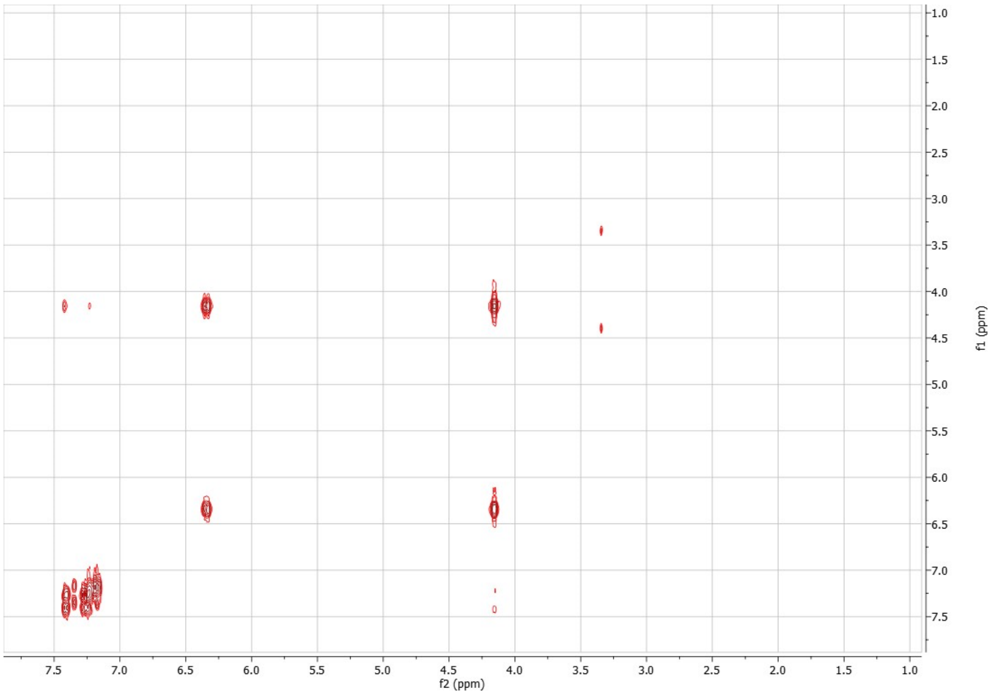

Figure S84 COSY NMR spectrum of **10b** in DMSO-d_6.

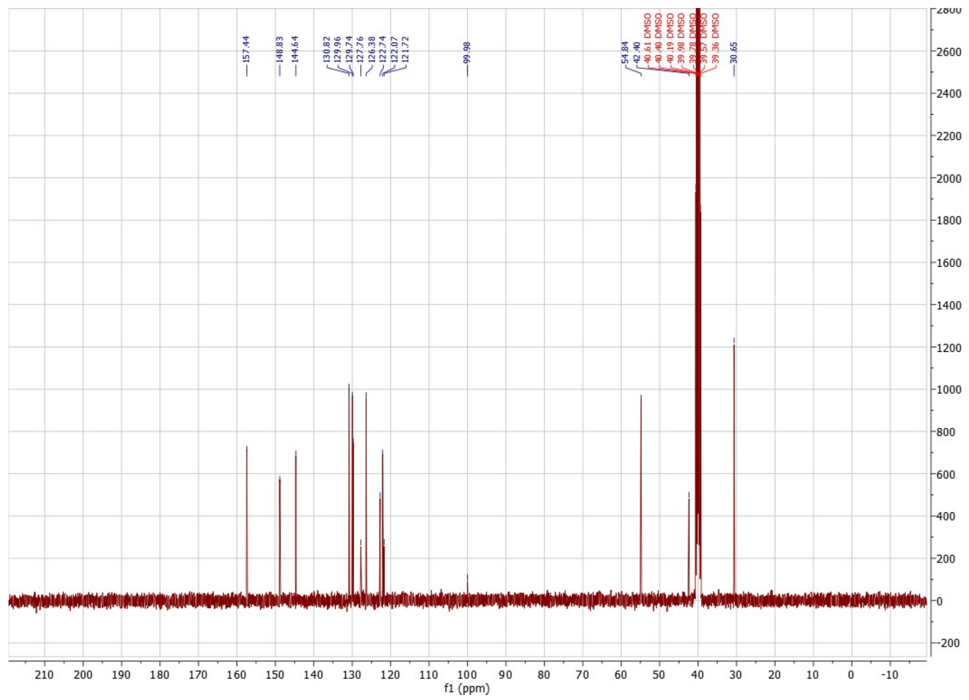

Figure S85 $^{13}C\{^1H\}$ NMR spectrum of **10b** in DMSO-d_6.

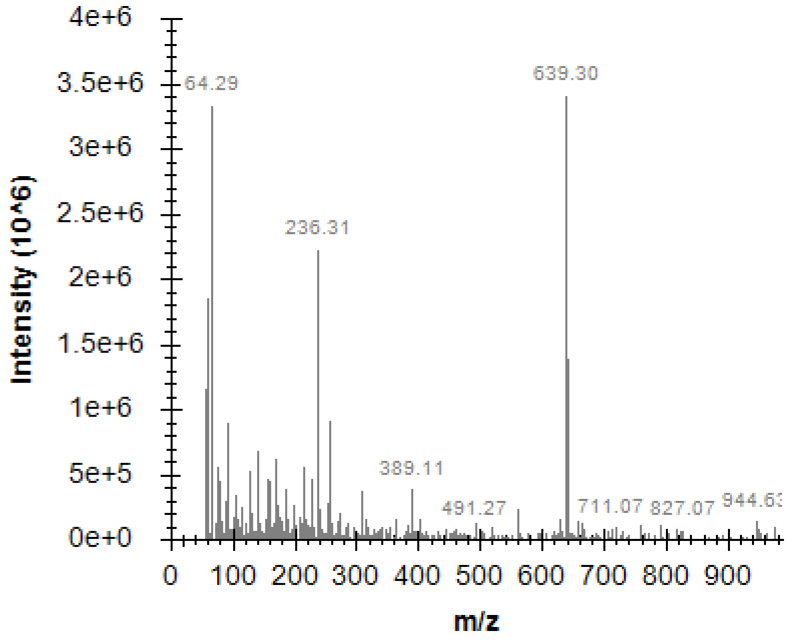

Figure S86 ESI-MS spectrum of **10b** in methanol.

Compound 10c

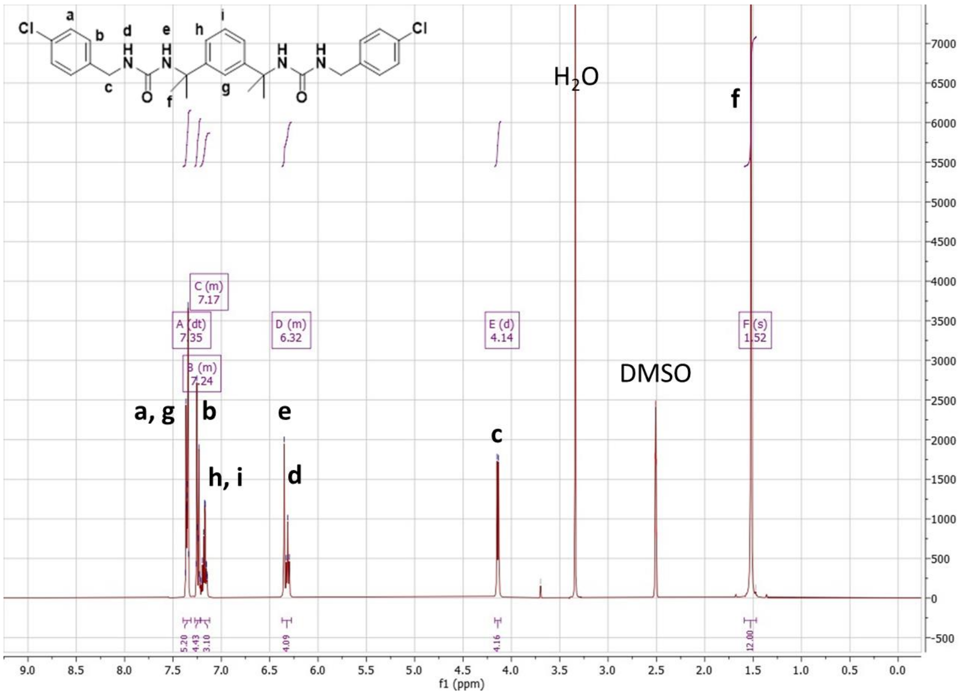

Figure S87 ^{1}H NMR spectrum of **10c** in DMSO-d_6.

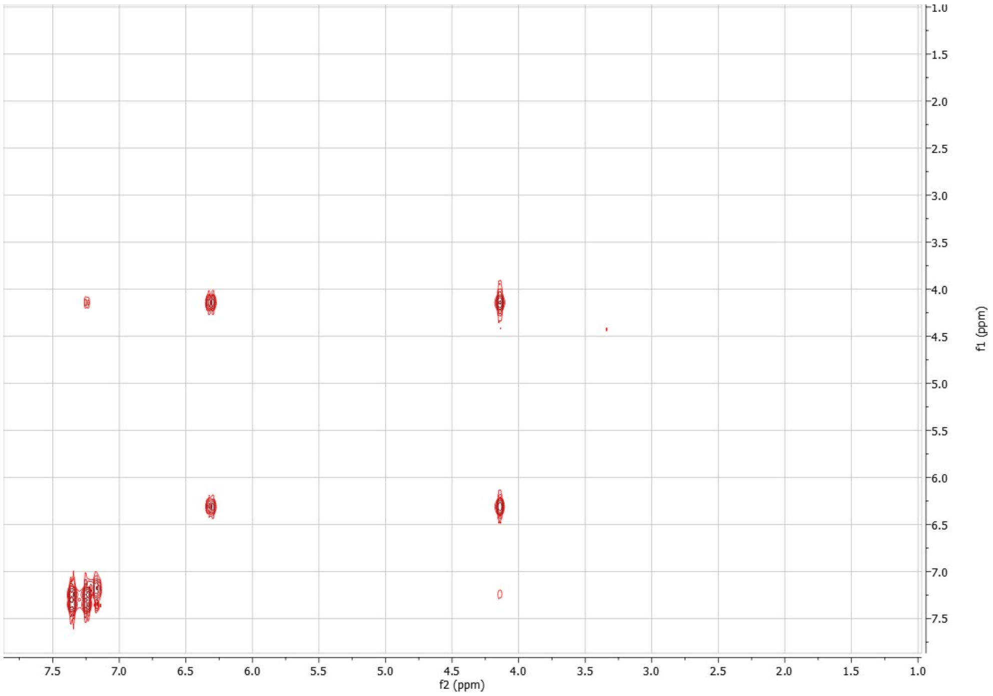

Figure S88 COSY NMR spectrum of **10c** in DMSO-d_6.

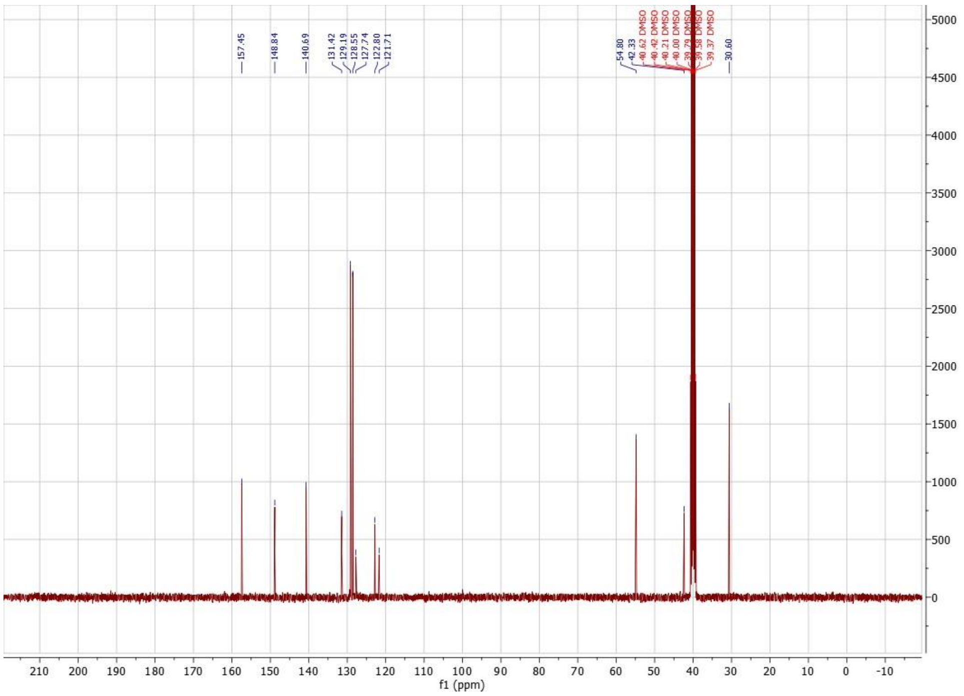

Figure S89 $^{13}C\{^1H\}$ NMR spectrum of **10c** in DMSO-d_6.

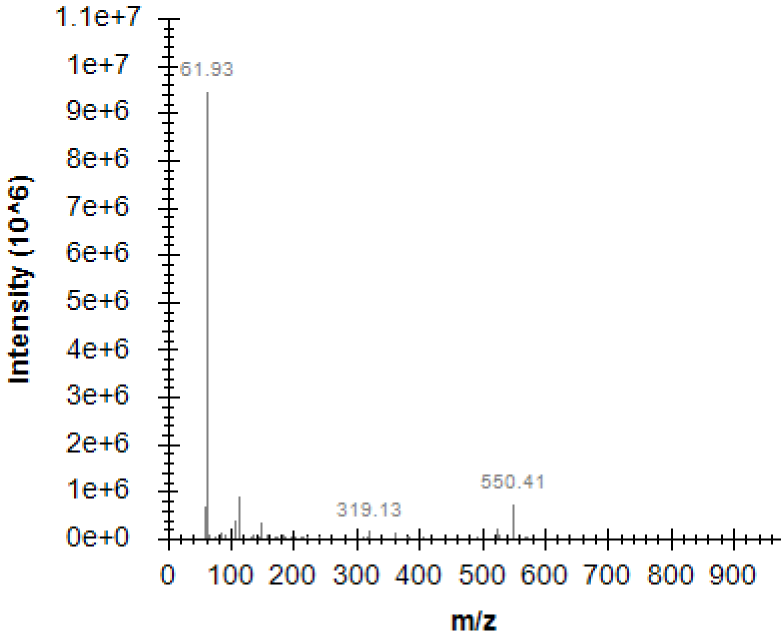

Figure S90 ESI-MS spectrum of **10c** in methanol.

2.2 CSD survey

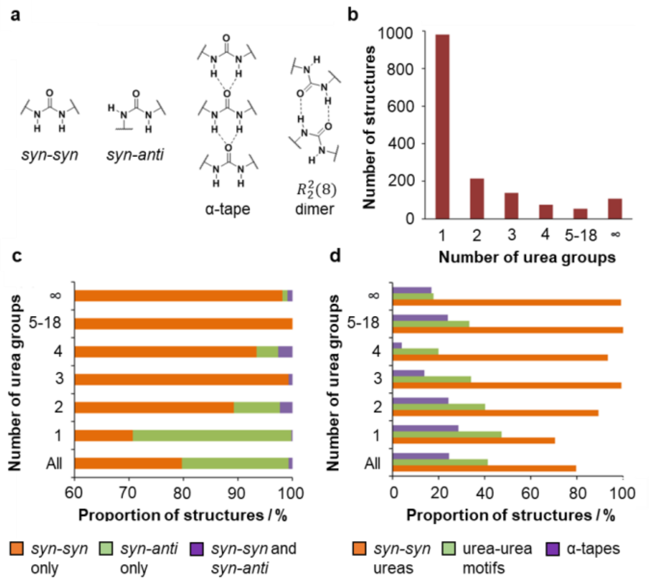

Figure S91 (a) Common urea conformers and supramolecular motifs; (b) frequencies of urea-based structures in the CSD as a function of the number of urea groups; (c) frequencies of *syn-syn* and *syn-anti* conformers; (d) frequencies of urea-urea interactions and tape motifs.

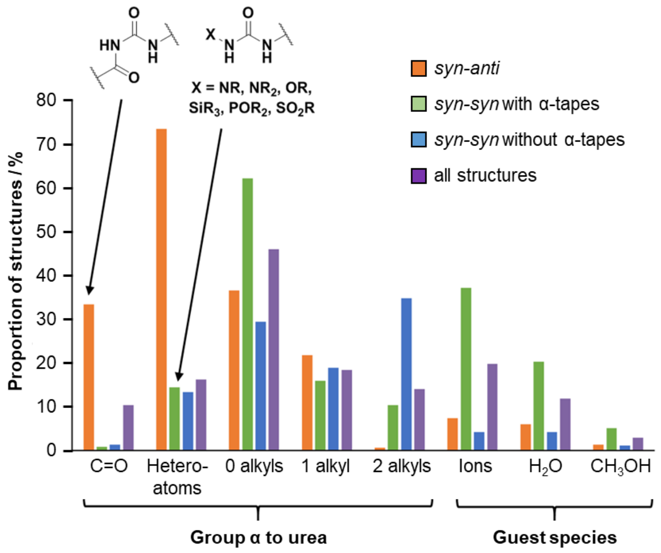

Figure S92 Frequency of key structural features and guest species in mono(urea) systems with different urea conformers and hydrogen bonding motifs.

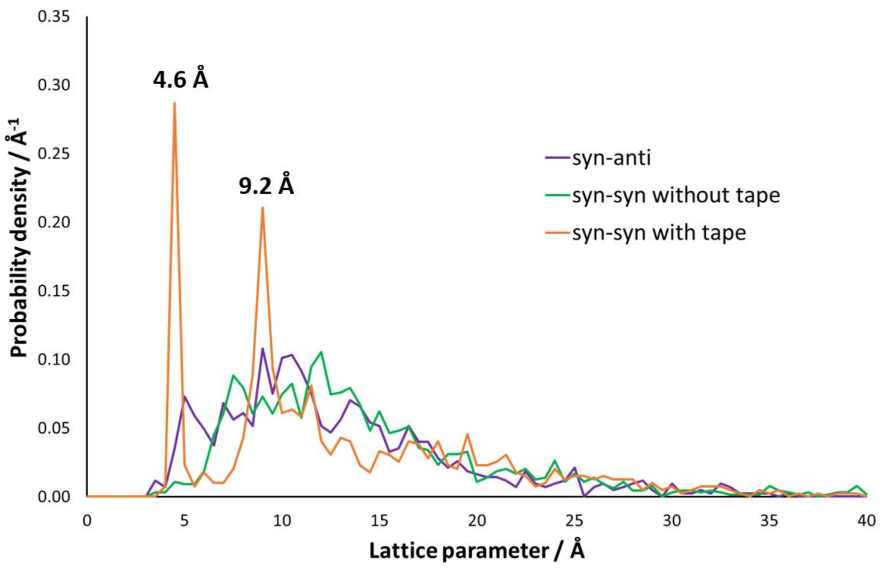

Figure S93 Probability of observing particular lattice parameters for structures with different urea conformers and supramolecular motifs. The probability density is calculated such that the area under the curve over an interval of 0.5 Å (units $Å^{-1}$ x Å) yields the fraction of lattice parameters within that range.

REFCODE	Urea-urea motifs?	REFCODE	Urea-urea motifs?	REFCODE	Urea-urea motifs?
AGOLIS01	Y	IJUXOB	Y	RASGOI	Y
AGOLOY	Y	JEHFAE		RASGUO	
AGOLUE	Y	JEMQOI	Y	RAWBOI	Y
AGOMAL	Y	KASLOH	Y	REMRAD	
AGOMIT	Y	KEVQAF		RENKOM	
ASUQEK	Y	KISNOQ	Y	RETGAA	
AZOMUY	Y	KOCXOQ		REZQUK	
BABHAQ	Y	KOCXUW		REZRAR	
BASXOJ		KOCYAD		REZRIZ	
BAXQAT		LAJDIL		REZRIZ01	
BEHTIT	Y	LARFOB		REZROF	
BEHTOZ	Y	LETLIH	Y	REZRUL	
BIDRUD		LETLON	Y	RIDLUN	
BORQOP	Y	LETLUT		RIYXUU	
BUKSIK	Y	LETMAA		ROCPIJ	Y
CAZBUB	Y	LETMEE		RUSLAU	
CAZCEM	Y	LETMII		RUSPAX	
CIQMEU		LIHYIM	Y	SAGRID	Y
COFKUE	Y	LIHZAF		SAWLAE	
COFLEP	Y	LIHZEJ		SICHOC	
COFLOZ	Y	LIHZIN		SICHUI	
CUCBAD		LIHZOT		SICJAQ	Y
CUTHOQ	Y	LIHZUZ		SIHROS	
DASNUH		LIPBIW	Y	SUBCOJ	
DONQOO		LIPBIW01	Y	SUSPON	Y

DUVZIF	Y	LIPBOC	Y	TOLBUS	
EFIKIO01	Y	LISDIB		TOLCAZ	
EFIKOU	Y	LOBHUH	Y	TOLCED	
EFIKOU02	Y	MEXBIA		TOLCIH	
EFIKUA	Y	MIXFOP		TOLCON	
EJISEW		MIXFUV	Y	TOLCUT	
ELIGAH	Y	MOQNEL	Y	TOLDAA	
EMIRUO		MOWWAY	Y	TORWAZ01	
EQOHIB		MOZJAO	Y	TUJZIJ	
EXESUW	Y	MOZJES	Y	TUJZOP	
EZUKIT01		MUMRER	Y	UJISEM	Y
EZUKOZ01		NADHIL	Y	UJISIQ	
EZUKUF01		NEJVIJ		UJISOW	
FABFAR	Y	NEJVOP	Y	VEGQUV	
FABFEV	Y	NEKZIN		VEGRAC	Y
FABFIZ	Y	NIHROM		VEGREG	Y
FACWIR		NIZHIP	Y	VEGRIK	Y
FACWOX		NOWZEG	Y	VEGROQ	Y
FANWEX		NOWZIK	Y	VEGRUW	Y
FANWIB	Y	NOWZOQ	Y	VIDSOS	
FANWOH		NOWZUW		VIDSUY	
FANWUN		NOXBAF		VIHDAS	
FANXAU		NOXBEJ		VIHDIA	Y
FANXEY	Y	NOXBIN		VIHDOG	
FANXIC	Y	NOXBUZ		VINBEA	
FANXOI		NUPQIA		VINBIE	
FEGVEU		ODAKUB	Y	VINZAV	
FELFAE		ONICUK	Y	VINZAV01	
FELFEI		OQINUY		VINZID	
FELFIM		OQINUY01		VOCYES	
FELFOS		OQINUY02		VOCYIW	
FELGAF		OQIPIO	Y	VOCYUI	
FIJNET		PAVMEF		VOMDAD	
FIWBOD	Y	PAVMIJ		VOMDEH	
FIWBUJ	Y	PEFHOY	Y	VUCTUJ	
FONXUC		PEJKIZ	Y	WESVOG	Y
FONYAJ		PEPROS	Y	WIMSES	
FONYEN		PIBKOB	Y	WIMSIW	
FUWHAI	Y	PIBTUQ		WIMSUI	
GAMQER	Y	POGMAB		WIMTAP	Y
GIRYIQ		POHHOK	Y	WOHFEG	Y
GUDKAR		POJNOT		WOHFIK	Y
GUDKEV		PORGOU06	Y	XAFBAK	
GUTBAZ		POXPAU	Y	XERDED	Y
HAFQIQ		POXPEY		XOGBOM	
HAGVER	Y	PUKCOO	Y	XOLJAK	

HAGVOB		QAVQAG		XOLJEO	Y
HAWQEC		QAVQEK		XUFWIG	
HEHRAP	Y	QENGAS	Y	YEKQAI	Y
HEHRET	Y	QENGEW	Y	YOJZUU	
HEKWOL		QIBJAN		YOXQOS	
HICWOH		QOCLAW		YOXQUY	
HIVVOY	Y	QOCLEA		YOXRAF	
HIWPEI	Y	QOXRAY		YOXREJ	
HOMSUZ		QOXREC		YUZHEI	
HOMTIO		QUSBUD	Y	ZAHFUM	Y
HOSNOU		QUSCEO		ZITREB	
IDEMOV	Y	QUSDEP	Y		
IDEMUB	Y	QUSWIM	Y		

Table S1 Bis(urea) structures in the Cambridge Structural Database (CSD), version 5.37[11]. There are 250 well-defined, inequivalent structures containing exactly two acyclic urea moieties. Of these, 102 (41%) display urea-urea interactions. Most urea-urea hydrogen bonds (69 structures, 68%) are found in continuous tape motifs.

REFCODE	Repeat	Pairs of syn-parallel tapes?	Polar space group?	All tapes syn-parallel?
AGOLUE	A			
AGOMAL	A			
AGOMIT	A			
ASUQEK	A			
AZOMUY	A	Y		
BABHAQ	A			
CUTHOQ	A			
EFIKIO01	A		Y	
EFIKOU	A		Y	
EFIKOU02	A		Y	
EFIKUA	A	Y	Y	Y
FABFAR	A			
FABFEV	A	Y		
FABFIZ	A	Y		
FANXIC	A			
FIWBOD	A			
FIWBUJ	A	Y		
FUWHAI	A			
HIWPEI	A	Y	Y	Y
IDEMOV	A			
IDEMUB	A			
LETLON	A	Y		
LIPBIW01	A	Y		
NEJVOP	A			
NOWZEG	A			
NOWZIK	A			
NOWZOQ	A			

ODAKUB	A			
PEJKIZ	A			
RAWBOI	A	Y	Y	Y
ROCPIJ	A		Y	
SICJAQ	A			
VEGRAC	A	Y	Y	Y
VEGREG	A	Y	Y	Y
VEGRIK	A			
WIMTAP	A	Y		
AGOLIS01	AB			
HIVVOY	AB			
MOWWAY	AB	Y	Y	
ONICUK	AB	Y		
PEPROS	AB		Y	
PIBKOB	AB			
UJISEM	AB			
VEGROQ	AB	Y		
VEGRUW	AB	Y		
XOLJEO	AB			
AGOLOY	AABB			
FANWIB	AABB	Y	Y	Y
LETLIH	AABB		Y	
PUKCOO	AABB			
JEMQOI	ABC		Y	
QENGAS	ABC		Y	
LIPBIW	AABC	Y		
SAGRID	ABCD	Y	Y	
BEHTOZ	1 tape	N/A		
IJUXOB	1 tape	N/A	Y	Y
LIPBOC	1 tape	N/A		
NIZHIP	1 tape	N/A		
PORGOU06	1 tape	N/A		
QUSBUD	1 tape	N/A		
QUSDEP	1 tape	N/A		
WOHFIK	1 tape	N/A		
YEKQAI	1 tape	N/A	Y	
ZAHFUM	1 tape	N/A	Y	Y
**Total**	**64**	**19**	**19**	**8**

Table S2 Bis(urea) structures in the CSD (version 5.37) with urea-urea tape motifs. Note that only structures occupying one of the 68 polar space groups may exhibit urea tape networks in which all tapes are syn-parallel (i.e. an entry of “Y” in column 5 must be accompanied by entries of “Y” in columns 3 and 4). In networks categorised as “1 tape”, each molecule forms only one tape.

Repeat	Total
A	36
AB	10
AABB	4
ABC	2
AABC	1
ABCD	1
1 tape	10

Table S3 Frequencies of different bis(urea) network topologies in the CSD (version 5.37).

ADEZEP	DOWTAL	KEWSOW	POPMOX	UYUTIS
ADIFAV	DUCSEA	KIDXEC07	POPMUD	VABQUM
AFIPIQ	DUNTUC	KIVLIM	POQYIE	VAPGOI
AFIPIQ01	EGUDIV	KIVLOS	PUCGLR10	VATLIN
AGOKIR	EKAXOD16	KOSHEF	PUQYOQ	VATLOT
AKELIM	EKECAY02	KPRCGM20	PUQYOQ01	VATLUZ
ALEFUT	ELAPIR	KUDZIS	PUQYOQ02	VATMAG
ALEGAA	EMIPEW01	KUGCUK	PUQYOQ03	VECSAZ
APUXUF	EPEVEB	LAKXOM	PURTRE	VILLUZ
AQETIZ	EVEQEC	LELROJ	QACLAH	VIMGUV
ASITEC01	EVEQEC01	LIDCEH	QAJSIE	VINYEW
AWUGIJ	EVIQEF	LIDLUH	QAJSOK	VOCBOG
AYUHEI	EYUJEO	LINSEG	QALXAE	VODWER
AZUMIS	FABPUV	LIWBOJ	QALXEI	VORBAF
BAQJUY	FIVRAD	LOFDUH	QANYIO	VUZLIM
BEBLOJ	FUHQEF	LOFFAP	QAZXEU	WEFQAB
BEFSIO	FUZQEX	LOFFIX	QAZXIY	WERTET
BORHAS	GAQQAS	LOFWAG	QEHDUC	WEXBEG
BORHIA	GEJHEJ	LOHSOR	QERXUG	WEXMOD
BORHOG	GIDTIW	LUKHOP	QIBHEO02	WICBAL
BOTRIL10	GIHMER	MAPJOD	QIRJEG	WOGXUM
BPCTHA	GIHMUH	MEXBOG	QIVNUG	WOKNOB
BUDSOJ	GIJVEC	MIXGAC	QUCWOB	WOMGUC
BUFXOP	GIYJEE	MOCNIC	QUCWUH	WOMHAJ
BUXFEG	GOFBEK	MOQNAH	QUCXAO	WOMHEN
BUXFEG01	GOFBIO	NAFVIA	QUCXES	WOYHIB
CABCOX	GORBOE	NAFWAT	QUCXIW	WOYQOQ
CABCUD01	GORBOE01	NAXHUR	QUCXOC	WUNNUP
CBOHAZ04	HABTEJ	NAXJAZ	QUCXUI	WUPYAI
CBOHAZ05	HAMCAB	NAXJON	RABYOJ	XAVNAL
CDSCBA	HANFUY	NAXJUT	RACCOO	XAVWIC
CDSCBB02	HAYRAB	NEMRON	REHFIV	XAYHUA
CDSCBC	HOBXOM	NEPKIE	REHFOB	XAYJAI
CDSCBD	HOBYAZ	NEVXIV	SAKCIQ	XAYSUL
CDSCBE	HODHAK	NICGEL	SERXAO	XELQUB
CEJTEQ	HOFDAI	NICGOV	SISSOE	XENHEE

CEJTIU	HOFKAP	NIDYUW	SOBCUI	XENSOY
CEVSAY	HOGYUZ	NIWFOQ	SOBPEE	XEQDEC
CIDGIH	HYXBUR10	NUCDIY	SOBPEE01	XERDIH
CIGSES	ICAKAY01	NUVCAI	SOBPEE02	XIGLAB
CIJFOS	ICILUC	OBUNAC	SOWQIE	XIVDEN
CIWMEC	IFAFEB	OCAKUZ	SUVGUL	XOWNIH
CIWWEM	IFEPAL	ODOMIE	TAWPUD	XUXNOT
CIZLUS	IHOWEI	OGECIN	TEDBUZ	XUYTUG
CNBPCT	INUGOO	OGIQIF	TEHBIR	YAGWAG
COGNIX	ISAGUF	PACNEO	TEJNEC	YAXZAA
CORZEQ	IWUXAA	PASHUN	TEVMOX	YAYYON
COYNOU	IWUXEE	PAWPAG	TEWJOV	YEJQUZ
CPDPSC11	IZOLIT	PAWXUI	TIRYIC	YIFVIT
CPFBUR01	JAHSUG	PCMTRE10	TOHBUN01	YIHRUC
CUGHUH	JAXGEV	PCTRIB10	TOLMOX	YINCAB
CUQTIR	KABMOR	PELKAT	TOLPUF	YINFOS
CYCOXB	KAJSUK01	PELKEX	UJIHAX	YIVGUG
CYNONB	KARTED	PELKIB	UJOLAH	YOCVOC
DATKAL	KATBIS	PELKOH	URAWUG	YUZHUW
DELRES	KATFAN	PIFZAG	USOCOV	ZEWDUC
DEMDOP	KEMZUZ	PIFZAG01	UXAYAU	ZINCEG

Table S4 Structures in the CSD (version 5.37) containing *syn-anti* mono(urea)s.

ABIBEU	EXAHEQ	KAZVEN	QEBBOQ	VIPBED01
ACURUN	EZAXOS	KAZWAK	QEMFEU	VIRTUN
ACUSAU	EZAXUY	KEDREQ	QEYBOM	VIXMOF
AGOKOX	FAWZEK	KETSUY	RAVVAN	VIXNAS
AGOLAK	FAWZOU	KOMBEV	RAVVER	VOBNIJ
AGOMEP	FAXBAJ	KOXPIW	REHQUR	VOFDAX
AJUJOF	FAXBIR	KUWHIT	REMWOV	VOHCEC
AJUJUL	FAXBOX	LAXJOK01	REPRUZ	VOKXAW
AKENOU	FEBWUG	LAXJOK02	RESRIR	VOYNUS
AKENUA	FEMQEU	LAXJOK03	RIPBOH	VUFWID
ALOFOW	FEMQIY	LAXJUQ	RISVEW	VUKJIV
APURIN	FEZWUE	LAXKAX	RISWUN	VUSPUV
AQOSAB	FITYAI01	LEPMAU	RIXBEH	VUSQAC
AXAWIG	FODXUT	LIDCAD	ROCNIH	VUZFUS
AZUQOB	FUDJAQ	LIKNEY	ROCPAB	VUZZAS
BAGPII	FUDJIY	LIPBUI	ROFGIE	VUZZUL
BEJTEP	FUDJOE	LIPCAP	ROHKOO	WAMGAU
BESCAD	FUWHIP	LIYLAI	RUJDOP	WARXOC
BIMWUQ	GAKGAA	LIYLEM	SAGXOP02	WARYAP
BOFXOK	GAPJAK	LIYLIQ	SAGXOP03	WEGLUP
BOFXUQ	GASMUJ	LONXIX	SAYQEP01	WEGQEG
BOJTIF	GASNAQ	MAHWAV	SEPQEJ	WEGQIK
BOJTUR	GASNEU03	MAMPUR	SEPTIQ	WEGQUW

BONLAS	GASNIY01	MBECUR	SEPTUC	WESKUB
BOZYOF	GAWZUB	MEHGAH	SIFDER	WESVEW
BUXVAS	GAXBAK	MEKSEA	SILVAK	WESWAT
CAGPIL	GEBFEA	MEPWUZ01	SILVOY	WETDIJ
CAZLEW	GIDTES	MEPXAG	SIQTOD	WICBEP
CBHYZS	GIHKUF	MEPXEK	SLFURE	WICBIT
CEGNOT	GIMROJ10	MEWMOQ01	SOBLUQ	WICNAY
CEKBEB	GIMRUP10	MICZII	SOBNOM	WIGJEC
CIBQIN	GIMSAW10	MIPLUT	SOHNAF	WIVGUF
CILFEJ	GIMSEA10	MIPMAA	SOKVOE	WIXYUZ
CIVRUV01	GOJBUC	MUWQIF	SOQXUR	WIXZAG
CIWNON	GOKXAF	MUWQOL	SUNHUF	WIXZEK
CIWPOP	GOLWUA	MUWQUR	SUZNIL	WIXZIO
CIWVIP	GURHUX	MUZSEG	SUZWOA	WOCFAW
COMZOT	HAFKOO	MXYLUR	TAMSUX	WOCFEA
CONKUM	HAMCEF	NATGIA04	TEPNEH	WOCFIE
CONLEX	HAMCIJ	NEBBIG	TERYIY	WOCFOK
CONMOJ	HASCIN	NEBBOM	TIFLOK	WOCFUQ
CONMUP	HEZTEL	NEBGEH	TIHTIO	WOCLIL
CONNEA	HIFZUR	NEBYID	TIMXIY	WOGZIC
CUCFAH	HIMPEZ	NEBYOJ	TIVMAN	WOGZOI
CUCFEL	HINWAE	NEBZEA	TIVMER	WOGZUO
CUKPUU	HITFOG	NEBZIE	TIVMIV	WOHBAX
DACPEE	HITFUM	NEGCOR	TIVMOB	WOHBEB
DARSIA	HITGAT	NEHHIT	TIVMUH	WOLPUJ
DARSOG	HITGEX	NIPXAN	TIVNAO	WOLQIY
DASYUS	HOMSOT	NIQFID	TIVNES	WOMHIR
DAZJUL	IDUSIK	NODYEM	TIVNIW	WOMHOX
DAZKAS	IFANOU	NUSVIH	TIVNOC	WOMHUD
DAZKEW	IFANUA	NUSVUT	TIVNUI	WOMJAL
DAZKIA	IFOCEM01	OVEBAT	TIVPAQ	WOMJEP
DEZGIA	IKEGIP	OVEBEX	TIVPEU	WOMJIT
DEZGOG	ILICEL	OVEBIB	TIVPIY	WONZAC
DIGBUR	ILICIP	OVEBOH	TIVPOE	WONZIK
DIGCAY	ILIDEM	OVEBUN	TIXKES	WUDMAK
DIGCEC	INUGAZ	OVECAU	TORSEM02	XANPOT
DIGCIG	IQOLEG	OXULOJ	TORSEM03	XATNIQ
DIGCOM	ISODEZ	OXULUP	TOSBUR	XAZGEM
DIGCUS	IXUDIO	OXUMAW	TUJNUH	XEFFAR01
DIGPIU	JANKOY	OXUMEA	TUJPAP	XEWXON
DIGPUG	JEDKIN02	PAMPOK	TUPMAS	XEXFEL
DIGQAN	JEDKOT	PAMPUQ	UCOHUQ	XEYYOQ
DISBOW	JEDKUZ	PAQLID	UDEJUK	XINHIN
DISBUC	JEDLAG01	PEFGOX	UDEKAR	XOLHIQ
DISVAD	JEDLEK	PEVREP	UDEKEV	XOMBEG
DIVYOY01	JEDSER	PEZDEE	UDEKIZ	XOPKIW

DIWBUI01	JEDSIV	PEZJUA	UJIGUQ	XUCDOO
DIWDAQ02	JEDSOB	PIHFOB	UJIHEB	YAFJOG
DIWDEU02	JEDSUH	PIHLAU	UJIHIF	YEKNUZ
DOJXEG	JEDTAO	PIHLIC	UJIHUR	YEKPAH
DUNXAL	JEDTES	PIHLOI	UJIJAZ	YEKPEL
DUQGOM	JEHFIM	PIHLUO	UJUVEA	YEKPIP
DUTNEM	JODXAC	PIHMAV	UKIFIE	YEKPOV
DUXBOO	JODXEG	PILFUM	ULIXOD	YEKPUB
ECATOT	JOJZOZ	PIXNER	UXIPOH	YEKQEM
ECATUZ	KABCUN	POJNIN	UYOZUE	YIGQAH
ECAVAH	KAQRUR	POKDIC	VACDIO	YITFEN
ECAVEL	KAQSAY	POQVAT	VIJDID	YIXDIT
EHEHEF	KARTAZ	POQYUQ	VIMQEN	ZAQROA
EKEJAF	KARTIH	POYZOT	VIMZAT	
EWEMEY	KAXJAW	POYZUZ	VIMZEX	
EWEMIC	KAXJEA	PPESIR	VIMZIB	
EWOZUL	KAXJIE	PUHHAC	VINBOK	
EXABIO	KAZVAJ	QAZXUK	VIPBED	

Table S5 Structures in the CSD (version 5.37) containing non-interacting *syn*-*syn* mono(urea)s.

AGOKUD	FABDUJ	MOQCAX	SIGBUF	WANPEH
AMAFEZ01	FATWAY	MUBHIB	SILTOW	WARWIV
AQASIV	FATWEC	NAXJIH	SILTOW11	WARWOB
AQATIW	FEBXAN	NEBYUP	SILTOW12	WARWUH
AQAVIY	FEYSEJ	NERPUX	SILTUC	WARXAO
AQAWUL	FICKAF	NIJHUJ03	SILTUC01	WARXES
ASITEC	FICTIU	NIJHUJ04	SISSOE01	WARXIW
AZUDAB	FIQSEE	NOEURA	SUZKII	WARXUI
BAHPIK	FOLRUU	NUQFIP	TAPRAD	WARYET
BATQUK	FUDJEU	NUSVAZ	TEPNEH	WARYIX
BEDMIG01	FUMQIN01	NUSVED	TIVPUK	WEGQOQ
BEDMIG02	FUWJAJ	NUSVON	TIVQAR	WEJBUI
BEDMIG03	GAKGAA	NUVRUS	TIVQEV	WESVIA
BEDMIG06	GEKFEJ	ODAKOV	TOHBUN02	WESVUM
BEDMIG07	GIJYUT	ODIRIE	TOSMUR	WESWEX
BEDMIG11	GOKWUY	OJIYAI	TRURET20	WESWOH
BEDMIG12	HAHCOJ	OJOWOA	TUDLUZ	WEVZED
BEDMIG16	HAWLIB	OKOSUD	TUDLUZ01	WEWRAR
BEDMIG18	HEVXAH	OTEBOF	TUHCUV	WIGJIG
BEQNAN	HEZSUA	OZIVAV	TUHPIV	WIXMEW
BOJCUY	HEZTAH	PAYQAI	TUNZAE	WONZEG
BOZZUM	HORPUB	PEFGIR	UCOYAM	XAVNEP
BUVHOP	IFANIO	PIHLEY	UGIWOX	XAYSOF
BUYZIE	IFEHEG	POTLIT	UJOBEA	XEBRUS
CAJQEK	ILICAH	POTLOZ	VAJFUI	XEGDES
CAJQIO	IPPSUR	POZWEG	VALPII	XEWXIH

CAJQOU	IWEHIB	QAXSUD	VALPII01	XINRIV
CAJRAH	IZUVII	QAXSUD01	VAQMIK	XINRIV01
CAJRIP	JADHEC	QAZXOE	VAQMIL	XINRIV02
CEGBAT	JEDRUG	QIYMEQ	VARGIF	XISNUI
CIMKUG	JEDSAN	QONSET	VEGSAD	XIXQOL
COCMEN	JEDTIW	QOQXOK	VEJBOB	XNTLUR
CONKOH	JODVED	QQQAFY01	VEPKIL01	XOMLIV
CONLAT	JUPDED	QQQAGA04	VEPTUG	XOYYEP
CONMID	KAXHUO	RABVIV	VICZIR	XUGNAO
CUNCEU	KEVRAG	RALGAO	VIMYEV	XURBIW
CUSWUJ	LAGKIP	REKDAM	VINZOJ	YALVOX
CYHXUR08	LAGKOV	REKDEQ	VINZUP	YEHHUQ
DATTUN	LAJFAF	RIPBOH01	VIRVAV	YIJBEZ
DATWOK01	LECLEL	RIPBUN	VOFCOK	YIJBID
DCPHUR	LEGZUT	ROCNON	VOFDIF	YIKDAZ
DEDDOG	LENXOT	ROCNUT	VOFDOL	YUNRIJ
DEDFAU	LIDJEP	ROHQAI	VOFFAZ	YUYSAM
DEVVUV	LIWRIT	RULPOD	VOPDUA01	ZINZUR
DIDVES	LIWROZ	SAGXOP01	VOPFAI	ZIPBAB
DPCBHZ	LIWRUF	SARZAM	VOPFEM01	ZIPBEF
DPUREA09	LIWSAM	SARZOA04	VUKHUF	ZIPBIJ
EFETEQ	LIWSEQ	SEPQIN	VUKJAN	ZZZPUS04
EFISOD	MAHWEZ	SEPQOT	VUKJER	ZZZPUS05
ETOBIY01	MBZAUR10	SIGBAL	VUSPOP	ZZZPUS10
EYOMAG	MEPXEK01	SIGBEP	VUSQEG	ZZZPUS13
EZAYAF	MILTOS	SIGBIT	WAMXEN	
EZAYEJ	MIXZAV01	SIGBOZ01	WANPAD	

Table S6 Structures in the CSD (version 5.37) containing interacting *syn-syn* mono(urea)s.

BAWJIU	***GERVUW***	OHOGOH	SASKEE	XAGRUS
BAWJOA	GIXZOD	OHOGUN	TEFZUA	XEWTOK
BAWJUG	GIYCAT	OHOHAU	TEGBAJ	XEWTUQ
BULKUP	GIYCEX	OKOKIJ	TEGBEN	XEWVAY
BULLAW	GIYCIB	OKOKOP	TOMJEM	XIBQEG
BULLEA	GIYCOH	OKOKUV01	TOMJIQ	XIBQIK
CAZCAI	GIYCUN	OLAVIH	TOMMIT	XIDBES
CEVWOQ	GIYDAU	OLAVON	TOMMOZ	XIDBIW
CEVXIL	GIYDEY01	OLAVUT	VAMJUQ	XIDBOC
CITYIO	HANWUQ	OLAWAA	VAMKAX	XUFFEK
CITYOU	HIBPIT	ONERAB	VIFYIU	XUFFIO
DAQWEZ	HIBPOZ	ONEREF	VIFYOA	YACJAP
DAQWID	HIJFOW	ONERIJ	WANKUT	YEMJOQ
DAQWOJ	HIJGAJ	OWISUJ	WANLAA	YIXJAS
DASZAZ	HIJGEN	PUSTIH	WANLEE	YIXJEW
DASZED	ISOWAP	QAMHIV01	WANLII	YIXJIA
DASZIH	ISOWET	QIQSIU	WANLOO	YIXJOG

DIXWIR	IWEHAT	QIQSOA	WANLUU	YIXLAU
DIXWOX	IXEMON	QIQSUG	WANMAB	YOZQIO
DOKDAJ	KECHEG	QOFVIS	WANMEF	YOZQOU
DUVLEM	KEVQEJ	QOFVOY	WANMIJ	YOZROV
EBONUF	KEVQUZ	QOLKIL	WANMOP	YUSDIA
EGUFER	KIKCUE	***REPQOU***	WANMUV	YUSDOG
EHIXID	MINTUY	RIBKIY	WEWTEX	YUSDUM
FABPOO	NEHGOY	SAKBIR	WIDXEO	***ZATBON***
FABPUU	OCUPAF	SAKBOX	WITMUJ	***ZATBUT***
FAFYUI	OCUPEJ	SAKCUE	WITNAQ	
FUXYIH	OCUPIN	SASKAA	WITNEU	

Table S7 Structures in the CSD (version 5.37) containing tris(urea)s. Structures consisting of linear molecules are shown in bold italics; molecules in the remaining structures are multipodal.

AXEYAD	***DEXROP***	KEYKEG	***QIFPUS***	VADDAH
AXEYEH	***ESICOZ***	KICZIG	***REPQAG***	VATXIX
BEPSOG	***ESICUF***	LISCAS	***REPQUA***	VATXUJ
BEPSUM	EZULAM03	LISCIA	SAWLEI	VATYAQ
BEQFUZ	EZULAM05	LISCOG	SAWLIM	VAVCUQ
CONKUN	EZULEQ03	NIJHEU	SAWLOS	VEZPIB
COTBAP	EZULIU	OLAWEE	SIHSAF	VEZQUO
DEXQAA	EZULOA04	PEDQUL	SIHSEJ	VEZRAV
DEXQEE	FANXUO	PEDRAS	TEJQOP	WOQCEK
DEXQII	GIVDOF	PEDREW	TIDWEI	XAFCEO
DEXQOO	GUBYIM	***PEKZIQ***	TISSIY	***XAXFUY***
DEXQUU	HAGVIV	***PEKZOW***	TISSOE	XOPVEE
DEXRAB	IFOWEG	PIBKIV	UHEQIH	XUPZEO
DEXRAB01	KEVQIN	***QIFPIG***	***USOJUI***	***YUBQIW***
DEXRIJ	KEVQOT	***QIFPOM***	***UZIHIV***	***ZATCAA***

Table S8 Structures in the CSD (version 5.37) containing tetrakis(urea)s. Structures consisting of linear molecules are shown in bold italics; molecules in the remaining structures are multipodal.

AMUVIO01	FEBNOR	***MAVSEJ***	OJOCOG	SUVMIG
BEPRUL	FEBNUX	***MAVSIN***	PEPNEF	***XUNFIW***
BEPSAS	FEBPAF	***MAVSOT***	PEPNIJ	***XUNFOC***
BEPSEW	FEBPEJ	MOGLIE	***PIBFIR***	***XUNFUI***
BEPSIA	***GERWAD***	NIVFOP01	***PIBFOX***	***XUNGAP***
CEXMID	JOGZIQ	***OCUNAD***	***PIBFUD***	YAKFOH
CEXMOJ	JOGZOW	***OCUNEH***	REPMAC	YAZQUL
CEXMUP	LEXMIM	***OCUNIL***	***REPQEK***	***ZATCEE***
CONLAU	***MAVROS***	***OCUNOR***	***REPQIO***	***ZATCII***
DIJRUL	***MAVRUY***	***OCUNUX***	***REPRAH***	***ZATCOO***
FEBNIL	***MAVSAF***	OJOCIA	SUVMEC	

Table S9 Structures in the CSD (version 5.37) containing oligomers with five or more urea groups. Structures consisting of linear molecules are shown in bold italics; molecules in the remaining structures are multipodal.

CUXBUT	HEKVUQ	LEYWUJ	NOQSOB	SEQGEB
CUXCAA	HEYDEV	LISCEW	NUCFIB	SEQGIF
CUXCEE	HEZLAA	LISCUM	ODAHEI	SEQGOL
CUYWOJ	IPONOQ	LISDAT	ODAHIM	SEQGUR
DEPDAE	JEXNEG	LISDEX	ODAHOS	SEQHAY
DEPDEI	JEYZET	LOFKIC	OWISIX	SETKIM
EHIXOJ	JOHYIP	LOFKOI	OWISOD	SIHRUY
EJISAS	JOHYOV	MEXCAT	PEFGUD	URIZOL
EJISIA	KAJFUY	NANZAE	PEFHAK	VEDYAF
EJISOG	KAJGAF	NANZEI	PEFHEO	VEDYOT
EJISUM	KAJGEJ	NAVWEN	PEFHIS	VEDYUZ
FELFUY	KAJGIN	NAVWIR	PEFHUE	VINBAW
FELJAJ	KAJGOT	NAVWIR02	PEFJAM	WITNIY
GEQTIH	KECHAC	NEMJOF01	PEJVAD	WITNOE
GEQTON	KIKGAO	NEMJUL	PEJVEH	WOCXIW
GEQTUT	KOLNEF	NEMKAS	PEJVOR	WOCXOC
GEQVAB	KOLNIJ	NEMKEW	PEJWAE	XAFCAK
GUBXOR	KOLNOP	NEMKIA	POQTIZ	XAFCIS
GUBXOR01	KOLNUV	NEMKOG	POQTOF	XAFCOY
GUBYAE	KURTUN	NERNOO	PUDRIQ	
GUBYEI	LEYWET	NOGSIL	PUDROW	
HAPHUD	LEYWIX	NOGXEM	PUNRUM	

Table S10 Structures in the CSD (version 5.37) containing polymeric ureas.

2.3 Crystal structure analysis

	**1a**	**1b**	**1c**	**1d**[13]	**2a** (Form 1)
Formula	$C_{16}H_{26}N_4O_2$	$C_{18}H_{30}N_4O_2$	$C_{20}H_{34}N_4O_2$	$C_{22}H_{38}N_4O_2$	$C_{20}H_{34}N_4O_2$
Formula weight	306.41	334.46	362.51	390.56	362.51
Space group	$C2/c$	$C2$	$P2$	$Pbca$	$P2_1/c$
a / Å	25.0139(13)	18.0134(12)	9.1013(9)	8.9115(2)	13.876(4)
b / Å	9.1736(3)	13.8756(7)	7.1705(9)	16.2345(3)	18.058(4)
c / Å	16.0505(7)	18.0080(13)	16.298(3)	32.4328(7)	17.452(3)
β / °	103.464(4)	117.631(9)	91.805(12)	90	93.691(15)
V / Å^3	3581.9(3)	3987.7(5)	1063.1(3)	4692.17(17)	4363.9(17)
Z	8	8	2	8	8
D_{calc} / g cm^{-3}	1.136	1.114	1.132	1.106	1.104
R_{int}	0.0463	0.0810	0.1162	0.1116	0.1102
R_1 [$I \geq 2\sigma(I)$]	0.0539	0.0881	0.0984	0.0637	0.0778
wR_2 [all data]	0.1376	0.1339	0.2247	0.1738	0.2129

	**2a** (Form 2)	**2b**	**2c**	**3**	**4** (Form 1)
Formula	$C_{20}H_{34}N_4O_2$	$C_{22}H_{38}N_4O_2$	$C_{24}H_{42}N_4O_2$	$C_{20}H_{30}N_4O_2$	$C_{20}H_{26}N_4O_2$
Formula weight	362.51	390.56	418.62	358.48	354.45
Space group	$P4_32_12$	$Pna2_1$	$P2_1$	$P2_12_12_1$	$P2_12_12_1$
a / Å	11.000(2)	9.2810(3)	9.3073(16)	10.5775(13)	15.7089(7)
b / Å	11.000(2)	16.7231(6)	23.098(4)	10.8913(11)	17.8992(8)
c / Å	18.106(4)	14.7367(5)	11.788(2)	17.9211(18)	6.9256(3)
β / °	90	90	90.039(2)	90	90
V / Å^3	2190.7(11)	2287.25(14)	2534.3(8)	2064.6(4)	1947.32(15)
Z	4	4	4	4	4
D_{calc} / g cm^{-3}	1.099	1.134	1.097	1.153	1.209
R_{int}	0.0726	0.1099	0.0463	0.0569	0.0305
R_1 [$I \geq 2\sigma(I)$]	0.0682	0.0670	0.0442	0.0573	0.0304
wR_2 [all data]	0.1835	0.1176	0.1073	0.1427	0.0769

	**4** (Form 2)	**5a** (Form 1)[14]	**5a** (Form 2)[14]	**5b** (Form 1)
Formula	$C_{20}H_{26}N_4O_2$	$C_{18}H_{28}N_4O_2Cl_2$	$C_{18}H_{28}N_4O_2Cl_2$	$C_{20}H_{32}N_4O_2Cl_2$
Formula weight	354.45	430.34	403.34	431.39
Space group	Pc	$I2/a$	$P2_12_12_1$	$Pccn$
a / Å	6.8841(6)	15.9023(7)	10.6959(6)	16.4399(18)
b / Å	8.9554(8)	9.1526(3)	10.9131(8)	30.083(3)
c / Å	16.1358(14)	30.4646(14)	18.0408(11)	9.1417(10)
β / °	90.154(3)	105.031(4)	90	90
V / Å^3	994.77(15)	4282.3(3)	2105.8(2)	4521.2(8)
Z	2	8	4	8
D_{calc} / g cm^{-3}	1.183	1.251	1.272	1.268
R_{int}	0.0421	0.0628	0.0485	0.0857
R_1 [$I \geq 2\sigma(I)$]	0.0900	0.0664	0.0475	0.1324
wR_2 [all data]	0.2537	0.1686	0.1357	0.3359

	**5b** (Form 2)	**5c**	meso-**6**	**7a**
Formula	$C_{20}H_{32}N_4O_2Cl_2$	$C_{20}H_{34}N_4O_4$	$C_{24}H_{38}N_4O_4$	$C_{24}H_{30}N_4O_4$
Formula weight	431.39	394.51	446.58	438.52
Space group	$P2_12_12_1$	$Pbca$	$P2_1/n$	$Pca2_1$
a / Å	10.9901(4)	8.9835(2)	9.3681(10)	9.0107(6)
b / Å	11.3666(4)	15.8130(5)	11.5545(13)	15.7046(11)
c / Å	18.2867(7)	31.6198(9)	22.951(3)	16.3185(11)
β / °	90	90	96.273(4)	90
V / Å^3	2284.38(14)	4491.8(2)	2469.5(5)	2309.2(3)
Z	4	8	4	4
D_{calc} / g cm^{-3}	1.254	1.167	1.201	1.261
R_{int}	0.0453	0.0526	0.0916	0.1029
R_1 [$I \geq 2\sigma(I)$]	0.0416	0.0642	0.0921	0.0593
wR_2 [all data]	0.1018	0.1319	0.2841	0.1191

	**7b** (Form 1)	**7b** (Form 2)	**9a**	**9b**
Formula	$C_{24}H_{30}N_4O_2S_2$	$C_{24}H_{30}N_4O_2S_2$	$C_{28}H_{34}N_4O_2$	$C_{30}H_{38}N_4O_2$
Formula weight	470.64	470.64	458.59	486.64
Space group	$Pccn$	$P2_1/c$	$P2_1/n$	$Pbca$
a / Å	16.6186(16)	20.8584(11)	13.7268(5)	41.500(3)
b / Å	31.589(3)	12.8685(6)	9.3304(3)	13.8787(10)
c / Å	9.2074(9)	9.1945(6)	19.7303(6)	9.2292(7)

β / °	90	97.234(5)	97.330(3)	90
V / Å^3	4833.5(8)	2448.3(2)	2506.33(14)	5315.7(7)
Z	8	4	4	8
D_{calc} / g cm^{-3}	1.293	1.277	1.215	1.216
R_{int}	0.1117	0.1131	0.0755	0.1356
R_1 [$I \geq 2\sigma(I)$]	0.0622	0.0672	0.0627	0.0617
wR_2 [all data]	0.1630	0.1555	0.1303	0.1277
	**9c**	**10a**	**10b**	**10c**
Formula	$C_{32}H_{42}N_4O_2$	$C_{28}H_{32}N_4O_2Cl_2$	$C_{28}H_{32}N_4O_2Br_2$	$C_{28}H_{32}N_4O_2Cl_2$
Formula weight	514.69	527.47	616.39	527.47
Space group	$P2_1$	$P2_1/c$	Cc	$P2_12_12_1$
a / Å	9.0390(4)	9.0025(7)	20.4713(12)	11.3349(5)
b / Å	24.3503(10)	11.9075(8)	16.1223(9)	13.8795(6)
c / Å	13.6232(5)	25.3236(17)	9.1809(5)	34.8860(13)
β / °	92.280(2)	91.255(3)	112.567(2)	90
V / Å^3	2996.1(2)	2714.0(3)	2798.1(3)	5488.4(4)
Z	4	4	4	8
D_{calc} / g cm^{-3}	1.141	1.291	1.463	1.277
R_{int}	0.0455	0.1048	0.0401	0.2337
R_1 [$I \geq 2\sigma(I)$]	0.0497	0.0469	0.0392	0.1021
wR_2 [all data]	0.1255	0.1141	0.0839	0.2662

Table S11 Summary of crystallographic data for bis ureas in this study. Data for **1d** and **5a** have been published previously but are included for comparison.[13-14]

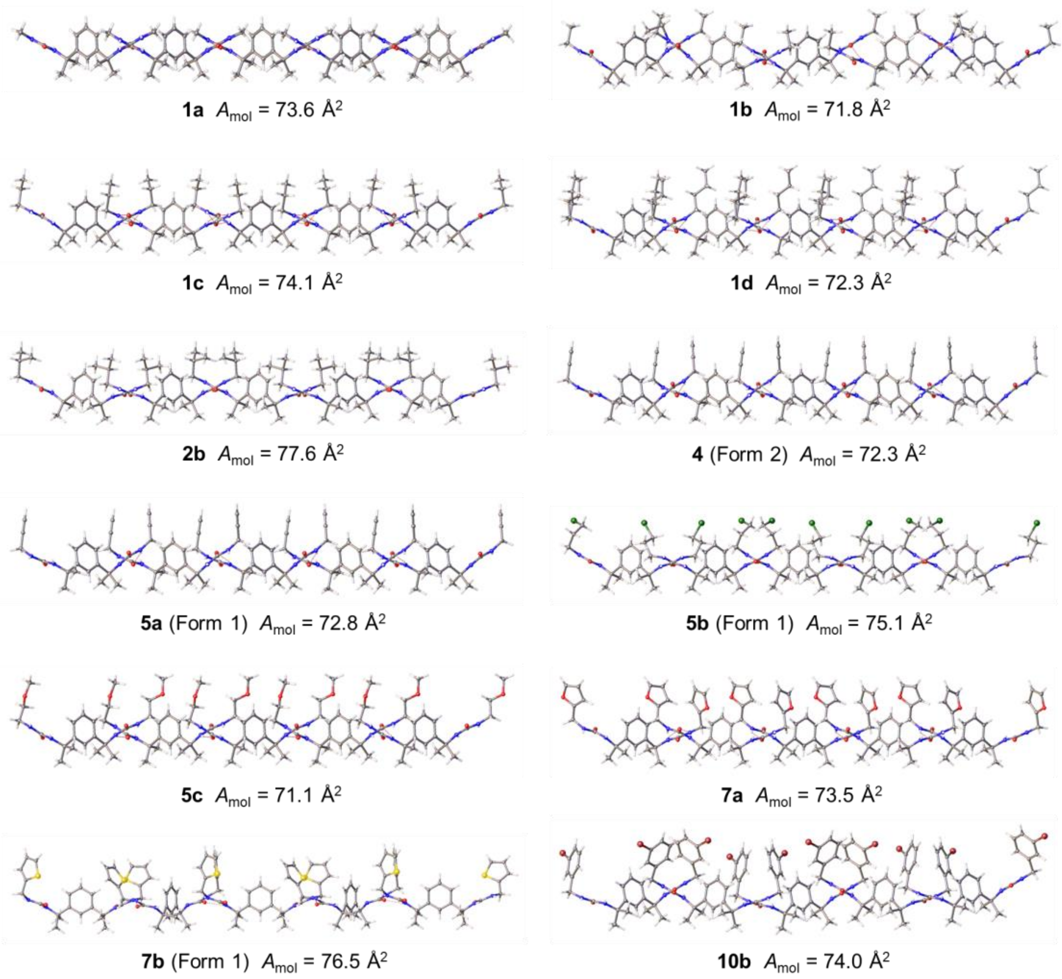

Figure S94 Molecular packing and A_{mol} values of asymmetric lamellae in bis(urea) crystal structures, viewed down the α-tape axes. All structures feature an [AB] α-tape network topology in which neighboring tapes are antiparallel.

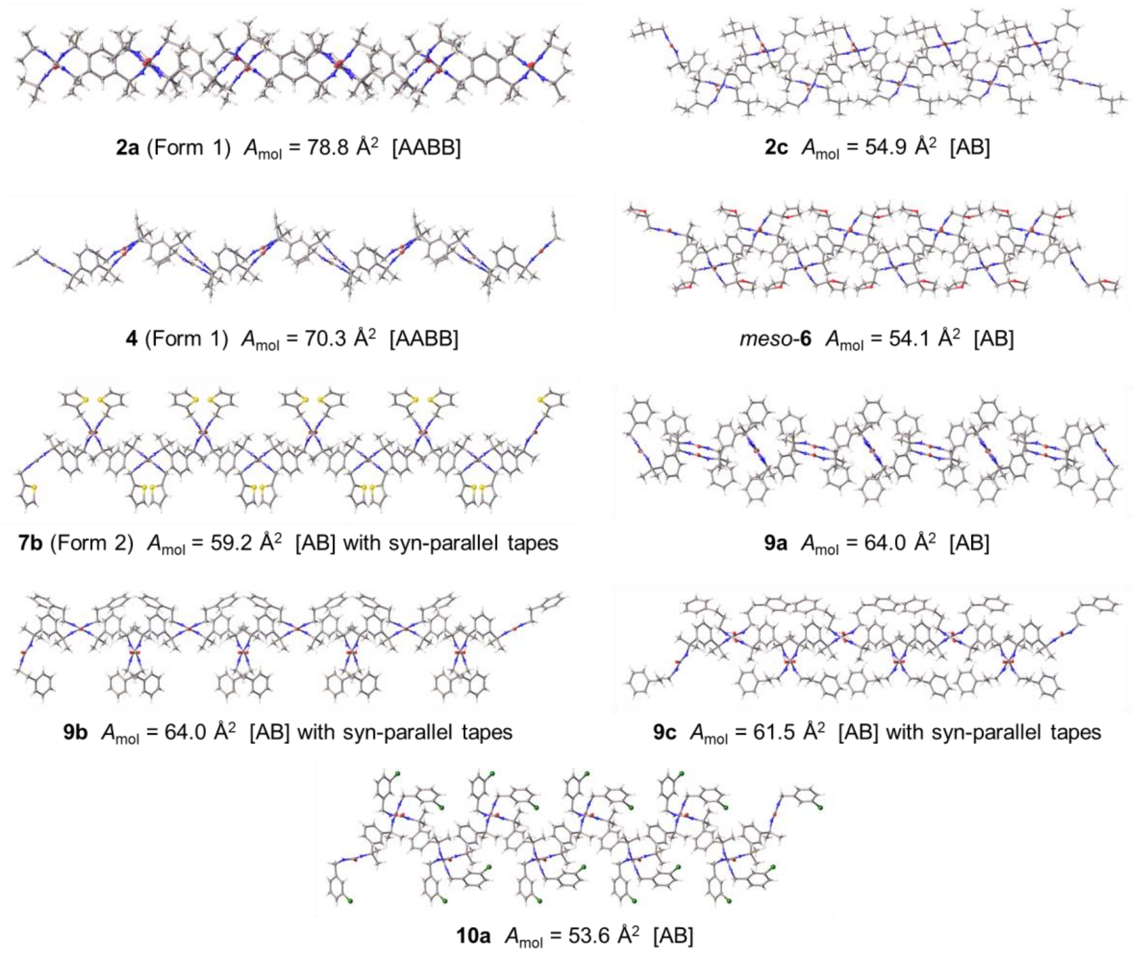

Figure S95 Molecular packing, A_{mol} values and α-tape network topologies of symmetric lamellae in bis(urea) crystal structures, viewed down the α-tape axes. Note that the lamellae of **7b** (Form 2), **9b** and **9c** are geometrically asymmetric, as the bis(urea) end groups on the upper and lower faces adopt different conformations. Neighboring tapes are antiparallel unless otherwise stated.

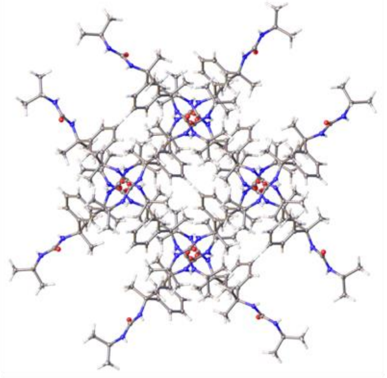

2a (Form 2) [ABCD]

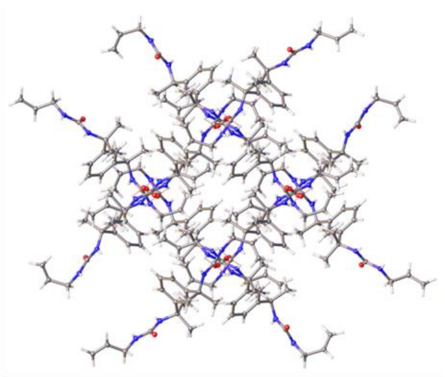

3 [ABCD]

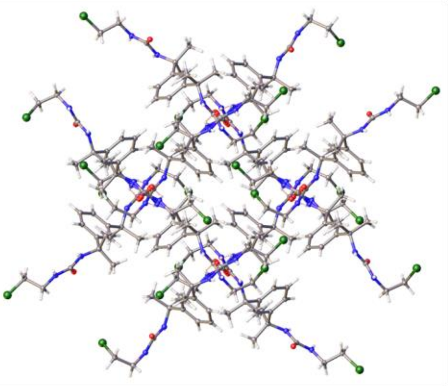

5a (Form 2) [ABCD]

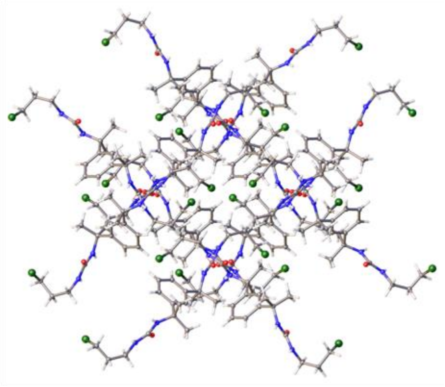

5b (Form 2) [ABCD]

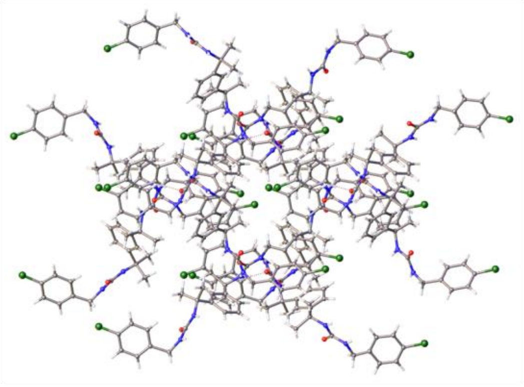

10c [ABABCDCD]

Figure S96 Molecular packing and α-tape network topologies of non-lamellar bis(urea) crystal structures, viewed down the α-tape axes. Neighboring tapes are antiparallel in all structures.

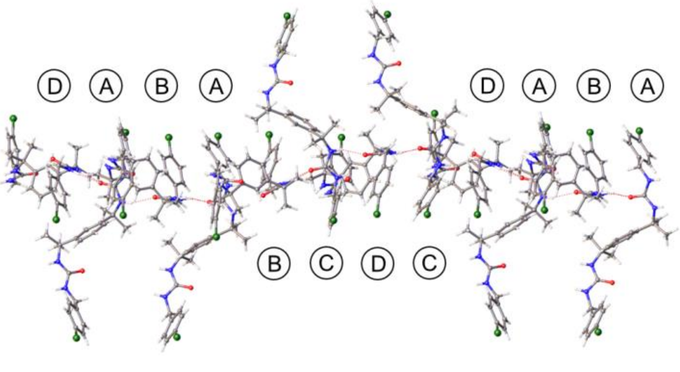

Figure S97 The [ABABCDCD] α-tape network formed by **10c**.

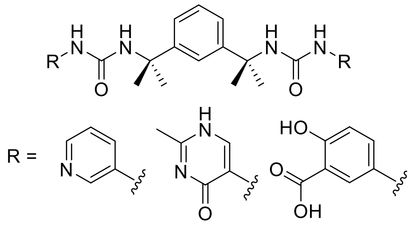

Figure S98 Bis(urea)s based on tetramethylxylylene diisocyanate with previously reported crystal structures. The CSD refcodes of the structures are GUDKAR, GUDKEV, JEMQOI, NEJVIJ, NUTFOZ, NUTFUF, RUSLAU, QENGAS, QENGEW, URIZIF, URIZOL, XOPTUS, XOPVAA and XOPVEE.

2.4 Gel characterization

Rheometry

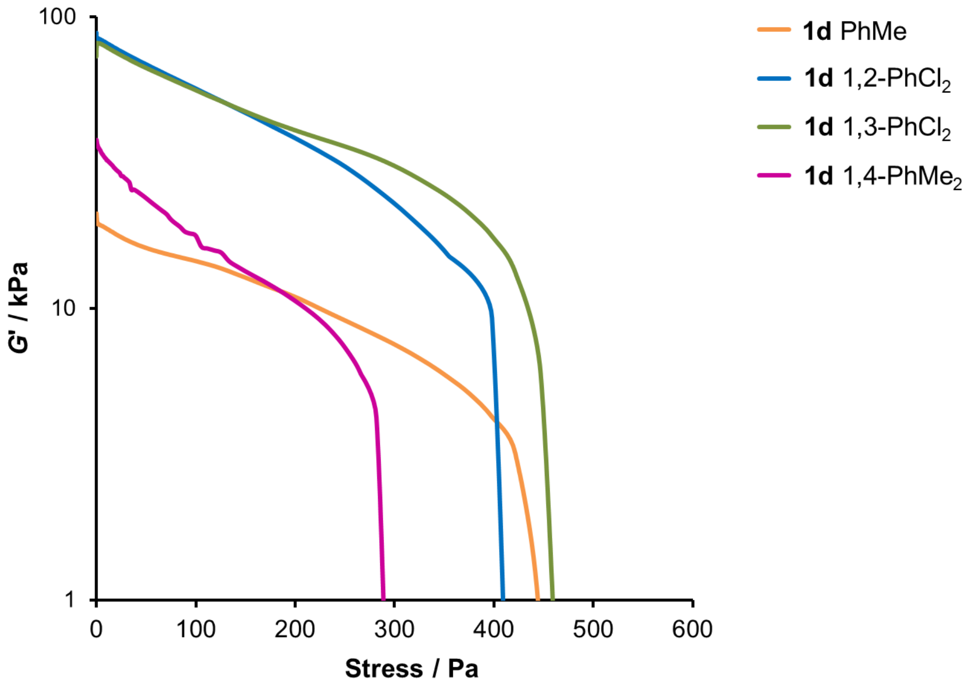

Figure S99 Variation of *G*′ and *G*″ with shear stress for gels of **1d** in four different solvents. All gels were prepared from 1% (w/v) solutions and analyzed with a constant frequency of 1 Hz. The gel of p-xylene (1,4-PhMe) was sheared at 20°C to prevent freezing of the solvent (melting point 13.2°C), while the remaining experiments were performed at 10°C.

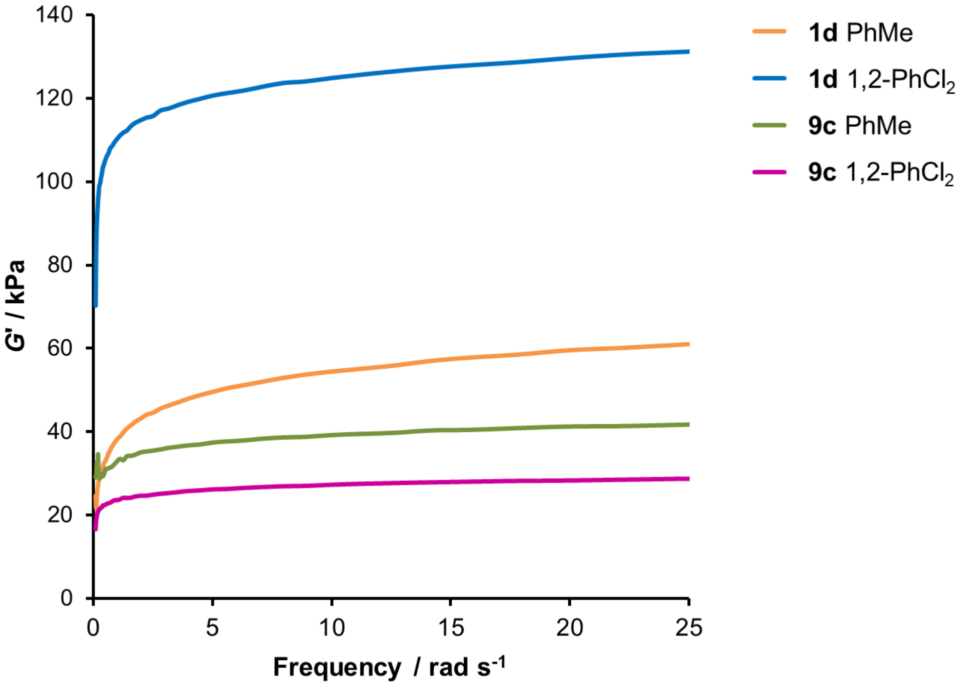

Figure S100 Variation of *G*′ with shear frequency for gels of **1d** and **9c** in toluene (PhMe) and 1,2-dichlorobenzene (1,2-$PhCl_2$). All gels were prepared from 1% (w/v) solutions and analyzed at 10 °C with a constant shear stress of 1 Pa.

Scanning electron microscopy

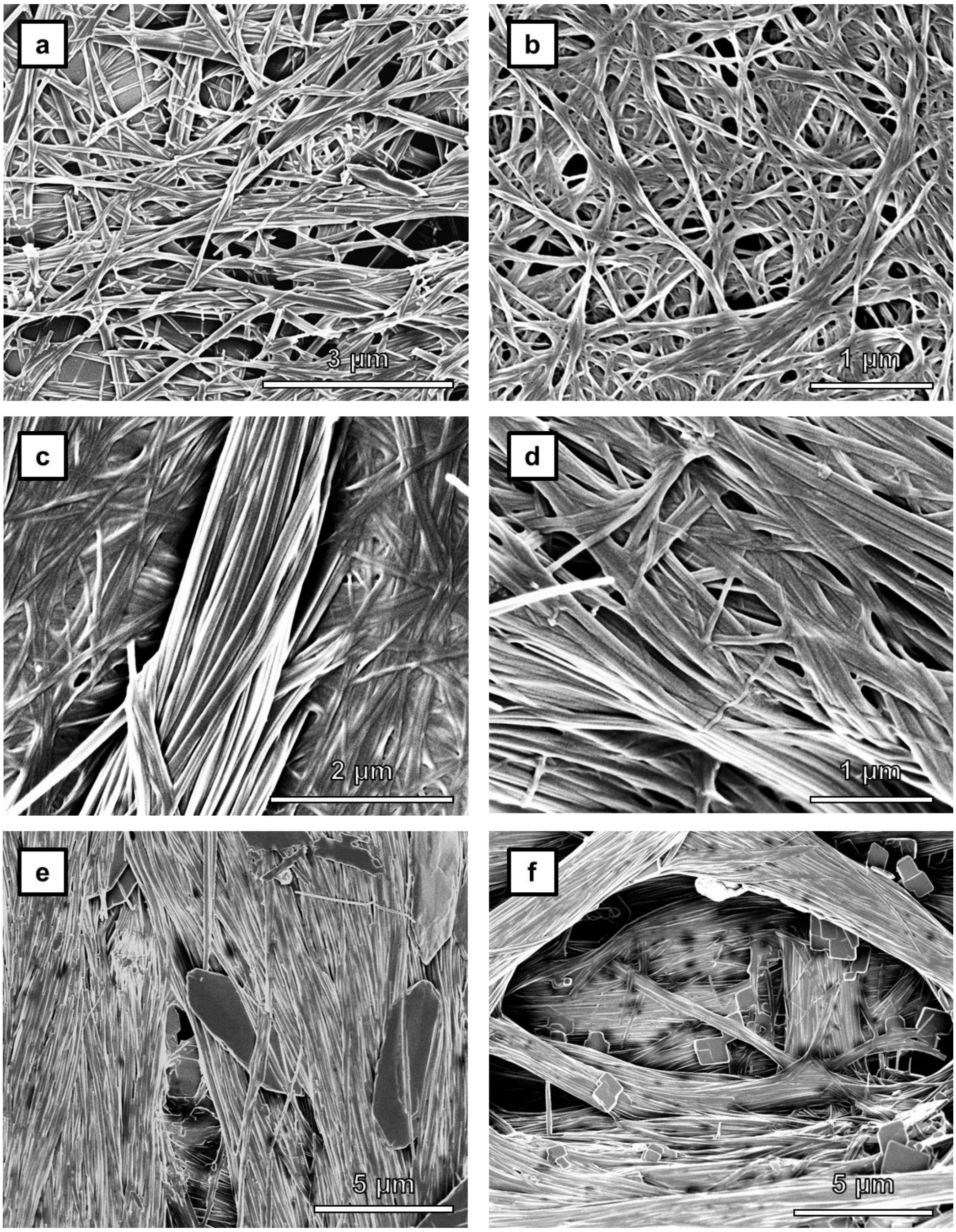

Figure S101 SEM micrographs of xerogels prepared from 1% (w/v) gels of (a) **1a** in nitrobenzene, (b) **4** in nitrobenzene, (c) **6** in 1,2-dichlorobenzene, (d) **6** in nitrobenzene, (e) **7a** in 1,2-dichlorobenzene and (f) **10c** in 1,2-dichlorobenzene.

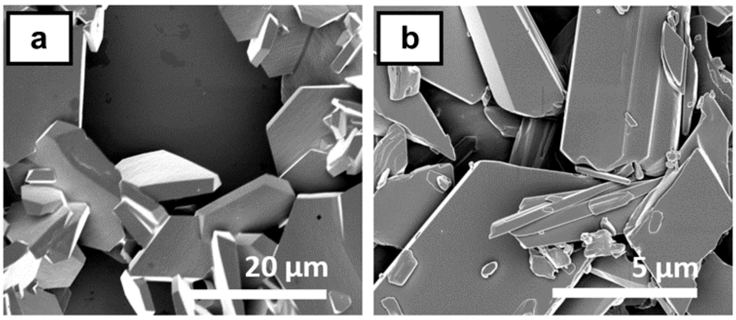

Figure S102 SEM micrographs of precipitates prepared by drying 1% (w/v) gels of (a) **7b** and (b) **8** in nitrobenzene. The images suggest that recrystallization occurs during drying of the samples.

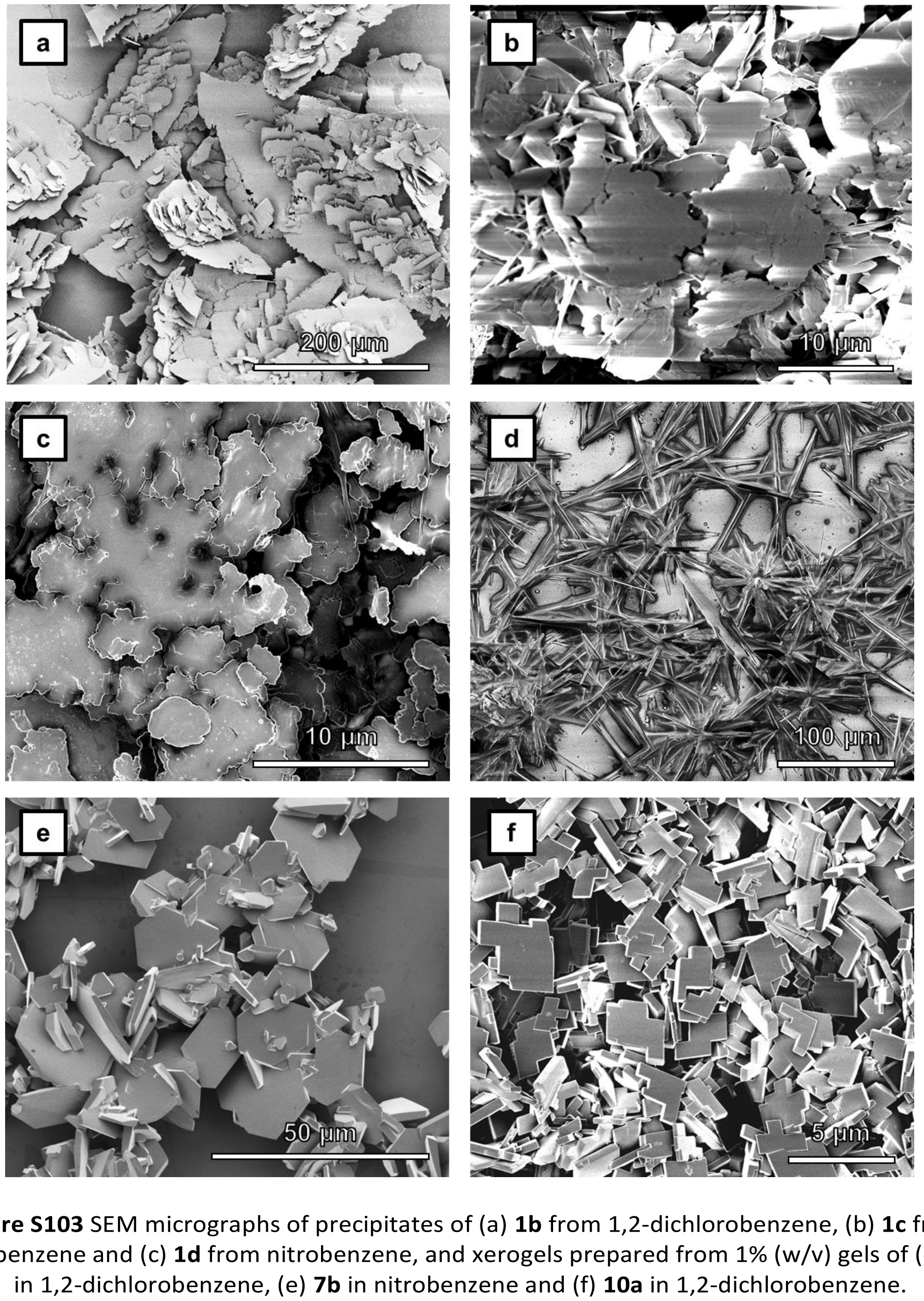

Figure S103 SEM micrographs of precipitates of (a) **1b** from 1,2-dichlorobenzene, (b) **1c** from nitrobenzene and (c) **1d** from nitrobenzene, and xerogels prepared from 1% (w/v) gels of (d) **7b** in 1,2-dichlorobenzene, (e) **7b** in nitrobenzene and (f) **10a** in 1,2-dichlorobenzene.

2.5 Molecular dynamics

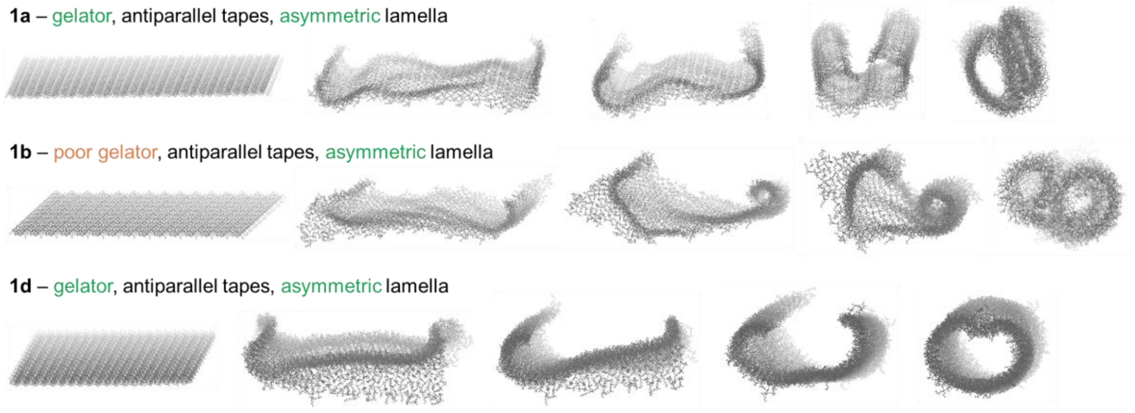

Figure S104 Selected frames from 500 ps MD simulations of lamellae extracted from the crystal structures of **1a**, **1b** and **1d**.

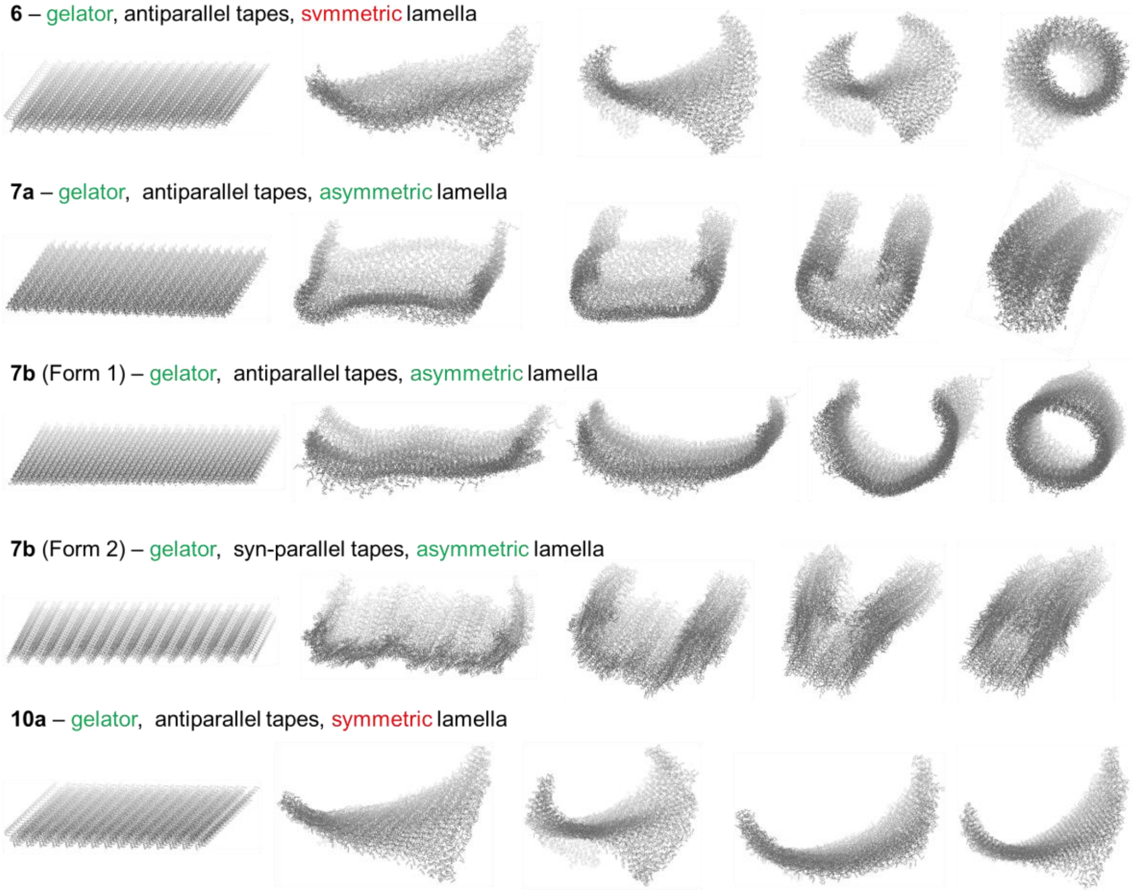

Figure S105 Selected frames from 500 ps MD simulations of lamellae extracted from the crystal structures of **6**, **7a**, **7b** and **10a**.

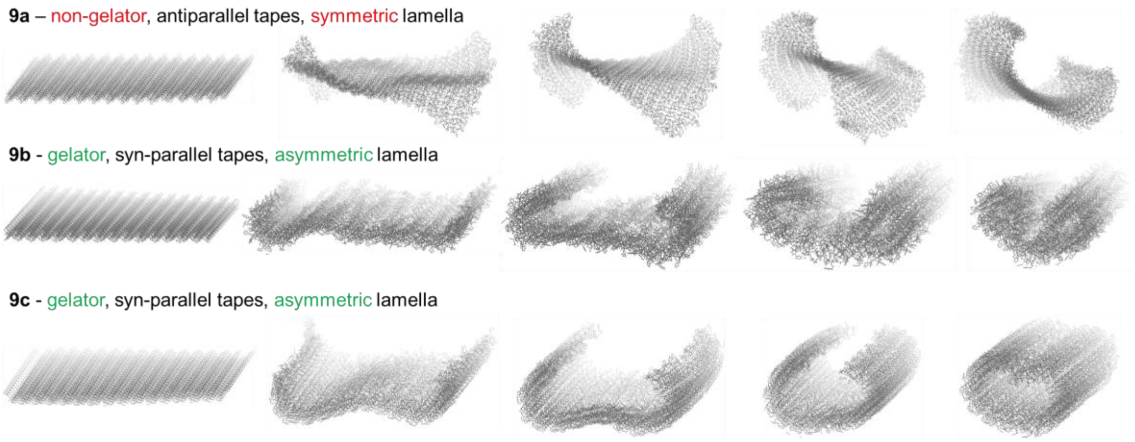

Figure S106 Selected frames from 500 ps MD simulations of lamellae extracted from the crystal structures of **9a**, **9b** and **9c**.

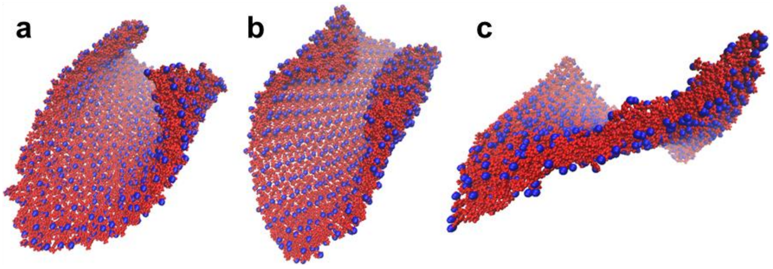

Figure S107 Final frames from 500 ps MD simulations of non-scrolling lamellae in a vacuum at 300 K. The lamellae consist of molecules of (a) **2c**, (b) **9a** and (c) **10a**, and were simulated from initial coordinates extracted from the crystal structures of **2c**, **9a** and **10a**, respectively. One atom in each end group is shown as a blue sphere of radius 1.3 Å and all other atoms are shown as red spheres of radii 0.7 Å.

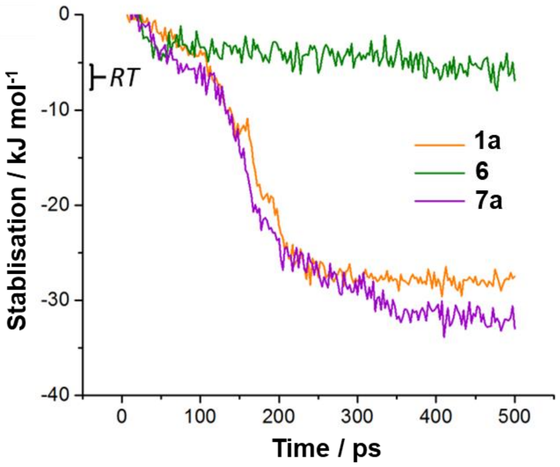

Figure S108 Energy profiles for MD simulations of scrolling in the asymmetric lamellae of **1a** and **7a** and random folding in the symmetric lamella of *meso*-**6**. An energy interval corresponding to *RT* at 300 K is labelled for comparison.

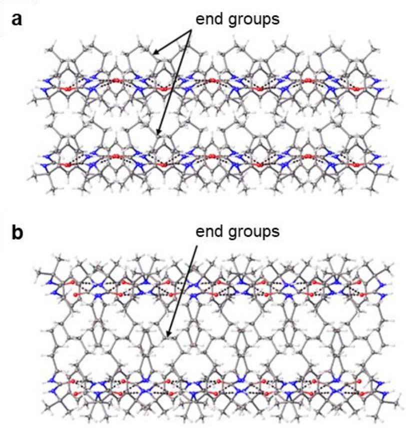

Figure S109 (a) Polar stacking in the structure of **1c**; (b) non-polar stacking in the structure of **1d**.

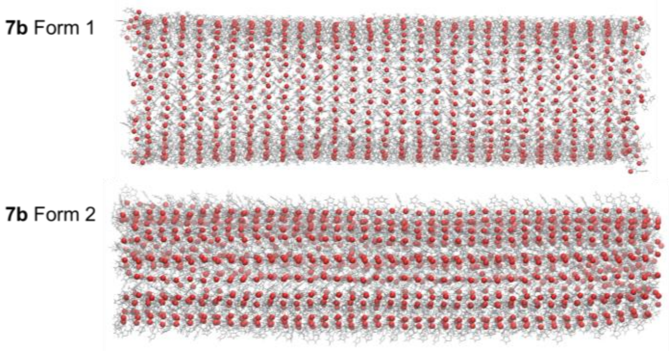

Figure S110 Final frames from 500 ps MD simulations of lamellae extracted from the crystal structures of **7b**. Oxygen atoms are shown in red to highlight the direction of the α-tape axes in the two scrolled structures.

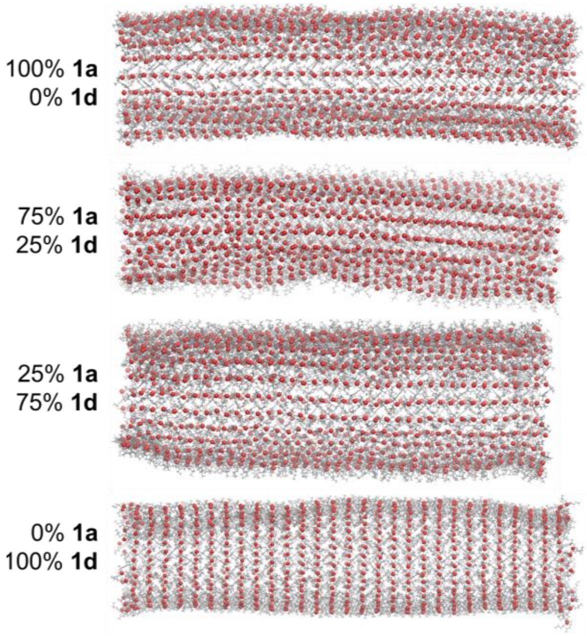

Figure S111 Final frames from 500 ps MD simulations of lamellae extracted from the crystal structures of **1a** and **1d**. In the simulations featuring both bis(urea) analogues, the initial lamellae were constructed by randomly replacing molecules in the lamella of **1d** with molecules of **1a**. Oxygen atoms are shown in red to highlight the direction of the α-tape axes in the final structures.

3. References

(1) Sheldrick, G. M., SHELXT - Integrated space-group and crystal-structure determination. *Acta Crystallogr. Sect. A* 2015, *71*, 3-8.

(2) Dolomanov, O. V.; Bourhis, L. J.; Gildea, R. J.; Howard, J. A. K.; Puschmann, H., OLEX2: a complete structure solution, refinement and analysis program. *J. Appl. Cryst.* 2009, *42*, 339-341.

(3) Van der Spoel, D.; Lindahl, E.; Hess, B.; Groenhof, G.; Mark, A. E.; Berendsen, H. J. C., GROMACS: Fast, flexible, and free. *J. Comput. Chem.* 2005, *26*, 1701-1718.

(4) Case, D. A.; Cheatham, T. E.; Darden, T.; Gohlke, H.; Luo, R.; Merz, K. M.; Onufriev, A.; Simmerling, C.; Wang, B.; Woods, R. J., The Amber biomolecular simulation programs. *J. Computat. Chem.* 2005, *26*, 1668-1688.

(5) Wang, J. M.; Wang, W.; Kollman, P. A.; Case, D. A., Automatic atom type and bond type perception in molecular mechanical calculations. *J. Mol. Graphics Modell.* 2006, *25*, 247-260.

(6) Jakalian, A.; Bush, B. L.; Jack, D. B.; Bayly, C. I., Fast, efficient generation of high-quality atomic Charges. AM1-BCC model: I. Method. *J. Comput. Chem.* 2000, *21*, 132-146.

(7) Berendsen, H. J. C.; Postma, J. P. M.; Vangunsteren, W. F.; DiNola, A.; Haak, J. R., MOLECULAR-DYNAMICS WITH COUPLING TO AN EXTERNAL BATH. *J. Chem. Phys.* 1984, *81*, 3684-3690.

(8) Jones, C. D.; Kennedy, S. R.; Walker, M.; Yufit, D. S.; Steed, J. W., Scrolling of Supramolecular Lamellae in the Hierarchical Self-Assembly of Fibrous Gels. *Chem* 2017, *3*, 603-628.

(9) Peng, C. Y.; Ayala, P. Y.; Schlegel, H. B.; Frisch, M. J., Using redundant internal coordinates to optimize equilibrium geometries and transition states. *J. Comput. Chem.* 1996, *17*, 49-56.

(10) Dunning, T. H., gaussian-basis sets for use in correlated molecular calculations .1. The atoms boron through neon and hydrogen. *J. Chem. Phys.* 1989, *90*, 1007-1023.

(11) Hehre, W. J.; Ditchfield, R.; Pople, J. A., Self-consistent molecular-orbital methods .12. Further extensions of gaussian-type basis sets for use in molecular-orbital studies of organic-molecules. *J. Chem. Phys.* 1972, *56*, 2257-2261.

(12) Groom, C. R.; Bruno, I. J.; Lightfoot, M. P.; Ward, S. C., The Cambridge Structural Database. *Acta Crystallogr. Sect. B* 2016, *72*, 171-179.

(13) Dawn, A.; Mirzamani, M.; Jones, C. D.; Yufit, D. S.; Qian, S.; Steed, J. W.; Kumari, H., Investigating the effect of supramolecular gel phase crystallization on gel nucleation. *Soft Matter* 2018, *14*, 9489-9497.

(14) Jones, C. D.; Cook, L. J. K.; Marquez-Gamez, D.; Luzyanin, K. V.; Steed, J. W.; Slater, A. G., High-Yielding Flow Synthesis of a Macrocyclic Molecular Hinge. *J. Am. Chem. Soc.* 2021, *143*, 7553-7565.
